# Detection of non-invasive sexing of early chick embryos in intact eggs using laser speckle contrast imaging and deep neural networks

**DOI:** 10.1371/journal.pone.0323847

**Published:** 2026-06-26

**Authors:** Simon Mahler, Anika Arora, Carol Readhead, Siyuan Yin, Surya Narayanan Hari, Ellie Wang, Cecilia I. Moxley, Abdullahi A. Adeboye, Zhenyu Dong, Haowen Zhou, Xi Chen, Marianne Bronner, Changhuei Yang

**Affiliations:** 1 Department of Electrical Engineering, California Institute of Technology, Pasadena, California, United States of America; 2 Department of Biomedical Engineering, Stevens Institute of Technology, Hoboken, New Jersey, United States of America; 3 California Institute of Technology, Pasadena, California, United States of America; 4 Department of Biology and Biological Engineering, California Institute of Technology, Pasadena, California, United States of America; 5 Department of Medical Engineering, California Institute of Technology, Pasadena, California, United States of America; Islamia University of Bahawalpur: The Islamia University of Bahawalpur Pakistan, PAKISTAN

## Abstract

The ability to image blood flow in early-stage avian embryos has significant applications in developmental biology, drug and vaccine testing, as well as determining sex differentiation. In this project, a recently developed laser speckle contrast imaging (LSCI) system was used to non-invasively image extraembryonic blood vessels and used these images to attempt early sex identification of chick embryos. Specifically, blood vessels images were captured from 1,251 living chicken embryos between day three and day four of incubation. Then, deep neural network (DNN) models were applied to evaluate whether it is possible to differentiate sex based on vascular patterns. Using ResNetBiT and YOLOv5s-cls models, our results indicate that sex differentiation from extraembryonic blood vessel images was not achievable with sufficiently high accuracy or statistical significance for practical use. Specifically, ResNetBiT had a five-fold cross-validated average accuracy of 56% ± 4% (p-value of 0.28 across cross-validation folds) at day 3 and 57% ± 3% (p-value of 0.07 across cross-validation folds) at day 4. YOLOv5s-cls had a five-fold cross-validated average accuracy of 55% ± 2% (p-value of 0.13 across folds) at day 3 and 57% ± 3% (p-value of 0.10 across folds) at day 4. Our findings suggest that under the current experimental conditions and modeling approaches, per-egg evaluation did not produce sufficiently accurate or statistically robust results for early sex classification.

## Introduction

Incubating fertile chicken eggs is a crucial process in the poultry industry for producing male (cockerels) and female (hens) chicks. Hens are used for meat production and egg-laying. Cockerels can only be used for meat production, but they are generally considered economically unviable for such purpose as they grow more slowly and do not reach the same size as hens [1]. Consequently, cockerels are routinely killed immediately after sex determination (male chick culling) on the same day they hatch [2–6].

One prevalent method of culling is maceration, which involves grinding the chicks alive [4]. Other methods include gassing and cervical dislocation [5,6]. All those methods are cruel and ethically questionable due to the distress and suffering they inflict on the chicks. In addition, the newly hatched chicks have to be manually sexed which is laborious and expensive. More than seven billion male chicks are culled annually around the world [1,7]. This grim reality underscores the urgent need for reevaluation and innovation within the poultry industry to address both economic and ethical concerns associated with the treatment of male chicks.

A straightforward solution to prevent mass male chick culling is to selectively incubate only female chicken eggs. However, no effective methods currently exist to accurately identify the sex of a chicken egg before incubation [8]. An alternative approach is to incubate the eggs and apply a non-invasive sexing technology within the first few days of development. At this stage, the embryo has not yet developed a functional sensory nervous system [4,9], making it an ethical window for intervention. In addition, male chick embryos could be redirected for alternative uses such as animal research or breeding. There exist methods for early-stage sexing of avian eggs, however, they require breaking the eggshell [8,10–12] and negatively impacting the natural development of the eggs. A robust and commercially viable sexing technology should be non-invasive and should not alter egg development. There exist different non-invasive optical methods for in ovo sexing of chicken embryos including visible and near-infrared spectroscopy, hyperspectral imaging, Raman spectroscopy, and fluorescence spectroscopy [13–15].

Recently, deep neural network (DNN) based studies have reported positive sex identification results using egg candling images of chick embryos [8,14,16–20]. Egg candling is a well-established technique in which an egg is held up to a strong light source to reveal its blood vessel network due to the high optical absorption of hemoglobin (in contrast to laser speckle contrast imaging (LSCI) [21,22] that provides blood flow). A recent study [16] suggest that DNN models can pick-up the subtle sex-based differences in the blood vessel network at early stages (as early as day 3 of incubation). However, these results are not supported by biological plausibility. Studies on embryonic gene expression indicate that key sex-determining genes begin to show differential expression around day 4.5 of incubation in male embryos and around day 6.5 in female embryos [23], with morphological differentiation in the gonads becoming apparent only after day 6.5. These developmental constraints suggest that pronounced sex-specific features are unlikely to be present at earlier stages. Nevertheless, for this study, we hypothesized the following: *subtle sex-related differences in vascular patterns may emerge prior to genetic expression,* potentially enabling sex classification through blood vessels image analysis.

We recently developed an improved egg imaging system using laser speckle contrast imaging (LSCI) [21,22]. Compared to traditional egg candling, this approach offers superior detection of active blood flow in blood vessels and is robust against eggshell coloration variations and stains [21]. Blood vessel images acquired with our LSCI system would serve as better input for DNN-based image analysis. This study aims to verify the following hypothesis: *LSCI-derived vascular patterns contain sufficient discriminative information for reliable sex determination at early developmental stages.*

In this paper, a non-invasive LSCI system was used to capture images of the active extraembryonic blood vessels of chicken embryos between day 3 and day 4 of incubation. Blood vessel images were recorded from 1,251 chicken eggs. The eggs were then re-incubated until day 11, at which point the sex was confirmed by breaking the eggs to directly examine the gonads and confirm the sex genetically by PCR. Using the collected dataset, two DNN models were trained (ResNetBiT and YOLOv5) for the task of identifying the sex of the chick eggs based on blood vessel images. Our results, from a five-fold cross-validation using ResNetBiT or YOLOv5s-cls DNNs, suggest that the model may have some capabilities to differentiate between male and female LSCI images but not at a high enough level for practical application. ResNetBiT achieved an average accuracy of 56% at day 3 (p-value of 0.28 across cross-validation folds) and 57% at day 4 (p-value of 0.10 across folds). YOLOv5, achieved an average accuracy of 55% at day 3 (p-value of 0.13 across folds) and 57% at day 4 (p-value of 0.10 across folds). These results are in disagreement with those obtained using a similar protocol in Ref. [16], where an accuracy of around 85% was achieved with egg candling images of comparable dataset size, and similar algorithm (YOLOv5).

## Materials and methods

### Laser Speckle Contrast Imaging (LSCI) of chick embryo blood vessels

**Fig 1:**
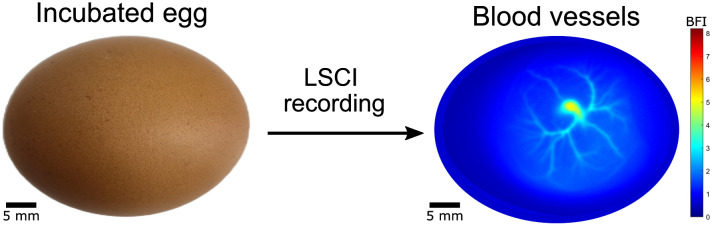
Representative LSCI image of flowing blood vessels in a chick embryo, obtained non-invasively through the eggshell. See online version for best visualization of the blood vessel image.

Fig 1 presents a typical image acquired with our laser speckle contrast imaging (LSCI) system [21,22,24], which operates based on laser speckles generated by the scattering of coherent laser light within the sample [24–30]. A laser speckle pattern is characterized by a random granular distribution of bright and dark spots. In our case, the laser speckles result from the self-interference of the scattered laser light by the eggshell and various internal components of the egg including red blood cells flowing through the blood vessels, the yolk, the embryo, and the albumen [21,24,31–33]. See Refs. [21,22] for more details on LSCI applied to image the chick embryo blood vessels.

As components such as blood move within the egg, the speckle field undergoes temporal changes. Since blood flows at a higher speed compared to other components of the egg, the temporal speckle variations are primarily influenced by blood movement. In our case, LSCI is accomplished by illuminating the egg with coherent laser light from the side of the egg and collecting the scattered light from the top of the egg with a high-resolution camera [21,22].

Speckle contrast, which typically ranges from 0 to 1, quantifies intensity variations within a speckle pattern. As blood cells move within blood vessels, their motion generates multiple speckle realizations during the camera’s exposure time. When these fluctuations occur faster than the integration time of a single image frame, they are averaged out, leading to a smeared and washed-out speckle pattern recorded by the camera. In contrast, when fluctuations are much slower than the integration time, the speckle pattern remains more defined, and pixel intensity readings reflect greater variation. Therefore, lower speckle contrast corresponds to regions with faster motion, such as flowing blood, whereas higher speckle contrast indicates static or slow-moving regions. Speckle contrast is mathematically defined as the ratio of the standard deviation to the mean value of pixel intensities, Eq. (1).

In our LSCI system (Supporting S1 Fig), the laser light source was a single-frequency continuous-wave 852 nm laser [Spectra-Physics DL852–300-SO] with an output power of 230 mW. The imaging setup included a 12.3-megapixel CMOS camera [Thorlabs CS126CU] with a pixel size of 3.45 × 3.45 µm^2^, operating at a frame rate of 21 frames per second and a pixel bit-depth of 12 bits. The camera exposure time was set to 10 ms. A 50 mm focal length lens [Edmund Optics #86–574] together with the camera formed an imaging system, achieving a magnification ratio of 0.2 and a numerical aperture of 0.03. This configuration resulted in a speckle field at the camera where, on average, a speckle would span 2 pixels laterally. The camera’s quantum efficiency at the laser wavelength of 852 nm was approximately 20%. In future studies, we plan to utilize a camera with higher quantum efficiency and a faster frame rate. See Refs. [21,22] and Supporting S1 Fig for more details on the experimental arrangement. Note that our LSCI system can also monitor blood flow time trace, with similar temporal resolution and sensitivity as speckle contrast optical spectroscopy (SCOS) [21,34,35].

Since the movement of blood flow within the vessels is significantly greater than that of other egg components, a distinct contrast difference emerges between the blood vessels and surrounding structures. This presents a key distinction from the traditional egg candling method, which relies on brightfield imaging to measure differences in light absorption between blood vessels and other components [8,16,21]. Unlike brightfield imaging, which detects absorption contrasts, LSCI selectively differentiates dynamic components from static ones [21]. Consequently, if a blood vessel is inactive (i.e., without flowing blood), it would not appear in LSCI imaging but would still be visible in brightfield imaging. This fundamental difference underscores the unique capability of LSCI in assessing blood flow dynamics. A detailed comparison study between the two methods can be found in [21] and reported that both LSCI and egg candling modalities provide comparable levels of vascular structural detail.

For this study, temporal LSCI [36,37] was used, where the camera recorded a sequence of N = 100 speckle frames images when the laser was on and another 100 noise frames $\tilde{I}(\text{t})$ when the laser was blocked. Both sets had the same exposure time T. The noise frames were averaged into a single frame and subtracted from the speckle frames [21]. The temporal speckle contrast $K_{t}$ was calculated for each camera pixel $\tilde{I}(i,j;t)$ in the temporal domain over the N noise-subtracted speckle frames as [21,22]:


$$\begin{array}{c} K_{t}(i,j)=\frac{\sigma_{t}}{\mu_{t}\;}=\frac{\sqrt{\frac{N}{N-\text{1}}(<{\tilde{I}}^{\text{2}}(i,j;t){>}_{t}-{<\tilde{I}(i,j;t){>}_{t}}^{\text{2}})}}{<\tilde{I}(i,j;t){>}_{t}\;}, \end{array}$$ (1)

where $\tilde{I}(i,j)$ is the intensity at the pixel row i and column j, t is the time at which the speckle pattern was recorded by the camera, and ${<>}_{t}$ indicates temporal averaging over time occurring at pixel i,j. The speckle contrast corresponds to the ratio between the standard deviation $\sigma_{t}$ and the temporal mean $\mu_{t}$ of the N recorded speckle pattern images. The relative blood flow index (BFI) was estimated from the speckle contrast as $BFI=\;\frac{\text{1}}{K^{\text{2}}}$ [34,38–40]. Note that this estimation is valid when the exposure time of the camera is much longer than the speckle field decorrelation time and rely on the assumption that the motion of multiple scattering blood cells is unordered [39]. It also assumes that the scattered light is an ergodic field with the absence of static scattering. These considerations are extensively studied in Ref. [41], as the different scattering regimes and particle motion types can change the form of the field correlation function $g_{\text{1}}(\tau )$ [39,41,42]. See Supporting Information A of Ref. [21] and Ref. [39] for more analysis on the blood flow index calculation from speckle contrast images in our LSCI system. The blood flow index provides relative information on blood flow, accounting for the total volume of blood moved within a given time period, differing from blood volume [43,44]. Fig 1 shows a typical example of an egg’s blood vessel image with a clear visualization of the blood vessel network. Our method can image blood vessels as small as 100 µm, see Ref. [21]. See Supporting S2 and S3 Figs for more examples of egg blood vessel images at day 3 and 4 of incubation.

### Egg incubation

For this study, brown fertile eggs were obtained from Sun Valley Farms and Petaluma Poultry in California. The eggs were incubated using Hethya, HHD, and Kebonnixs commercial incubators, which automatically maintained the temperature inside the incubator between 37–38.5°C (99–101°F) and gently rotated the eggs every 60–90 minutes. Humidity levels were kept within a 50–75% range. After three days of incubation, embryos that had developed beyond stage HH17 were selected for imaging. All animal research procedures were approved by the Caltech Office of Laboratory Animal Resources. No eggs were incubated beyond day 12, and embryo disposal was conducted in compliance with the Caltech Institutional Animal Care and Use Committee (IACUC) policies.

### Speckle imaging data collection protocol and ground-truth sex

Our data collection protocol was as follows: first, fertile eggs were incubated in a large incubator capable of holding up to 60 chicken eggs. Each egg was assigned a unique number between 1 and 1,500 in chronological order (i.e., the first egg was labeled 1, the second 2, and so on). The label was written with a black permanent marker on the rounded bottom part of the egg. The incubator automatically maintained the internal temperature and gently rotated the eggs. Distilled water was added whenever the humidity level dropped below 55%.

**Fig 2:**
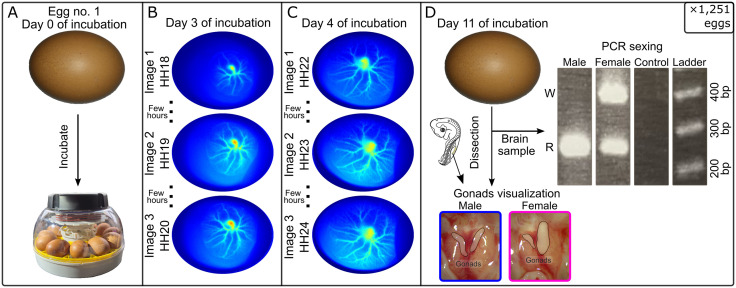
Speckle imaging data collection protocol for a single egg. **(A)** A fertile egg (e.g., Egg 1) was incubated in a large incubator. **(B)** After three days of incubation, the egg was imaged using our LSCI system. **(C)** After four days of incubation, the egg was imaged again using our LSCI system. **(D)** After eleven days of incubation, the eggshell was broken, and the developing chick embryo was dissected to visualize the gonads for sex identification. At the same time, a brain sample was collected and frozen at −20^0^C for PCR genetic testing to confirm the visual sex identification. Male and female examples are shown for comparison. See online version for best visualization of the blood vessel images.

Second, after three days of incubation, LSCI imaging (as in Fig 1) was performed at different developmental Hamburger and Hamilton (HH) [40] stages of HH18 (68 hours of incubation), HH19 (72 hours), and HH20 (75 hours) (Fig 2). After each imaging session, the egg was returned to the incubator. To prevent the same egg from being used in both training and validation within a single DNN experiment, LSCI images from the same egg were used exclusively for either training or validation.

Third, after four days of incubation, each egg was LSCI imaged at different developmental stages of HH22 (92 hours), HH23 (96 hours), and HH24 (100 hours) (Fig 2). If the imaging quality was insufficient to distinguish blood vessels from the background, the LSCI images were discarded. Due to time constraints and imaging quality selection, most eggs were only imaged at one or two developmental stages. After imaging, the eggs were returned to the incubator until day 11.

Lastly, after eleven days of incubation, the eggshell was broken, and the chick embryo was carefully dissected so that the gonads could be visualized for sex identification [45,46]. An incision was made on the ventral surface of the embryo, and the gonads were exposed (see Fig 2D insets). In males, the left and right gonads were typically similar in size and were thin, elongated structures [45, 46]. In females, the left gonad appeared larger and more elongated than the right gonad. The images were recorded using a 0.7X-5X magnification ratio microscope (AmScope H800 Series). The sex of the embryo was further validated by using PCR [47]. For the PCR analysis, approximately 50 mg of soft brain tissue was collected at the time of dissection and stored at −20°C. (Fig 2D insets). The tissue was extracted using Thermo Scientific™ Animal Tissue Direct PCRKit according to the manufacturer’s instructions. The primers for the PCR reaction were the W-repeat (W), which is specific to female DNA and R the 18S ribosomal gene which is present in both sexes Ref. [47]. The primers used were W from the female specific W chromosome which was designed to amplify a 415 Bp product, and R from the 18S ribosomal gene, which were designed to amplify a 256 Bp product Ref. [47].

The primers were:

W 5´ primer: 5´CCCAAATATAACACGCTTCACT 3´,
W 3´ primer: 5´ GAAATGAATTATTTTCTGGCGAC 3´,
R 5´ primer: 5´ AGCTCTTTCTCGATTCCGTG 3´,
R 3´ primer: 3´ GGGTAGACACAAGCTGAGCC 3´.

Fig 2D presents a typical visualization of gonads alongside PCR results for both sexes. The PCR products were run on a 1.5% agarose gel. In females, both the W and R products are detected at 415 and 256 bp, respectively, whereas in males, only the R sequence is present. On average, there was a 2% discrepancy between the sex identification via PCR and gonadal visualization. LSCI images from these eggs were discarded to maintain consistency within the dataset.

### Dataset overview: LSCI images and egg count

Following the LSCI imaging protocol (Fig 2), a total of 2,773 LSCI images were collected from 1,251 eggs. Table 1 summarizes the distribution of images and eggs across incubation days and sex categories. On day 3, covering developmental Hamburger and Hamilton (HH) [48] stages HH18 to HH20, a total of 1,552 images were collected, with 51% from male and 49% from female embryos. On day 4, spanning HH22 to HH24, 1,220 images were collected, with a similar distribution between males (52%) and females (48%). Both days combined, 2,772 images were captured, with 51% from male embryos and 49% from female embryos. Readers interested in viewing the LSCI images are invited to see Supporting S2 and S3 Figs, which display sixteen LSCI images randomly selected from day 3 and day 4 datasets.

**Table 1:** Statistical summary of the LSCI images dataset with female and male ratios. A total of 2,773 images were collected from 1,251 eggs.

Incubation Day	Sex	No. of Eggs	No. of Images	Total No. of Images
Day 3 (HH17-HH20)	Male	598 (52%)	794 (51%)	1,553
	Female	543 (48%)	759 (49%)	
Day 4 (HH21-HH25)	Male	479 (52%)	631 (52%)	1,220
	Female	440 (48%)	589 (48%)	
Days 3 & 4	Male	652 (48%)	1,425 (51%)	2,773
	Female	599 (52%)	1,348 (49%)	

The number of images collected per egg was not uniform due to the limited time windows available for imaging, as the eggs can only be imaged during specific time windows. In addition, some images had to be rejected due to poor imaging conditions, such as bad embryo positioning, embryonic body movements, or artifact noise. Ideally, each egg would be represented by a single image per classification stage to avoid potential sampling bias; however, in preliminary experiments, using only one image per egg yielded low classification accuracy (consistently below 60%). To enable a fair comparison (comparable dataset size) with the prior work in Ref. [16] (which also used multiple images per egg), multiple images per egg was used. The cross-validation split is performed on egg-wise basis to prevent data leakage.

For the DNN analysis, we utilized transfer learning with models pretrained on ImageNet, a large-scale dataset containing over 14 million labeled images across thousands of diverse categories [49]. Transfer learning from such large-scale datasets typically provides models with robustness against minor class imbalances in downstream tasks [50,51]. For validation, one image per egg was selected to ensure balanced representation across samples and to prevent any bias that could arise from over-representing individual embryos. This strategy prevents potential bias from over-representation of individual embryos, ensures statistical robustness, and allows for a fair evaluation of the model’s generalizability.

### Data preparation and preprocessing

**Fig 3:**
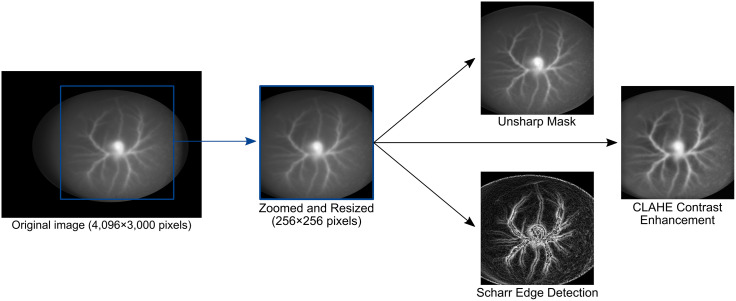
Chicken embryo dataset pre-processing pipeline. Each LSCI image undergoes the same pre-processing pipeline before being input into the algorithm.

The approach used for preprocessing the LSCI images is shown in Fig 3. Monochrome grayscale images were used as input for the DNNs, on the assumption the vascular structure could serve as the primary indicator of sex. First, a center crop was applied to the original image, reducing its dimensions from 4,096 × 3,000 pixels to 3000 × 3000 pixels. To enhance vascular structure, a sliding window approach was used to identify the 30 × 30 pixels region with the highest blood flow index (BFI), derived from speckle contrast. Given that the embryonic heart exhibits the highest perfusion in LSCI images [21, 22], this region was used as a proxy for heart localization. Based on this localization, a 2,300 × 2,300 pixels square region was cropped and centered on the detected location, providing a standardized and biologically relevant region of interest across all samples.

To ensure compatibility with pretrained DNNs, all images were resized to 256 × 256 pixels. Additionally, various image enhancement techniques were applied to optimize feature extraction. Unsharp masking [52,53] was used for image sharpening to enhance image details by subtracting a blurred version of the image from the original. The Scharr operator [54] was employed for edge detection to emphasize gradient edges and features. Finally, Contrast Limited Adaptive Histogram Equalization (CLAHE) [55,56] was applied to enhance contrast while preventing over-amplification of noise.

The complete dataset from days 3 and 4 (Table 1) was split into training and validation sets with an 80/20 split ratio. For the validation set, only one image per egg was used.

### Choice of the deep neural networks (DNNs)

For this study, established deep neural network (DNN) architectures were used. For the specific task of classifying male versus female egg embryo LSCI images, the DNN models were tuned with applied transfer learning and fine-tuning. Four DNN models were trained and tested for final selection: ResNetBiT (Big Transfer ResNet) [57], Xception41 [58], DenseNet121 [59], and InceptionV3 [60]. This preliminary assessment was done using a train/test split instead of cross-validation. Model performance was evaluated based on accuracy, area under the receiver operating characteristic curve (AUC), and loss. The AUC, a standard metric for assessing a model’s ability to distinguish between classes (here, female vs. male), ranges from 0 to 1, with higher values indicating better balance between sensitivity (true positive rates for females) and specificity (true negative rates for males). Accuracy measures the proportion of correctly classified samples, while loss quantifies the error between the predicted and actual labels, with lower values indicating better model performance.

When training accuracy and AUC are high on training data but significantly lower on validation data, and when training loss decreases while the loss stagnates or increases, it indicates overfitting [61]—where the model memorizes training data but fails to generalize to unseen data. The binary cross-entropy (BCE) loss function penalizes incorrect predictions more heavily than it rewards correct ones, making it an effective tool for identifying overfitting [62,63]. Therefore, BCE loss was applied in all the experiments presented in this paper. The BCE loss L  for a set of n predictions $({\hat{y}}_{\text{1}},\;{\hat{y}}_{\text{2}},\;...\;,\;{\hat{y}}_{n}),\;$ given true labels $\left(y_{\text{1}},\;y_{\text{2}},\;...\;,\;y_{n}\right)$ is as follows:


$$\begin{array}{c} L=-\frac{\text{1}}{n}\sum_{i=\text{1}}^{n}{[\;y}_{i}\cdot log({\hat{y}}_{i})+(\text{1}-y_{i})\cdot log(\text{1}-{\hat{y}}_{i})]. \end{array}$$ (2)

After training and validating the four DNNs for 25 epochs, the accuracy, AUC, and loss for each model were as follows: ResNetBiT (accuracy: 51%, AUC: 0.51, loss: 0.72), Xception41 (accuracy: 54%, AUC: 0.54, loss: 0.80), DenseNet121 (accuracy: 53%, AUC: 0.54, loss: 0.85), and InceptionV3 (accuracy: 55%, AUC: 0.57, loss: 0.76). Although ResNetBiT performed slightly worse in terms of accuracy and AUC, it exhibited the least overfitting with lowest loss and with training and validation curves showing similar trends (see Supporting Information S6 Fig). This observation suggests that ResNetBiT may have greater capacity to improve if trained for additional epochs, motivating our focus on optimizing ResNetBiT performance.

In addition to ResNetBiT, a YOLO-based model was tested, as Ref. [16] reported its performance in sex identification of chicken embryos using blood vessel images obtained from egg candling. In that study, a slightly modified YOLOv7 model incorporating a convolutional block attention module (CBAM) achieved an accuracy of 85% or higher on incubation days 3 and 4, with at least 87% precision, 82% recall, and 85% average precision. Reference [16] also compared four models—Faster R-CNN, SSD, YOLOv5, and their modified YOLOv7—for sex identification from blood vessel images on day 4 of incubation. Reference [16] used a dataset comprised of 5,940 images from 2,844 eggs, with an approximate 70/30 training-to-testing split. The results indicated that YOLO models were more effective at identifying blood vessel regions and differentiating sex, with YOLOv5 and modified YOLOv7 exhibiting less than 3% deviation in precision and recall. Consequently, both models (YOLOv5 and YOLOv7) are expected to achieve an accuracy of at least 85%. Since we did not have access to the modified YOLOv7 model from Ref. [16], we instead used the established YOLOv5s-cls model [64] as a comparative baseline for evaluating our results against those reported in the study of Ref. [16].

### Approaches: ResNetBiT and YOLOv5s-cls

To classify the sex of chicken embryos, two established backbone DNN architectures were used: ResNetBiT [57] and YOLOv5s-cls [64], both adapted for binary classification. ResNetBiT is a high-capacity convolutional neural network designed for robust feature extraction and transfer learning. Pretrained on the ImageNet-21k dataset, it is well-suited for transfer learning on small datasets. ResNetBiT was initialized with pretrained weights and fine-tuned it on our dataset. Similarly, YOLOv5s-cls was adapted using its classification variant, rather than its object detection head. This distinction is important, as the model was not used with bounding-box supervision but instead configured as a standard image-level classifier. This approach allowed to explore whether a detection-based model, originally designed for localization tasks, could better suit this problem. The YOLOv5s-cls model was on 640 x 640 pixels images to match the default image size for the model.

**Fig 4:**
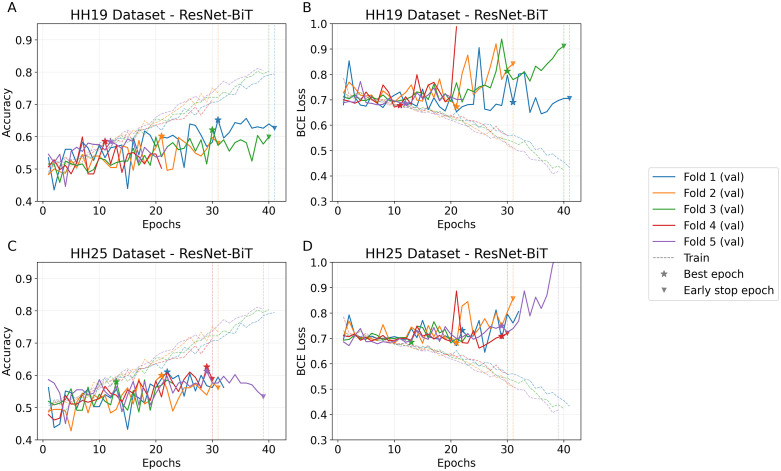
Sex identification results on LSCI images using the ResNetBiT model. **(A)** Accuracy and **(B)** BCE loss as function of the number of epochs for each fold of the training (dotted line) and validation (plain line) datasets on day 3 (HH19) of incubation. Each fold is represented by a different line color. **(C)** Accuracy and **(D)** BCE loss on day 4 (HH25).

Both models were trained using the Adam optimizer, BCE loss function, and cosine annealing learning rate scheduler to ensure stable convergence. To prevent overfitting, several regularization strategies were employed. For ResNetBiT, all backbone layers except the last two stages were frozen, dropout (p = 0.3) and weight decay (1e-4) were added, and a learning rate of 5e-5 was used with a 5-epoch linear warmup followed by cosine annealing. Early stopping was applied from epoch 11 onward using two criteria: validation ACC (patience = 10, minimum improvement = 0.01) and validation loss (patience = 5). For YOLOv5s-cls, backbone layers 0–9 were frozen, dropout (p = 0.1) and applied weight decay (5 × 10 ^−5^) were added with a cosine annealing learning rate scheduler. Early stopping with a patience of 15 epochs was applied after a 20-epoch warmup. Both models used training-only data augmentation including rotations (up to ±180°), translations, scaling, random flips, and brightness/contrast jitter, as well as transfer learning from ImageNet pretrained weights. Five-fold cross-validation with egg-ID-based splits was used to prevent data leakage and ensure reproducibility. We trained on one-channel grayscale images using each of the preprocessing techniques of Fig 3: normal images, unsharp masking, edge detection, and contrast enhancement. And zoomed and resized preprocessing are used in the experiments shown in Fig 4 (resized to 256 × 256) and 5 (resized to 640 × 640).

For both ResNetBit and YOLOv5s-cls models, several regularization strategies were applied exclusively to the training set to improve model generalization and prevent overfitting (see Supporting Information S4 Fig). Training-only data augmentation included random rotations up to 180°, translations up to 5% in any direction, image scaling between 0.85x and 1.15x, random vertical and horizontal flips with probability 0.3, and brightness/contrast/saturation jitter. No augmentations were applied to the validation set. Five-fold cross-validation with egg-ID-based splits were implemented to prevent data leakage and evaluate the model’s performance.

For ResNetBiT, all backbone layers were frozen except the last two stages, dropout (p = 0.3) and weight decay (1e-4) were added, and the learning rate was set to 5e-5 with a 5-epoch linear warmup followed by cosine annealing. Early stopping was applied from epoch 11 onward using two criteria: validation ACC (patience = 10, minimum improvement = 0.01) and validation loss (patience = 5), halting training when either criterion was triggered.

For YOLOv5s-cls, backbone layers 0–9 were frozen, dropout (p = 0.1) was added to the classification head, and weight decay (5 × 10  ^−5^) was applied. Early stopping with a patience of 15 epochs was applied after a 20-epoch warmup. This strategy ensured that training was stopped before training and validation performance significantly diverged (S4 Fig).

## Results

From the days 3 and 4 dataset, a final five-fold cross-validation was conducted with trained ResNetBiT and YOLOv5s-cls models. For each fold, the training set contained approximately 2,200 images (mixed day 3 and day 4 datasets), while the validation set contained around 230 images for day 3 dataset, and 185 images for day 4 dataset. The exact sizes varied slightly between folds because the splits were designed to ensure that images from the same egg were included exclusively in either the training or validation set, and that only one image per egg was included in the validation set.

### ResNetBiT

The five-fold cross-validation results for the ResNetBiT model are presented in Fig 4 and Table 2. Fig 4A and 4C depict the training and validation accuracy curves over the course of epoch for day 3 (HH19) and day 4 (HH25). Fig 4B and 4D show the BCE loss. Early stopping was applied from epoch 11 onward using two criteria: validation ACC (patience = 10, minimum improvement = 0.01) and validation loss (patience = 5).

**Table 2:** Five-fold cross-validated accuracies and p-value results for the ResNetBiT model at day 3 and day 4 of incubation. The significance levels are: p > 0.05 (ns: not significant), p < 0.05 (*), p < 0.01 (**), p < 0.001 (***), and p < 0.0001 (****).

ResNetBiT	Day 3 (HH17-HH20)			Day 4 (HH21-HH25)		
	Accuracy	p-value	α	Accuracy	p-value	α
Fold 1	60%	0.003	**	59%	0.01	*
Fold 2	60%	0.003	**	60%	0.01	*
Fold 3	54%	0.23	ns	57%	0.07	ns
Fold 4	50%	0.96	ns	53%	0.35	ns
Fold 5	54%	0.18	ns	57%	0.07	ns
Average	56%	0.28	ns	57%	0.07	ns

As shown, the validation accuracy curves for day 3 and day 4 datasets fluctuate between 50% and 65%, with average accuracies across five-fold runs of 56% for the day 3 dataset and 57% for the day 4 dataset. To assess whether the classification performance exceeded chance level, p-values were calculated across cross-validation folds (Table 2). For each fold, the p-value was computed using a binomial test under the null hypothesis of random guessing, implemented via a binomial calculator [65]. Specifically, the observed classification accuracy of the deep neural network (DNN) for each fold was compared against the expected accuracy from random classification of male versus female egg embryo LSCI images (i.e., a chance level of 50%). The significance levels α are established as follows: p > 0.05 (ns: not significant), p < 0.05 (*), p < 0.01 (**), p < 0.001 (***), and p < 0.0001 (****).

To assess model interpretability, we applied gradient-weighted class activation mappings (Grad-CAM) to generate heatmaps highlighting regions influencing the ResNetBiT predictions, see Supporting Information S5 Fig. The model’s attention was primarily localized within the egg, often near the embryo vascular region, rather than the external background, suggesting that the model was driven by the blood vessels’ structure rather than the eggshell artefacts or illumination bias. Despite focusing on biologically relevant regions, the model did not capture consistent sex-discriminative features, supporting our conclusion that LSCI vascular pattern images at day 3 (stage HH19) and day 4 (HH25) do not contain sufficient signal for reliable early-stage sex classification.

Table 2 shows the accuracy, p-value, and significance level α for each fold of the ResNetBiT model tested on datasets from day 3 and day 4 of incubation. As the five-fold experiments are performed with the same dataset, their prediction accuracy and associated p-value are not independent of each other. While the accuracy averaged across the five-fold provide some measure of the overall accuracy, a more meaningful approach would be to look at the worst performing p-value across the folds. From Table 2, the worst performing p for ResNetBiT for experiment was 0.96 for day 3 and 0.35 for day 4, significantly higher than any significance level. Using our established significance level, the ResNetBiT did not pass the significance threshold. The average accuracy of 56% (57%) at day 3 (day 4) is noteworthy not enough for practical applications. These findings are further supported by the training and validation BCE loss curves in Fig 4B and 4D. The training loss decreases from 0.7 to below 0.5 over 30 epochs, while the validation loss curves slightly increase from 0.70 to 0.80. This suggests slight overfitting of the model, confirming that it is not suitable for practical application. For a random binary guess, the expected BCE loss is log(2)≈0.693.

### YOLOv5s-cls

**Fig 5:**
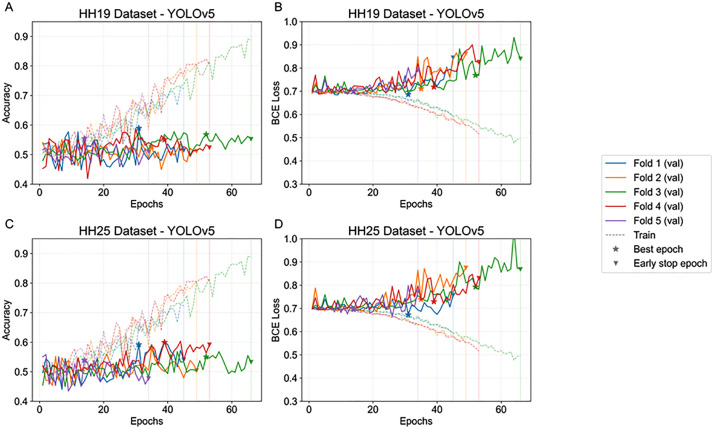
Sex identification results on LSCI images using the YOLOv5s-cls model. **(A)** Accuracy and **(B)** BCE loss as function of the number of epochs for each fold of the training (dotted line) and validation (plain line) datasets on day 3 of incubation. Each fold is represented by a different line color. **(C)** Accuracy and **(D)** BCE loss on day 4.

The five-fold cross-validated results for the YOLOv5s-cls model are presented in Fig 5 and Table 3. Fig 5A and 5C depict the training and validation accuracy curves over the course of epochs for day 3 and day 4. Fig 5B and 5D show the BCE loss. To mitigate overfitting, the following strategies were adopted and described in Approaches: ResNetBiT and YOLOv5s-cls section of the Materials and Methods.

**Table 3:** Five-fold cross-validated accuracies and p-value results for the YOLOv5s-cls model at day 3 and day 4 of incubation. The significance levels are: p > 0.05 (ns: not significant), p < 0.05 (*), p < 0.01 (**), p < 0.001 (***), and p < 0.0001 (****).

YOLOv5	Day 3 (HH17-HH20)			Day 4 (HH21-HH25)		
	Accuracy	p-value	α	Accuracy	p-value	α
Fold 1	58%	0.02	ns	59%	0.016	*
Fold 2	52%	0.36	ns	58%	0.03	*
Fold 3	56%	0.062	ns	55%	0.13	ns
Fold 4	55%	0.10	ns	59%	0.016	*
Fold 5	55%	0.10	ns	53%	0.29	ns
Average	55%	0.13	ns	57%	0.10	ns

As shown, the training accuracy also increases with the number of epochs, surpassing 70% after 30 epochs. The validation accuracy curves for day 3 and day 4 datasets fluctuate between 50% and 60%, with average accuracies across five-fold runs of 55% for the day 3 dataset and 57% for the day 4 dataset. The worst performing p-values for YOLOv5s-cls were 0.36 for day 3 and 0.29 for day 4. Using our established significance level, neither passed the significance threshold. These findings are further supported by the training and validation BCE loss curves in Figs 5B and 5D. The training loss decreased from 0.7 to around 0.6 over 40 epochs, while the validation loss curves slightly increased from 0.70 to 0.80, suggesting overfitting, even though multiple strategies to prevent overfitting were used. Table 3 shows the accuracy, p-value, and significance level α for each fold of the YOLOV5s-cls model tested on datasets from day 3 (HH19) and day 4 (HH25) of incubation.

To summarize, despite early-stopping and other strategies preventing overfitting such as backbone freezing, dropout, weight decay, cosine annealing, and extensive data augmentation (see Materials and methods), as shown in Fig 5, the validation accuracy did not improve meaningfully across training epochs, remaining close to chance level. While the validation performance remained near chance, the training accuracy increased, indicating that while overfitting was somehow controlled, reliable generalization was not achieved. This suggests that LSCI blood vessels images lack sufficiently robust sex-discriminative features for models such as YOLOv5 to learn from, and therefore the training process did not yield a valid model for hypothesis testing purposes.

### Independent tests with ResNet50 and YOLOVv8-nano

Additionally, the classification performance was independently validated (by a researcher not involved in the ResNetBiT or YOLOv5s-cls study) using a modified ResNet50 model and a YOLOv8-nano classification model. The models processed egg images from both day 3 (HH19) and day 4 (HH25) developmental stages, supporting training on individual stages as well as a mixed-stage dataset. For the ResNet50 model, the architecture was adapted for single-channel grayscale input by modifying the first convolutional layer (Conv2d, 1◊64 channels, 7 × 7 kernel, stride 2, padding 3) and adjusting the final fully connected layer 2048 → 2 outputs. Models were trained both with ImageNet-pretrained weights and from random initialization. Input images were resized to 224 × 224 pixels and subjected to data augmentation (random resized cropping and horizontal flipping), followed by normalization (μ = 0.5, σ = 0.5). Optimization was performed using the Adam optimizer (learning rate of 10^−3^) with cross-entropy loss for 25 epochs with a batch size of 32. For the YOLOv8 model, we used the nano classification variant (yolov8n-cls), rather than the object detection architecture, to perform image-level binary classification. The model was trained on 512 × 512 pixel images with a batch size of 16 for 50 epochs.

Both architectures were evaluated across three dataset configurations: HH19 only (day 3; n = 1,287 images), HH25 only (day 4; n = 1,048 images), and a mixed dataset combining both stages (n = 2,335 images). For both architectures, the dataset was split into training and validation sets using an 80/20 stratified split based on sex labels, across HH19, HH25, and mixed-stage conditions. All experiments were tracked using weights and biases to ensure reproducibility and consistent performance monitoring. To account for split variability, all experiments were repeated across five independent random seeds and results are reported as mean ± standard deviation of the best validation accuracy (Supporting S1 Table). ResNet50 performed with accuracies of 52% on mixed datasets, 51% on day 3 dataset, and 52% on day 4 dataset. YOLOv8n-cls performed with accuracies of 57% on mixed datasets, 57% on day 3 dataset, and 59% on day 4 dataset. All accuracies remained below the 60% level. Therefore, both models achieved validation performance close to chance level across configurations, consistent with the results obtained from other architectures. These findings further support the conclusion that LSCI vascular pattern alone does not provide a sufficiently robust discriminative signal for reliable early sex identification.

### Comparison with Previous Studies

Our study was unable to accurately distinguish the sex of chick embryos from LSCI images at days 3 and 4. Two DNN models were trained: ResNetBiT and YOLOv5, neither achieved practically useful accuracy (80% or better). In fact, none achieved statistical significance.

These results are puzzling given that Ref. [16] reported over 85% accuracy with a low loss using YOLO models on chick embryo blood vessel images from day 3 or 4 of incubation. Additionally, Ref. [16] compared YOLOv5 and their modified YOLOv7 for sex identification from blood vessel images on day 4 of incubation, using a dataset of 5,940 images from 2,844 eggs with a 70/30 train-test split. Their results demonstrated that YOLO models effectively identified blood vessel regions and differentiated sex, with YOLOv5 and modified YOLOv7 showing less than a 3% deviation in precision and recall. Based on these findings, both models were expected to achieve at least 85% accuracy, making our lower performance particularly unexpected. We could not pinpoint a major factor explaining the disparity between results. Their training set was almost twice as large, but we do not expect this alone would account for such a significant deviation in accuracy. Despite multiple attempts, we were unable to obtain access to the dataset and/or code used in Ref. [16].

## Discussions

In this study, we evaluated the feasibility of using deep neural networks (DNNs) to classify the sex of chicken embryos from laser speckle contrast imaging (LSCI)-derived vascular images. Despite employing multiple established DNN architectures with transfer learning and fine-tuning, the overall classification performance remained modest, with the best-performing model achieving an accuracy below 60%. These results indicate that, under the current experimental and analytical conditions, sex classification at early-stages from vascular patterns alone is marginally above chance and not reliable. Importantly, these findings are consistent with the central conclusion of this work: sex-specific differences in vascular structure at the investigated developmental stages (day 3 and day 4) are either too subtle to be captured by LSCI or not present at all.

A key motivation for this paper was to contrast our findings with prior work (Ref. [16]), which reported classification accuracies exceeding 85% using similar vascular images (egg candling). Despite using a comparable methodological framework, we were unable to reproduce such high performance. The reasons for this discrepancy remain unclear. Notably, the dataset used in that study is not publicly available, and attempts to obtain additional details were unsuccessful, preventing direct comparison and reproducibility assessment.

Note that the choice to downsample images from 4096 × 3000–256 × 256 pixels was driven by compatibility with standard pre-trained DNN architectures and computational constraints. While this reduction in resolution may lead to loss of fine vascular details, experiments with higher input resolution (512 × 512) did not improve performance. Future work may explore alternative strategies, such as multi-scale approaches, higher-resolution DNN architectures, or image pre-processing techniques to better preserve and exploit the subtle vascular patterns. To promote transparency and enable further progress in this matter, we have made our dataset of original LSCI images (of size 4096 × 3000 pixels) publicly available, see Data availability section.

LSCI is particularly well suited for capturing hemodynamic and perfusion-related information (blood flow time trace).

Exploration of temporal features, including embryonic heart rate and blood flow dynamics, did not reveal reliable sex-specific differences at (day 3–4) developmental stages (see Supporting Information). At these stages, the embryonic heart remains immature and has not yet developed into a fully structured four-chamber organ with a stable and regular beating pattern. Consequently, heart rate measurements exhibited inter-embryo variability, driven by differences in developmental stage rather than sex. In addition, heart rate measurements are sensitive to temperature fluctuations, where variations as small as ±1 °C were found to affect the measurements. Analysis at a later stage (day 6), where cardiac development is more advanced, was conducted on 39 eggs (20 female and 19 male). The temperature of the eggs was always above 37 °C. The results, shown in Supporting S7 Fig, did not reveal clear sex-specific differences in heart rate between female and male, however, a larger dataset would be required to draw more definitive conclusions

Future studies could explore alternative approaches, such as multiple-instance learning to assess spatial and temporal variations in blood vessel patterns, feature extraction from LSCI images, blood flow dynamics and heart rate analysis, which would require precise temperature control during image acquisition for accurate measurements. These refinements may offer new insights into the potential for early-stage sex differentiation in chick embryos using LSCI imaging.

## Conclusion

Using laser speckle contrast imaging (LSCI), images of chick embryo blood vessels at days 3 and 4 of incubation were recorded to explore the feasibility of sex differentiation using deep neural network (DNN) models. Our final dataset consisted of images from 1,251 chick embryos, providing a substantial sample size for a pilot DNN testing. Two DNN models were trained: ResNetBiT and YOLOv5s-cls, both achieved not satisfactory accuracy and relatively high BCE loss, with YOLOv5s-cls performing worse than ResNetBiT. A per-egg evaluation approach did not achieve high accuracy or statistically robust results, indicating that, under the current experimental conditions and modeling approaches, we did not observe sufficiently strong predictive performance to support reliable sex classification at early incubation stages. We emphasize that this is not a definitive conclusion. Further refinements and optimizations of the models or alternative imaging approaches could potentially improve performance.

Importantly, these negative results are unlikely to stem primarily from model limitations. Instead, they are more plausibly explained by fundamental biological constraints at this early developmental stage. Previous literature on genetic expression related to sex differentiation in embryos indicates that most genes influencing sex determination begin to express around day 4.5 of incubation in male embryos and day 6.5 in female embryos [23]. From day 6.5 onward, gradual morphological changes in gonadal tissue become observable, marking the onset of sex organ development. At days 3–4, when our imaging was performed, sex-specific physiological or vascular signatures are therefore expected to be minimal or absent, limiting the availability of discriminative features in blood vessel images.

Additionally, the chorioallantoic membrane (CAM)—a highly vascularized extra-embryonic membrane—plays a crucial role in embryonic development, supporting vital functions such as gas exchange, calcium absorption, and waste removal. Since the CAM begins to form around day 5 of incubation (stage HH25) and is closely linked to the embryo’s genetic identity and overall development, we hypothesize that it may also be involved in sex differentiation. Given that the CAM is rich in active blood vessels and continues developing until days 12–14, this could explain why sex identification through blood vessel imaging may become more viable at later incubation stages.

While we hypothesize that sex differentiation may be detectable from day 6 onward, our current LSCI system is limited to imaging up to day 5.5 due to increased embryonic motion, which degrades speckle contrast measurements during multi-frame acquisition (total acquisition time of 5 s for 100 frames at 20 FPS, 10 ms exposure). To address this limitation, we plan to upgrade to a higher-speed camera (90 FPS), reducing the inter-frame interval to 11 ms and the total acquisition time to approximately 1.1 s, minimizing motion-induced artifacts. This improvement is expected to enable reliable LSCI imaging at day 6 and beyond.

## Supporting information

S1 Fig Experimental arrangement of our LSCI system for imaging the active blood vessels of a chick embryo non-invasively. The whole system was encased in a black box to avoid stray light. See Ref. (16) for a detailed experimental arrangement.
(PNG)

S2 Fig Example of 16 LSCI images before pre-processing. The images were randomly selected from day 3 dataset.
(PNG)

S3 Fig Example of 16 LSCI images before pre-processing. The images were randomly selected from day 4 dataset.
(PNG)

S4 Fig Training and validation loss curves for different levels of rotation data augmentation (±10°, ± 30°, ± 60°, ± 120°, and ±180°). Increasing rotation strength reduced the divergence between training and validation loss, consistent with decreased overfitting. The most significant effect was observed at ±180°.
(PNG)

S5 Fig Gradient-weighted class activation mappings (Grad-CAM) heatmap visualizations of the early-stopped ResNetBiT model trained on LSCI egg images, highlighting image regions contributing to model predictions. (PNG)

S6 Fig Training and validation curves for ResNetBiT, Xception41, DenseNet121, and InceptionV3 over 25 epochs on the full dataset (HH19 + HH25). Although ResNetBiT performs the worst in terms of accuracy, it exhibits the least overfitting, with training and validation losses relatively aligned, suggesting higher potential for learning with extended training.
(PNG)

S7 Fig (Left) Statistical distribution of heart rate for male and female chicken embryos at day 6 of incubation. Each dot in the graph represents a chick embryo. (Right) Summary table showing mean, median, and quartiles of heart rate for each sex. As shown, there is no clear statistical difference between female and male in heart rate, even though the male distribution appears more dispersed.
(PNG)

S1 Table Binary sex classification accuracy (%) for ResNet50 and YOLOv8 across developmental stages, reported as mean ± std over 5 train/validation splits. (PNG)
