# Supplementary figures and images for: Detection of non-invasive sexing of early chick embryos in intact eggs using laser speckle contrast imaging and deep neural networks
Source: PLoS One. 2026 Jun 26;21(6):e0323847. doi: 10.1371/journal.pone.0323847 (PMC13308874; doi:10.1371/journal.pone.0323847)

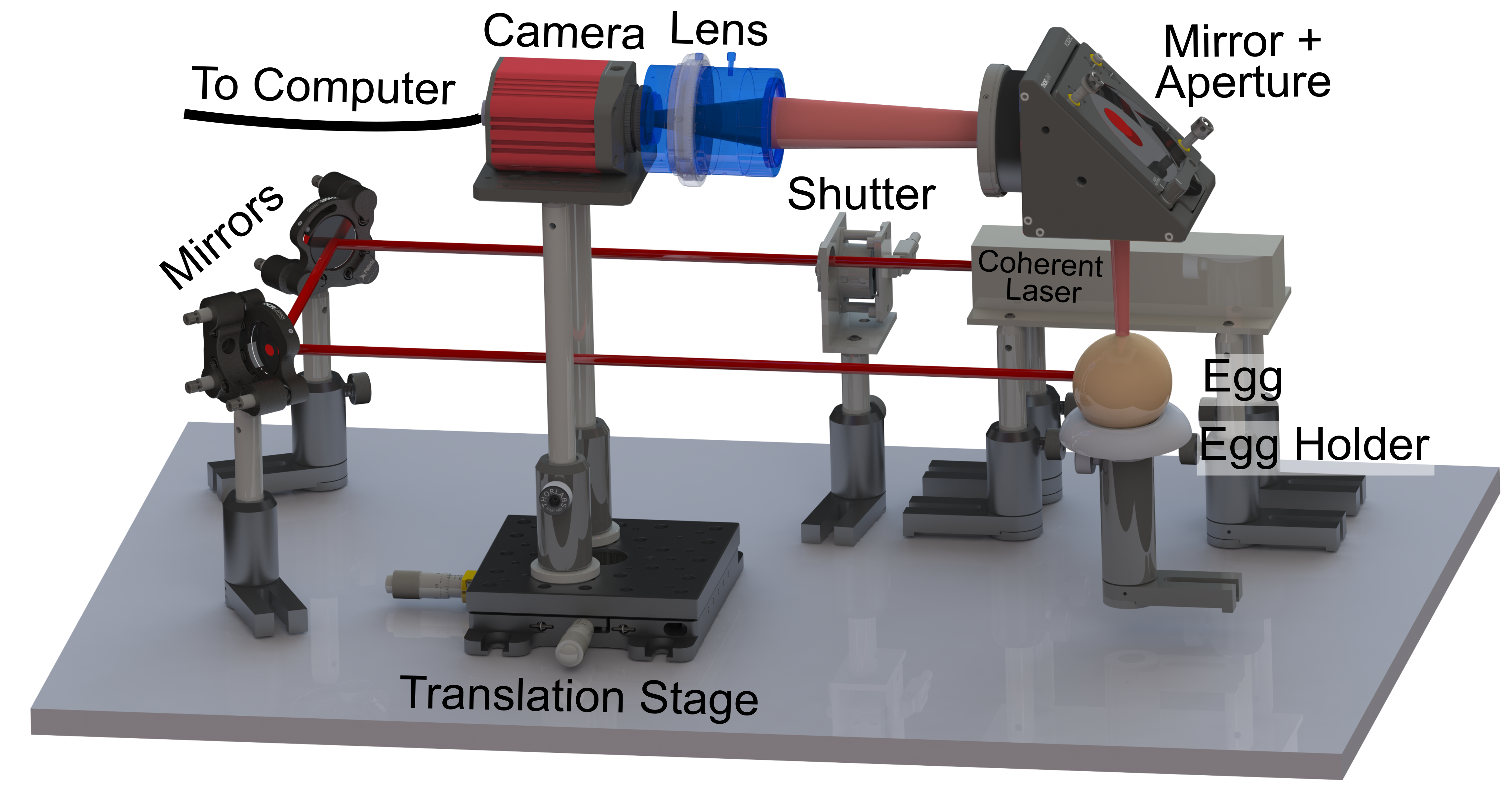

Supplement: S1 Fig — The whole system was encased in a black box to avoid stray light. See Ref. (16) for a detailed experimental arrangement. (PNG) [file pone.0323847.s001.png]

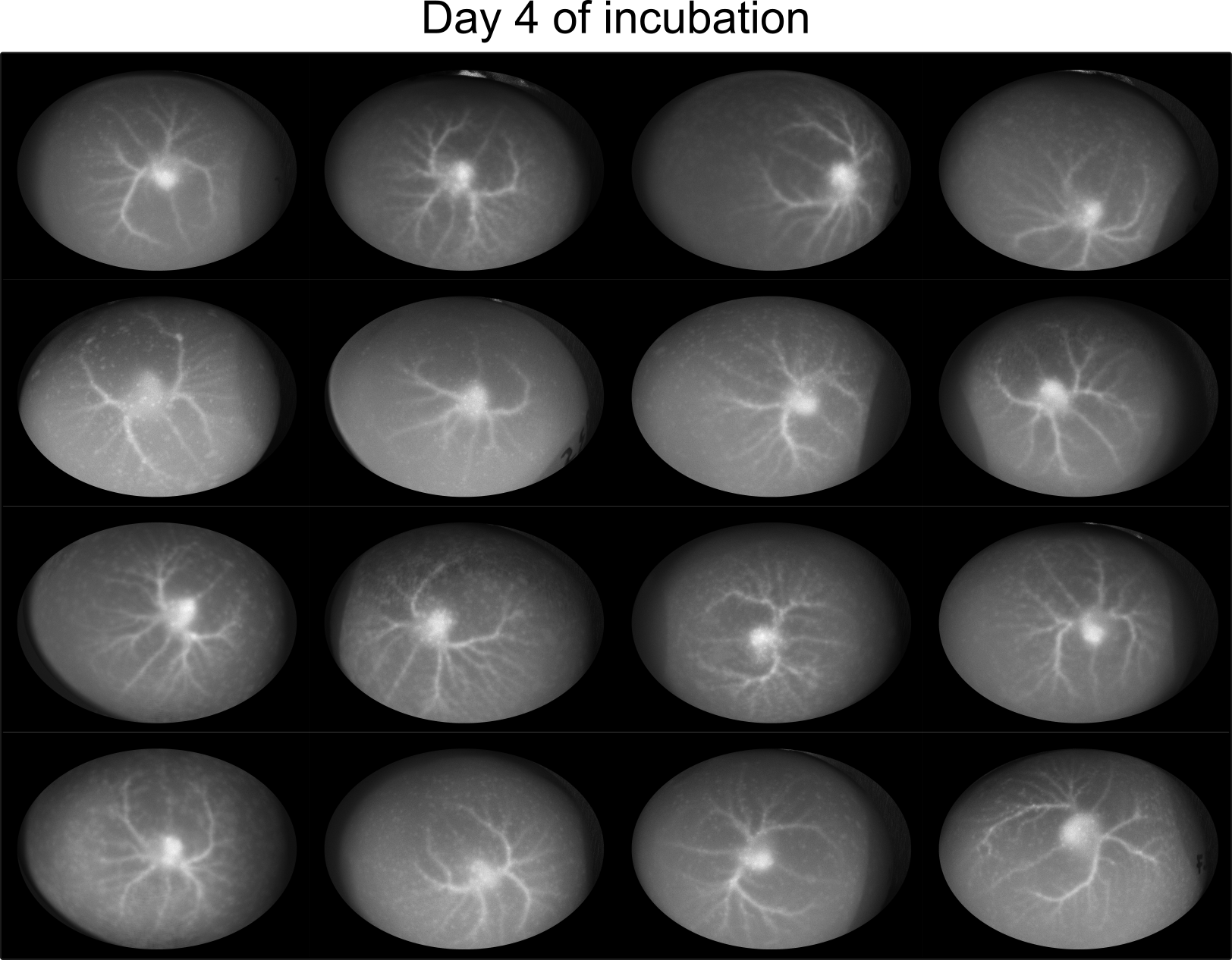

Supplement: S2 Fig — The images were randomly selected from day 3 dataset. (PNG) [file pone.0323847.s002.png]

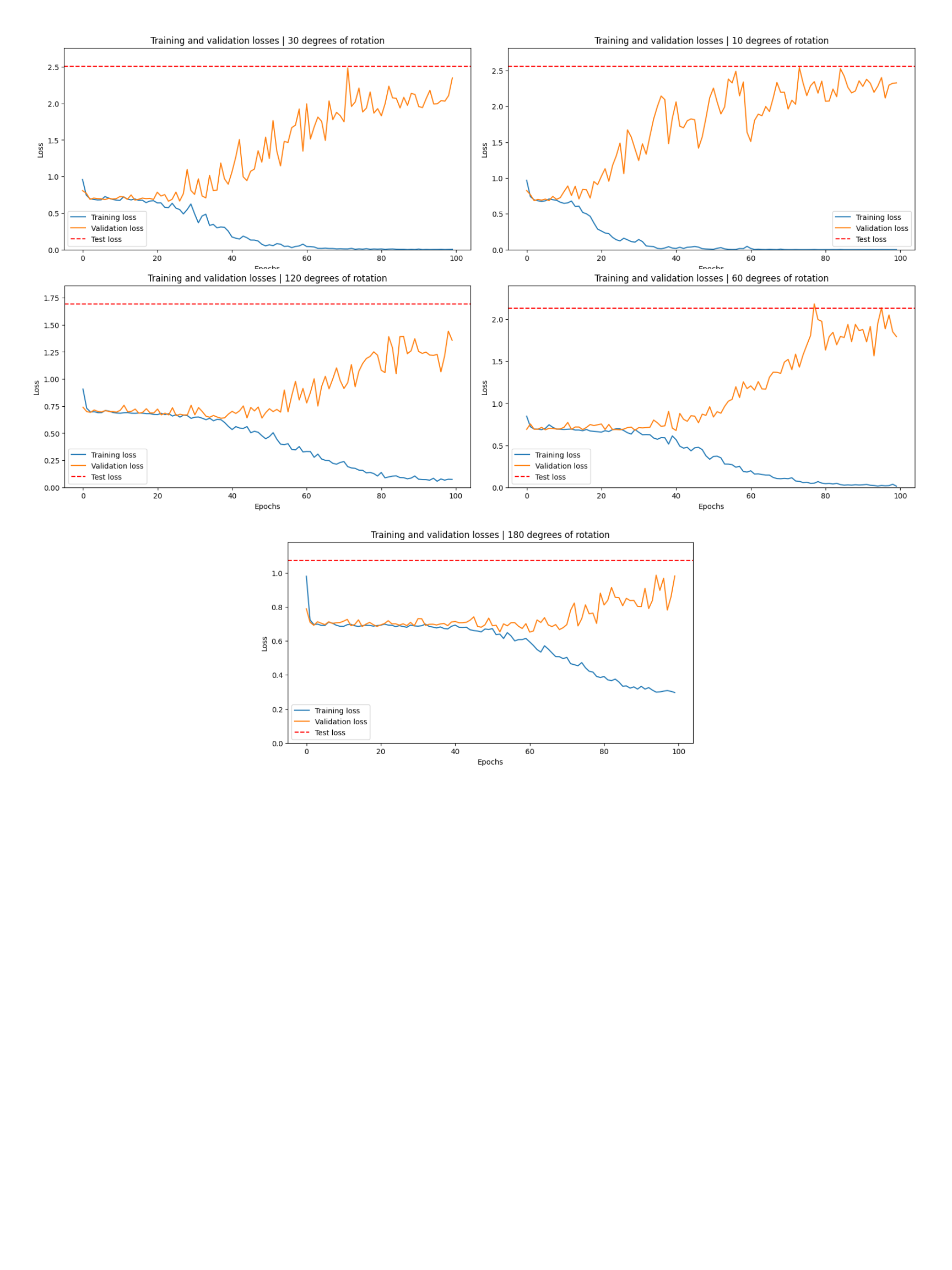

Supplement: S4 Fig — Increasing rotation strength reduced the divergence between training and validation loss, consistent with decreased overfitting. The most significant effect was observed at ±180°. (PNG) [file pone.0323847.s004.png]

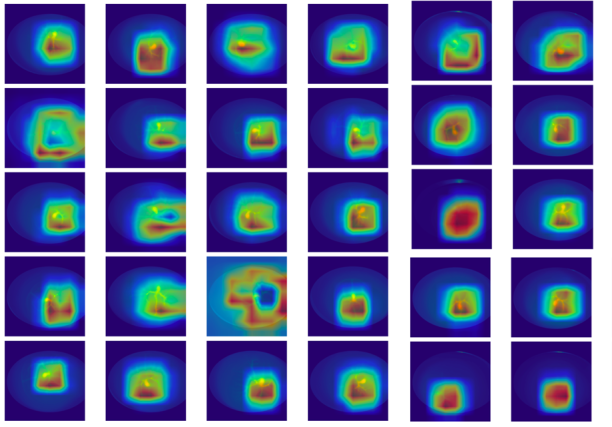

Supplement: S5 Fig — (PNG) [file pone.0323847.s005.png]

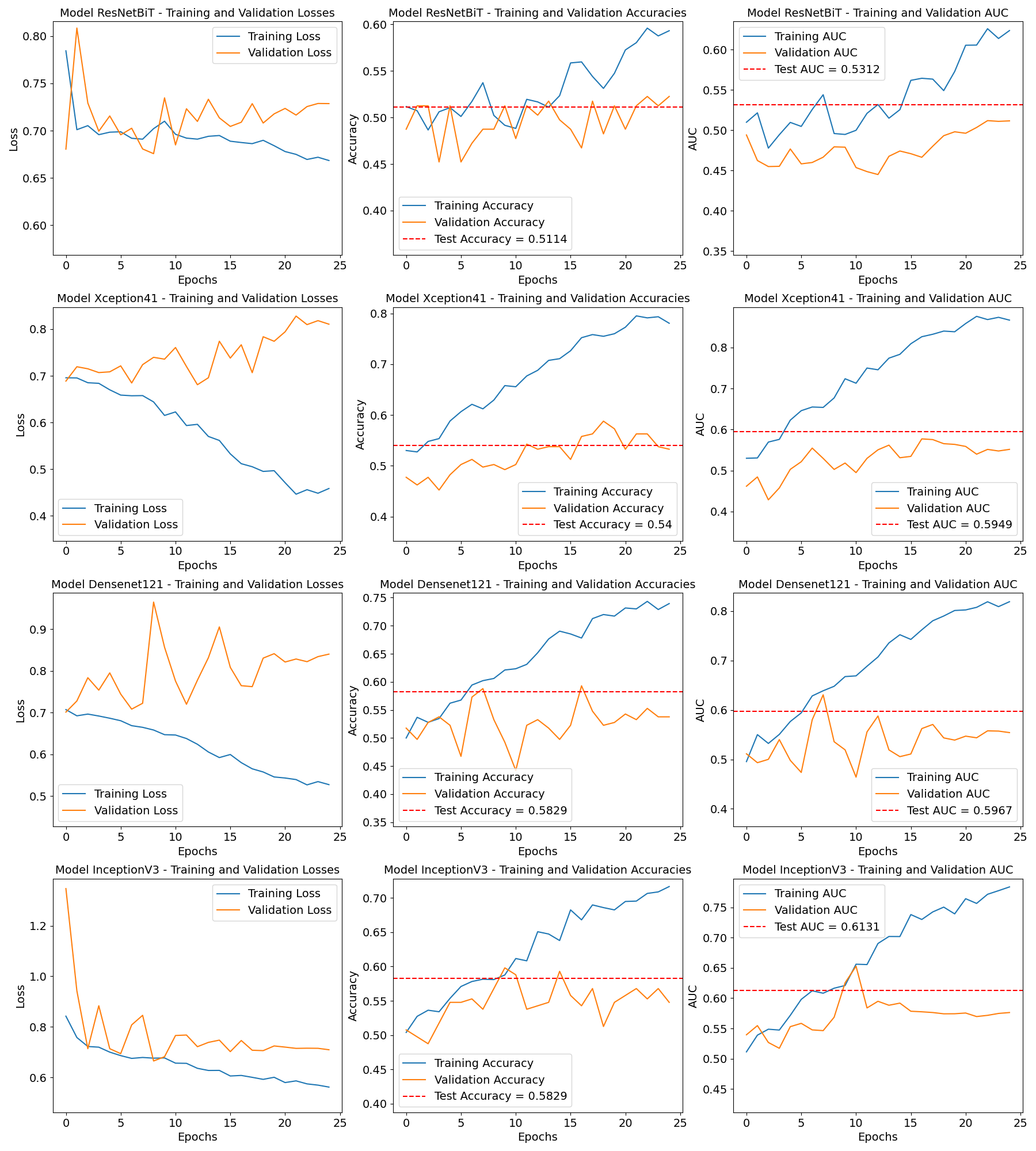

Supplement: S6 Fig — Although ResNetBiT performs the worst in terms of accuracy, it exhibits the least overfitting, with training and validation losses relatively aligned, suggesting higher potential for learning with extended training. (PNG) [file pone.0323847.s006.png]

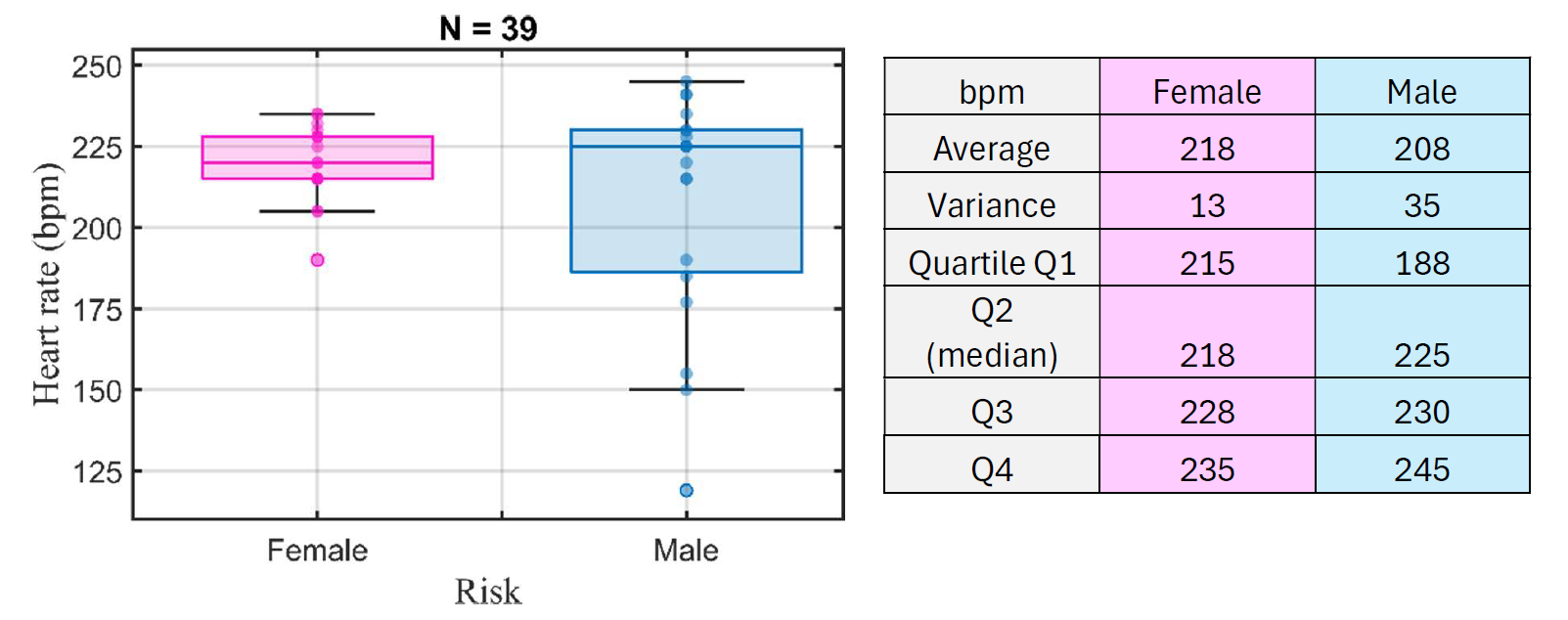

Supplement: S7 Fig — Each dot in the graph represents a chick embryo. (Right) Summary table showing mean, median, and quartiles of heart rate for each sex. As shown, there is no clear statistical difference between female and male in heart rate, even though the male distribution appears more dispersed. (PNG) [file pone.0323847.s007.png]

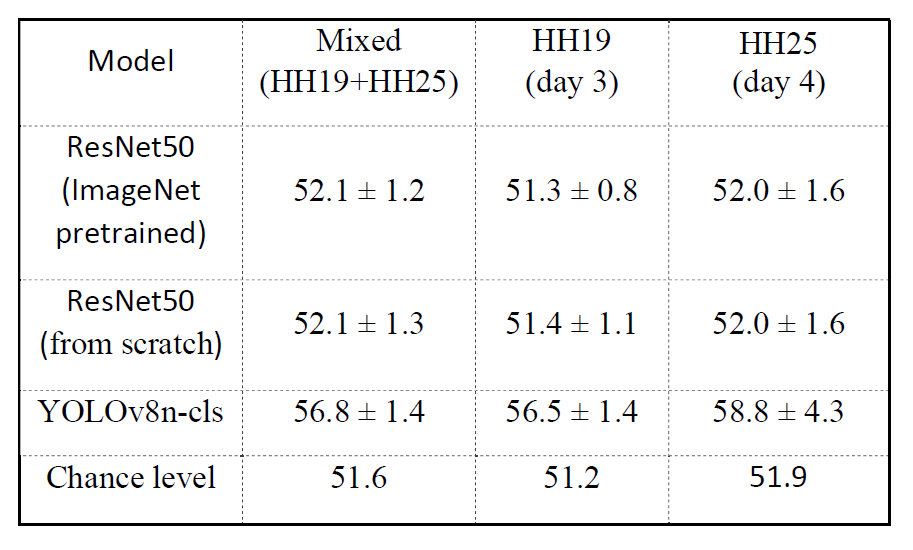

Supplement: S1 Table — (PNG) [file pone.0323847.s008.png]
