# Supplementary material for: Detection of non-invasive sexing of early chick embryos in intact eggs using laser speckle contrast imaging and deep neural networks
Source: PLoS One. 2026 Jun 26;21(6):e0323847. doi: 10.1371/journal.pone.0323847 (PMC13308874; doi:10.1371/journal.pone.0323847)
Supplement: Attachment — Submitted filename: Response to Reviewers.pdf [file pone.0323847.s009.pdf]

Manuscript ID: PONE-D-25-20406

Title: Detection of non-invasive sexing of early chick embryos in intact eggs using laser speckle contrast imaging and deep neural networks

Authors: S. Mahler et al; California Institute of Technology

Subject: Revised manuscript + response letter to reviewer's comments

Dear Khan Bahadar Khan,

We have received your decision on March-11, 2026 to give us an opportunity to revise our manuscript before final decision. Below, we address the reviewers' comments, editorial comments and instructions, together with our changes made to the manuscript. We note that substantial revisions have been made to the manuscript, and we believe the resubmitted version addresses the comments and suggestions raised by the four reviewers.

We appreciate your consideration and look forward to receiving your final decision soon. We also attach to this resubmission a marked-up copy of the revised manuscript (labeled "Revised Manuscript with Track Changes") and an unmarked version without tracked changes (labeled "Manuscript").

We do not wish to make any change to the financial disclosure.

Editorial Comments:

"1. Please ensure that your manuscript meets PLOS ONE's style requirements, including those for file naming. The PLOS ONE style templates can be found at

https://journals.plos.org/plosone/s/file?id=wjVg/PLOSOne_formatting_sample_main_body.pdf and https://journals.plos.org/plosone/s/file?id=ba62/PLOSOne_formatting_sample_title_authors_affiliations.pdf."

We have revised the manuscript to comply with the formatting and style requirements of PLOS ONE. Specifically, we reformatted the manuscript using the PLOS ONE Word template available on ResearchGate and updated the reference list to match the PLOS ONE citation style. We also verified that the manuscript complies with the formatting guidelines outlined in the two sample PDF documents provided above.

"2. Please note that PLOS ONE has specific guidelines on code sharing for submissions in which author-generated code underpins the findings in the manuscript. In these cases, we expect all author-generated code to be made available without restrictions upon publication of the work.
Please review our guidelines at https://journals.plos.org/plosone/s/materials-and-software-sharing#loc-sharing-code and ensure that your code is shared in a way that follows best practice and facilitates reproducibility and reuse."

We have created a repository including the code and dataset used for the findings of this manuscript. The repository was uploaded on Zonedo public repository, one of the recommended repositories by PLOS One. The repository can be found at this URL:
https://zenodo.org/records/19004322?token=eyJhbGciOiJIUzUxMiJ9.eyJpZCI6IjYwZTdiMGVjLTMyOWEtNDY5MS05YTVjLWQ0OTU1ZDQzNmY0OCIsImRhdGEiOnt9LCJyYW5kb20iO

iIzZjQwNzlhZDY5NTJjNTdiNzY4NTljNGQ5Y2M1ZTBkZCJ9.tIFalcb3V9aCwCLno5uCitBhZFBinbXC6pnOZzHFka6EPekYvE56muUQfPyiaNRORSbfIW3Q3CG23RxTZkc0nQ

“3. We note that the grant information you provided in the ‘Funding Information’ and ‘Financial Disclosure’ sections do not match.

When you resubmit, please ensure that you provide the correct grant numbers for the awards you received for your study in the ‘Funding Information’ section.”

We have added the grant information (IST Carver Mead Endowment (25550038)) and its associated details (per the guidelines) in the ‘Financial Disclosure’ section only.

“4. Thank you for stating the following financial disclosure: “IST Carver Mead Endowment (25550038)”

Please state what role the funders took in the study. If the funders had no role, please state: "The funders had no role in study design, data collection and analysis, decision to publish, or preparation of the manuscript." If this statement is not correct you must amend it as needed.

Please include this amended Role of Funder statement in your cover letter; we will change the online submission form on your behalf.”

We confirm that the funder (IST Carver Mead Endowment) had no role in study design data collection and analysis, decision to publish or preparation of the manuscript. Per suggestion of the editorial team, we have added the following sentence in the financial disclosure section:

"The funders had no role in study design, data collection and analysis, decision to publish, or preparation of the manuscript."

“5. Please note that funding information should not appear in the Acknowledgments section or other areas of your manuscript. We will only publish funding information present in the Funding Statement section of the online submission form. Please remove any funding-related text from the manuscript.”

We removed any funding information from the Acknowledgments section and related text from the manuscript. The funding information is now provided only in the Funding Statement section of the online submission form.

“6. We note that you have indicated that there are restrictions to data sharing for this study. PLOS only allows data to be available upon request if there are legal or ethical restrictions on sharing data publicly. For more information on unacceptable data access restrictions, please see http://journals.plos.org/plosone/s/data-availability#loc-unacceptable-data-access-restrictions.

Before we proceed with your manuscript, please address the following prompts:

a) If there are ethical or legal restrictions on sharing a de-identified data set, please explain them in detail (e.g., data contain potentially identifying or sensitive patient information, data are owned by a third-party organization, etc.) and who has imposed them (e.g., a Research Ethics Committee or Institutional Review Board, etc.). Please also provide contact information for a data access committee, ethics committee, or other institutional body to which data requests may be sent.

b) If there are no restrictions, please upload the minimal anonymized data set necessary to replicate your study findings to a stable, public repository and provide us with the relevant URLs, DOIs, or accession numbers. For a list of recommended repositories, please see https://journals.plos.org/plosone/s/recommended-repositories. You also have the option of uploading the data as Supporting Information files, but we would recommend depositing data directly to a data repository if possible.

We will update your Data Availability statement on your behalf to reflect the information you provide."

We have created a public repository containing the dataset and code used to generate the findings reported in this manuscript. The repository has been deposited in Zenodo, which is one of the repositories recommended by PLOS ONE for data sharing. The repository can be accessed at the following link:

https://zenodo.org/records/19004322?token=eyJhbGciOiJIUzUxMiJ9.eyJpZCI6IjYwZTdiMGVjLTMyOWEtNDY5MS05YTVjLWQ0OTU1ZDQzNmY0OCIsImRhdGEiOnt9LCJyYW5kb20iOiIzZjQwNzlhZDY5NTJjNTdiNzY4NTljNGQ5Y2M1ZTBkZCJ9.tIFalcb3V9aCwCLno5uCitBhZFBinbXC6pnOZzHFka6EPekYvE56muUQfPyiaNRORSbfIW3Q3CG23RxTZkc0nQ

This dataset represents the minimal anonymized data required to replicate the results presented in the study.

"7. In this instance it seems there may be acceptable restrictions in place that prevent the public sharing of your minimal data. However, in line with our goal of ensuring long-term data availability to all interested researchers, PLOS' Data Policy states that authors cannot be the sole named individuals responsible for ensuring data access (http://journals.plos.org/plosone/s/data-availability#loc-acceptable-data-sharing-methods).

Data requests to a non-author institutional point of contact, such as a data access or ethics committee, helps guarantee long term stability and availability of data. Providing interested researchers with a durable point of contact ensures data will be accessible even if an author changes email addresses, institutions, or becomes unavailable to answer requests.

Before we proceed with your manuscript, please also provide non-author contact information (phone/email/hyperlink) for a data access committee, ethics committee, or other institutional body to which data requests may be sent. If no institutional body is available to respond to requests for your minimal data, please consider if there any institutional representatives who did not collaborate in the study, and are not listed as authors on the manuscript, who would be able to hold the data and respond to external requests for data access? If so, please provide their contact information (i.e., email address). Please also provide details on how you will ensure persistent or long-term data storage and availability."

We have created a public repository containing the dataset and code used to generate the findings reported in this manuscript. The repository can be accessed at the following

Dataset URL:

https://zenodo.org/records/19004322?token=eyJhbGciOiJIUzUxMiJ9.eyJpZCI6IjYwZTdiMGVjLTMyOWEtNDY5MS05YTVjLWQ0OTU1ZDQzNmY0OCIsImRhdGEiOnt9LCJyYW5kb20iOiIzZjQwNzlhZDY5NTJjNTdiNzY4NTljNGQ5Y2M1ZTBkZCJ9.tIFalcb3V9aCwCLno5uCitBhZFBinbXC6pnOZzHFka6EPekYvE56muUQfPyiaNRORSbfIW3Q3CG23RxTZkc0nQ

Code URL: https://github.com/anika24/lsci-egg-sex-classification

The non-author contact information for data access or other institutional body to which data requests may be sent is: Anne M. Sullivan, Staff Laboratory Manager, Caltech Electrical Engineering. Office Phone: 626-395-4506 - Email: annes@caltech.edu

"8. We note that Figures 1 and 2 in your submission contain copyrighted images. All PLOS content is published under the Creative Commons Attribution License (CC BY 4.0), which means that the manuscript, images, and Supporting Information files will be freely available online, and any third party is permitted to access, download, copy, distribute, and use these materials in any way, even commercially, with proper attribution. For more information, see our copyright guidelines: http://journals.plos.org/plosone/s/licenses-and-copyright.

We require you to either (1) present written permission from the copyright holder to publish these figures specifically under the CC BY 4.0 license, or (2) remove the figures from your submission:

1) You may seek permission from the original copyright holder of Figures 1 and 2 to publish the content specifically under the CC BY 4.0 license.

We recommend that you contact the original copyright holder with the Content Permission Form (http://journals.plos.org/plosone/s/file?id=7c09/content-permission-form.pdf) and the following text: "I request permission for the open-access journal PLOS ONE to publish XXX under the Creative Commons Attribution License (CCAL) CC BY 4.0 (http://creativecommons.org/licenses/by/4.0/). Please be aware that this license allows unrestricted use and distribution, even commercially, by third parties. Please reply and provide explicit written permission to publish XXX under a CC BY license and complete the attached form."

Please upload the completed Content Permission Form or other proof of granted permissions as an ""Other"" file with your submission.

In the figure caption of the copyrighted figure, please include the following text: "Reprinted from [ref] under a CC BY license, with permission from [name of publisher], original copyright [original copyright year]."

2) If you are unable to obtain permission from the original copyright holder to publish these figures under the CC BY 4.0 license or if the copyright holder's requirements are incompatible with the CC BY 4.0 license, please either i) remove the figure or ii) supply a replacement figure that complies with the CC BY 4.0 license. Please check copyright information on all replacement figures and update the figure caption with source information.

If applicable, please specify in the figure caption text when a figure is similar but not identical to the original image and is therefore for illustrative purposes only."

We consulted with the PLOS ONE editorial office regarding this matter. The potential copyright concern in Figures 1 and 2 was related to the use of images resembling clip art (specifically, the egg images in both figures and the incubator drawing in Figure 2). To eliminate any copyright concerns, we have replaced these elements with original photographs. Specifically, we have taken new photographs of the eggs and substituted the clip-art incubator image with a photograph of our actual incubator. All images currently included in Figures 1 and 2 are original, have not been published elsewhere, and were created by the authors. We therefore confirm that these figures contain no copyrighted material.

"9. If the reviewer comments include a recommendation to cite specific previously published works, please review and evaluate these publications to determine whether they are relevant and should be cited. There is no requirement to cite these works unless the editor has indicated otherwise."

Thank you for this information and take note of it.

Reviewer 1:

We thank the reviewer for the helpful writing suggestions, which significantly improved the clarity and presentation of the manuscript.

Comment R1-1: *"Reviewer #1: 1. The use of the term "Exploring" in the title appears less appropriate. A more precise and assertive word, such as "Detection", may better reflect the nature of the study."*

Per comment of the reviewer, we have updated the title to: "Detection of non-invasive sexing of early chick embryos in intact eggs using laser speckle contrast imaging and deep neural networks"

Comment R1-2: *"2. First-person pronouns such as "we" should be avoided, in accordance with the conventions of scientific writing."*

Per comment of the reviewer, the use of first-person pronouns such as "we" have been significantly reduced thorough the manuscript.

Comment R1-3: *"3. The phrase "fold-wise p-value, p < 0.3" is unclear. Please clarify the intended meaning and context of this expression."*

We clarified that the reported p-values were calculated across cross-validation folds and modified the wording "~~fold-wise p-value~~" to "across cross-validation folds" to improve clarity.

We have also expanded the p-value explanation in the ResNeBit section to explicitly describe how these values were computed: "To assess whether the classification performance exceeded chance level, p-values were calculated across cross-validation folds (Table 2). For each fold, the p-value was computed using a binomial test under the null hypothesis of random guessing, implemented via a binomial calculator [65]. Specifically, the observed classification accuracy of the deep neural network (DNN) for each fold was compared against the expected accuracy from random classification of male versus female egg embryo LSCI images (i.e., a chance level of 50%). The significance levels α are established as follows: $p > 0.05$ (ns: not significant), $p < 0.05$ (*), $p < 0.01$ (**), $p < 0.001$ (***), and $p < 0.0001$ (****)."

The following text was updated in the Abstract: "Specifically, ResNetBiT had a five-fold cross-validated average accuracy of 56% ± 4% (p-value $p < 0.28$ across cross-validation folds) at day 3 and 57% ± 3% (p-value $p < 0.07$ across cross-validation folds) at day 4. YOLOv5s-cls had a five-fold cross-validated average accuracy of 55%±2% ($p < 0.13$ across folds) at day 3 and 57% ± 3% ($p < 0.10$ across folds) at day 4."

The following text was updated in the Introduction: "ResNetBiT achieved an average accuracy of 56% at day 3 (p-value $p < 0.28$ across cross-validation folds) and 57% at day 4 ($p < 0.07$ across folds). YOLOv5s-cls achieved an average accuracy of 55% at day 3 ($p < 0.13$ across folds) and 57% at day 4 ($p < 0.10$ across folds)."

Comment R1-4: *"4. Statistical significance should be reported using only the less than symbol ("<"), without the equal sign."*

The manuscript was revised to report statistical significance using only the less-than ("<") symbol for statistical significance. These revisions are reflected in the changes noted in the previous comment R1-3. We thank the reviewer for this comment.

Comment R1-5: *"5. It was initially assumed that the Deep Neural Network (DNN) used in the study was developed independently. However, the model appears to rely on existing algorithms. Please clearly state which parts of the architecture or training process were modified or uniquely implemented in the Methods section."*

We did not develop a completely new deep neural network (DNN) architecture for this study. Instead, we used established backbone DNN models and applied transfer learning with fine-tuning for our specific task of classifying male versus female egg embryo LSCI images. All modifications were limited to adapting the pre-trained networks to our dataset and task, including updating the output layer and retraining on our images. We have clarified this in the Methods section to ensure transparency regarding the architecture and training process.

Revisions: "For this study, established deep neural network (DNN) architectures were used. For the specific task of classifying male versus female egg embryo LSCI images, the DNN models were tuned with applied transfer learning and fine-tuning. Four DNN models were trained and tested for final selection: ResNetBiT (Big Transfer ResNet) (57), Xception41 (58), DenseNet121 (59), and InceptionV3 (54)."

Comment R1-6: *"6. The manuscript lacks a distinct discussion section. Including a well-structured discussion would greatly improve the clarity and scientific rigor of the paper.*

Per comment of the reviewer, we have added a Discussion section to the manuscript. This section provides a structured interpretation of our findings, including the limitations of the deep neural network (DNN) performance, the implications of the modest classification accuracy, a brief comparison with previously reported study, and directions for future research:

"Discussions

In this study, we evaluated the feasibility of using deep neural networks (DNNs) to classify the sex of chicken embryos from laser speckle contrast imaging (LSCI)-derived vascular images. Despite employing multiple established DNN architectures with transfer learning and fine-tuning, the overall classification performance remained modest, with the best-performing model achieving an accuracy of 61%. These results indicate that, under the current experimental and analytical conditions, sex classification at early-stages from vascular patterns alone is marginally above chance and not reliable. Importantly, these findings are consistent with the central conclusion of this work: sex-specific differences in vascular structure at the investigated developmental stages are either too subtle to be captured by LSCI or not present at all.

A key motivation for this paper was to contrast our findings with prior work (Ref. [16]), which reported classification accuracies exceeding 85% using similar vascular images (egg candling). Despite using a comparable methodological framework, we were unable to reproduce such high performance. The reasons for this discrepancy remain unclear. Notably, the dataset used in that study is not publicly available, and attempts to obtain additional details were unsuccessful, preventing direct comparison and reproducibility assessment.

Note that the choice to downsample images from 4096×3000 to 256×256 pixels was driven by compatibility with standard pre-trained DNN architectures and computational constraints. While this reduction in resolution may lead to loss of fine vascular details, experiments with higher input resolution (512×512) did not improve performance. Future work may explore alternative strategies, such as multi-scale approaches, higher-resolution DNN architectures, or image pre-processing techniques to better preserve and exploit the subtle vascular patterns. To promote transparency and enable further progress in this matter, we have made our dataset of original LSCI images (of size 4096×3000 pixels) publicly available, see Data availability section.

LSCI is particularly well suited for capturing hemodynamic and perfusion-related information (blood flow time trace). Exploration of temporal features, including embryonic heart rate and blood flow dynamics, did not reveal reliable sex-specific differences at early (days 3–4) developmental stages (see Supporting Information). At these stages, the embryonic heart remains

immature and has not yet developed into a fully structured four-chamber organ with a stable and regular beating pattern. Consequently, heart rate measurements exhibited inter-embryo variability, driven by differences in developmental stage rather than sex. In addition, heart rate measurements are sensitive to temperature fluctuations, where variations as small as ±1 °C were found to affect the measurements. Analysis at a later stage (day 6), where cardiac development is more advanced, was conducted on 39 eggs (20 female and 19 male). The temperature of the eggs was always above 37 °C. The results, shown in Supporting Figure S7, did not reveal clear sex-specific differences in heart rate between female and male, however, a larger dataset would be required to draw more definitive conclusions

Future studies could explore alternative approaches, such as multiple-instance learning to assess spatial and temporal variations in blood vessel patterns, feature extraction from LSCI images, blood flow dynamics and heart rate analysis, which would require precise temperature control during image acquisition for accurate measurements. These refinements may offer new insights into the potential for early-stage sex differentiation in chick embryos using LSCI imaging."

Reference:

16. Jia N, Li B, Zhao Y, Fan S, Zhu J, Wang H, et al. Exploratory Study of Sex Identification for Chicken Embryos Based on Blood Vessel Images and Deep Learning. Agriculture. 2023;13: 1480. doi:10.3390/agriculture13081480

Comment R1-7: *"The novelty of the research is not in question. However, the manuscript suffers from insufficient detail and overly simplistic explanations. These weaknesses reduce the overall impact of the study. Please revise the content thoroughly and align it with the standards of the journal."*

We thank the reviewer for acknowledging the novelty of our study and for highlighting areas where clarity and detail could be improved. We have thoroughly revised the manuscript to provide more detailed explanations of our methods, including the design and implementation of the classification deep neural networks, the cross-validation and p-value calculations, and the preprocessing and analysis of LSCI images. We have also refined the text throughout the Results and Discussion sections to provide clearer, more precise interpretations of the findings. We conducted major revisions to the manuscript addressing all comments from the four reviewers and the editorial team. As a result, we believe that the manuscript is improved in clarity, rigor, and overall impact, aligning it more closely with the standards of the journal.

Reviewer 2:

"The research methods and steps in this manuscript are generally accurate, and well-documented, aligning with standards for reproducibility and ethical conduct in developmental biology and imaging studies. Although the conclusions are negative, I believe this study is helpful for research and development in related fields. I have the following comments:"

We appreciate the reviewer's encouraging comments.

Comment R2-1: *"(1) After obtaining the laser speckle contrast image and BFI image, the authors only utilized the vascular structure information. Traditional methods, such as egg candling, provide more detailed vascular structure information, which may explain why the results of the tested method are inferior to those of the egg candling method. Please comment on this."*

While we agree that, in the present study, we primarily utilized structural information from the LSCI images, we do not agree that egg candling (brightfield imaging) provides more detailed vascular structure information than our LSCI images.

In our previous study (Ref. [21]), we directly compared LSCI and brightfield imaging using white chicken eggs, brown chicken eggs, and white quail eggs, and reported that both modalities provided comparable levels of vascular structural detail. Furthermore, we demonstrated that LSCI is less sensitive to confounding factors such as eggshell cracks, pigmentation, and surface irregularities, which can significantly degrade image quality and interpretation in brightfield imaging (see Figs. 2–4 in Ref. [21]). Furthermore, during early incubation (days 2–5), both modalities provided comparable visualization of vascular structures (Figs. 2–4 in Ref. [21]). LSCI measures blood flow dynamics, capturing functional vascular information that is not accessible with traditional egg candling. This enables visualization of perfusion-related features that are not detectable using purely intensity-based imaging.

Regarding using blood flow information for sexing eggs, please see our answer to comment R2-3.

Reference:

21. Dong Z, Mahler S, Readhead C, Chen X, Dickson M, Bronner M, et al. Non-invasive laser speckle contrast imaging (LSCI) of extra-embryonic blood vessels in intact avian eggs at early developmental stages. Biomed Opt Express. 2024 Aug 1;15(8):4605. doi:10.1364/BOE.530366

Comment R2-2: *"(2) When constructing the training dataset for deep learning, the authors downsampled all images from 2300×2300 to 256×256, which may result in the loss of vascular structure details. Please evaluate the impact of downsampling on vascular structure details."*

The input size of 256×256 pixels was chosen to match the requirements of the pre-trained models used in this study and to ensure computational feasibility, as processing the cropped 2300×2300 images would have required substantially higher computational resources. To evaluate the effect of image size, we also tested 512×512 images; however, this did not improve model performance and in some cases resulted in slightly worse classification accuracy. This suggests that increasing resolution alone does not necessarily enhance the model's ability to extract discriminative vascular features in this dataset. We agree that investigating strategies to preserve more fine vascular details while maintaining computational feasibility is an important direction for future work.

Revisions: We have added a paragraph in the discussion section to detail the choice of downsampling images to 256×256 pixels. We also acknowledged that downsampling may reduce fine vascular detail and that future work could explore alternative approaches such as multi-scale modeling or higher-resolution architectures:

"Note that the choice to downsample images from 4096×3000 to 256×256 pixels was driven by compatibility with standard pre-trained DNN architectures and computational constraints. While this reduction in resolution may lead to loss of fine vascular details, experiments with higher input resolution (512×512) did not improve performance. Future work may explore alternative strategies, such as multi-scale approaches, higher-resolution DNN architectures, or image pre-processing techniques to better preserve and exploit the subtle vascular patterns. To promote transparency and enable further progress in this matter, we have made our dataset of original LSCI images (of size 4096×3000 pixels) publicly available, see Data availability section."

Comment R2-3: *"(3) The advantage of laser speckle imaging lies not in structural imaging but in hemodynamic and perfusion imaging, such as capturing the periodic changes in blood flow caused by heartbeats. Please comment on whether hemodynamics can assist in non-invasive sexing of early chick embryos in intact eggs."*

We agree that the advantage of LSCI lies in capturing hemodynamic and perfusion-related information rather than static structural features. Accordingly, we did explore this direction by analyzing sex-related differences in embryonic heart rate and temporal blood flow graphs at days 3 and 4 of incubation. However, we did not observe any statistically significant differences between male and female embryos at these early stages. One major challenge was the high inter-egg variability in heart rate, which is particularly pronounced at those early stages of development. Indeed, at days 3 and 4 of incubation, the embryonic heart is still immature and has not yet fully developed into a well-structured four-chamber organ with a stable and regular beating pattern. As a result, variations due to differences in developmental stage appear to be larger than any potential differences associated with sex.

Another important limitation is the strong dependence of heart rate on temperature. We observed that even small temperature variations (as little as ± 1 °C) can lead to non-negligible changes in heart rate and blood flow dynamics. During imaging, eggs have to be removed from the incubator, requiring data acquisition to be completed within approximately 3 minutes to minimize temperature drop effects. To address this limitation, we are working on integrating an incubator directly into the LSCI system (Ref. [22]).

For completeness, we extended this analysis to a later stage (> day 6), where the heart is more developed, and recorded data from 39 eggs. We maintained the temperature of the eggs to be always above 37 °C for this experiment. Ground-truth sexing was done similarly than in the manuscript (PCR test + gonads visualization).

The results (Table R1 and Figure R2) still did not reveal clear or reliable sex-specific differences in heart rate. These findings suggest that, while hemodynamic features are a promising direction in principle, heart rate alone may not provide a sufficiently robust signal for early sex determination, particularly at very early developmental stages, which is why we focused on the blood vessels images.

Table R1: List of day-6 incubation chicken eggs for which blood flow was measured and heart rate was extracted for sex determination.

Egg ID	T egg (°C)	Heart rate (bpm)	Visual+PCR Sex
1410	> 37	190	F
1419	> 37	228	F
1421	> 37	232	F
1425	> 37	228	F
1428	> 37	228	F
1451	> 37	215	F
1462	37.6	215	F
1465	37.9	235	F
1466	37.8	235	F
1471	37.6	230	F

1472	38.0	205	F
1474	37.6	190	F
1476	37.5	215	F
1477	37.8	205	F
1478	37.0	215	F
1484	37.0	215	F
1485	37.3	225	F
1486	37.3	215	F
1488	37.6	220	F
1489	37.6	220	F
1411	> 37	215	M
1418	> 37	215	M
1420	> 37	241	M
1424	> 37	119	M
1450	> 37	241	M
1455	> 37	228	M
1463	38.2	225	M
1464	38.0	230	M
1468	37.2	235	M
1469	37.4	245	M
1470	36.9	225	M
1475	37.2	190	M
1476	38.0	177	M
1479	37.2	150	M
1480	37.5	220	M
1481	37.5	225	M
1482	37.1	155	M
1483	37.4	230	M
1490	37.6	185	M

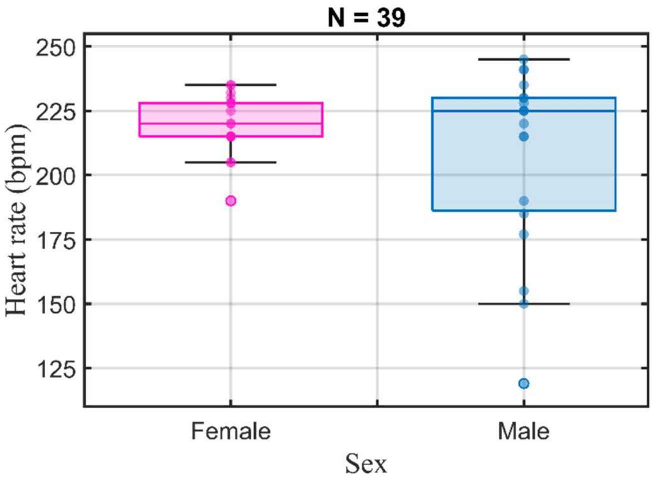

bpm	Female	Male
Average	218	208
Variance	13	35
Quartile Q1	215	188
Q2 (median)	218	225
Q3	228	230
Q4	235	245

Figure S7 (left) Statistical distribution of heart rate for male and female chicken embryos at day 6 of incubation. Each dot in the graph represents a chicken egg. (Right) Summary table showing mean, median, and quartiles of heart rate for each sex. As shown, there is no clear statistical difference between female and male in heart rate, even though the male distribution appears more dispersed.

We have added a paragraph in the Discussion section addressing the feasibility of sexing chicken eggs based on blood flow dynamics and heart rate at days 3–4 of incubation: "LSCI is particularly well suited for capturing hemodynamic and perfusion-related information (blood flow time trace). Exploration of temporal features, including embryonic heart rate (HR) and blood flow dynamics, did not reveal reliable sex-specific differences at early (days 3–4) developmental stages (see Supporting Information). At these stages, the embryonic heart remains immature and has not yet developed into a fully structured four-chamber organ with a stable and regular beating pattern. Consequently, heart rate measurements exhibited inter-embryo variability, driven by differences in developmental stage rather than sex. In addition, heart rate measurements are sensitive to temperature fluctuations, where variations as small as ±1 °C were found to affect the measurements.

Finally, analysis at a later stage (day 6), where cardiac development is more advanced was conducted on 39 eggs (20 female and 19 male). The temperature of the eggs was always maintained above 37 °C. The results, shown in Supporting Figure S7, did not reveal clear sex-specific differences in heart rate between female and male, however, a larger dataset would be required to draw more definitive conclusions."

Supporting Figure S7 was also added to the Supporting Information.

Reviewer 3:

"This paper "Exploring non-invasive sexing of early chick embryos in intact eggs using Laser Speckle Contrast Imaging (LSCI) and Deep Neural Network (DNN)" uses a custom laser speckle contrast imaging (LSCI) system to non-invasively capture extraembryonic blood vessel images from 1,251 chick embryos at days 3 and 4 of incubation, then applies ResNetBiT and YOLOv5 deep neural networks to determine whether sex can be classified from vascular patterns alone, concluding via five-fold cross-validation that neither model achieves practically useful accuracy (59–61% and 53–55% respectively), rendering the approach insufficient for reliable early sex identification.

The topic is justified. The paper could be further improved if the following remarks are taken into consideration:"

Our response: We thank the reviewer for the positive evaluation and constructive comments. We have revised the manuscript accordingly and provided detailed responses below.

<u>Comment R3-1:</u> *"1. The best-performing model (ResNetBiT at day 4) achieves only 61% accuracy, which is barely above chance — and only one-fold across both models and both days even marginally passes the significance threshold (p ≤ 0.04), making the entire DNN component statistically inconclusive and of no practical value."*

We thank the reviewer for this important comment and agree with the assessment. Indeed, the relatively modest performance of the best-performing model (<60% accuracy) supports the main conclusion of our study that sex classification of chicken embryos from LSCI-derived vascular images using current deep neural network (DNN) approaches remains inconclusive at this stage.

A primary motivation of this work is to contrast our findings with those reported in Ref. [16] (Agriculture, MDPI), where accuracies exceeding 88% were reported using similar vascular imaging data on a larger dataset. Despite our efforts, we were unable to reproduce or explain this discrepancy. Notably, the dataset from Ref. [16] is not publicly available and attempts to obtain further details from the authors were unsuccessful, limiting direct comparison.

After posting the manuscript on bioRxiv, the dataset was shared with three independent researchers, none of whom were able to achieve improved classification performance above 60%, which re-affirms our assessment that sexing chicken embryos from vascular images is inconclusive. Following reviewers and editorial comments, we have established a public repository for this dataset, enabling others to explore alternative methods and potentially improve our results.

<u>Revisions:</u> We added a Discussion section to interpret the limitations of the deep neural network (DNN) performance, the implications of the modest classification accuracy, and a comparison with the previously reported study. We also discuss potential reasons for the observed discrepancies, as well as directions for future research.

"In this study, we evaluated the feasibility of using deep neural networks (DNNs) to classify the sex of chicken embryos from laser speckle contrast imaging (LSCI)-derived vascular images. Despite employing multiple established DNN architectures with transfer learning and fine-tuning, the overall classification performance remained modest, with the best-performing model achieving an accuracy of 61%. These results indicate that, under the current experimental and analytical conditions, sex classification at early-stages from vascular patterns alone is marginally above chance and not reliable. Importantly, these findings are consistent with the central conclusion of this work: sex-specific differences in vascular structure at the investigated developmental stages are either too subtle to be captured by LSCI or not present at all.

A key motivation for this paper was to contrast our findings with prior work (Ref. [16]), which reported classification accuracies exceeding 85% using similar vascular images (egg candling). Despite using a comparable methodological framework, we were unable to reproduce such high performance. The reasons for this discrepancy remain unclear. Notably, the dataset used in that study is not publicly available, and attempts to obtain additional details were unsuccessful, preventing direct comparison and reproducibility assessment.

Note that the choice to downsample images from 4096×3000 to 256×256 pixels was driven by compatibility with standard pre-trained DNN architectures and computational constraints. While this reduction in resolution may lead to loss of fine vascular details, experiments with higher input resolution (512×512) did not improve performance. Future work may explore alternative strategies, such as multi-scale approaches, higher-resolution DNN architectures, or image pre-processing techniques to better preserve and exploit the subtle vascular patterns. To promote transparency and enable further progress in this matter, we have made our dataset of original LSCI images (of size 4096×3000 pixels) publicly available, see Data availability section.

LSCI is particularly well suited for capturing hemodynamic and perfusion-related information (blood flow time trace). Exploration of temporal features, including embryonic heart rate and blood flow dynamics, did not reveal reliable sex-specific differences at early (days 3–4) developmental stages (see Supporting Information). At these stages, the embryonic heart remains immature and has not yet developed into a fully structured four-chamber organ with a stable and regular beating pattern. Consequently, heart rate measurements exhibited inter-embryo variability, driven by differences in developmental stage rather than sex. In addition, heart rate measurements are sensitive to temperature fluctuations, where variations as small as ±1 °C were found to affect the measurements. Analysis at a later stage (day 6), where cardiac development is more advanced, was conducted on 39 eggs (20 female and 19 male). The temperature of the eggs was always above 37 °C. The results, shown in Supporting Figure S7, did not reveal clear sex-specific differences in heart rate between female and male, however, a larger dataset would be required to draw more definitive conclusions

Future studies could explore alternative approaches, such as multiple-instance learning to assess spatial and temporal variations in blood vessel patterns, feature extraction from LSCI images, blood flow dynamics and heart rate analysis, which would require precise temperature control during image acquisition for accurate measurements. These refinements may offer new insights into the potential for early-stage sex differentiation in chick embryos using LSCI imaging."

Reference:

16. Jia N, Li B, Zhao Y, Fan S, Zhu J, Wang H, et al. Exploratory Study of Sex Identification for Chicken Embryos Based on Blood Vessel Images and Deep Learning. Agriculture. 2023 Jul 26;13(8):1480. doi:10.3390/agriculture13081480

Comment R3-2: *"2. Both models exhibit severe overfitting, with training accuracy reaching 75–100% while validation accuracy stagnates near chance level, yet no effective regularization strategies beyond early stopping are explored or discussed."*

We thank the reviewer for this concern. We respectfully note that our original experiments already included several regularization strategies beyond early stopping: (1) extensive training-only data augmentation, systematically tuned through ablation experiments on rotation (up to ±180°, Figure S4), translation, scaling, and random flips, which demonstrably reduced the train-validation gap; (2) cosine annealing learning-rate scheduling; and (3) transfer learning from ImageNet-21k (ResNetBit)/ImageNet-1k(YOLOv5s-cls) pretrained weights. In the YOLOv5s-cls experiments, we additionally used weight decay (5×10^{-5}).

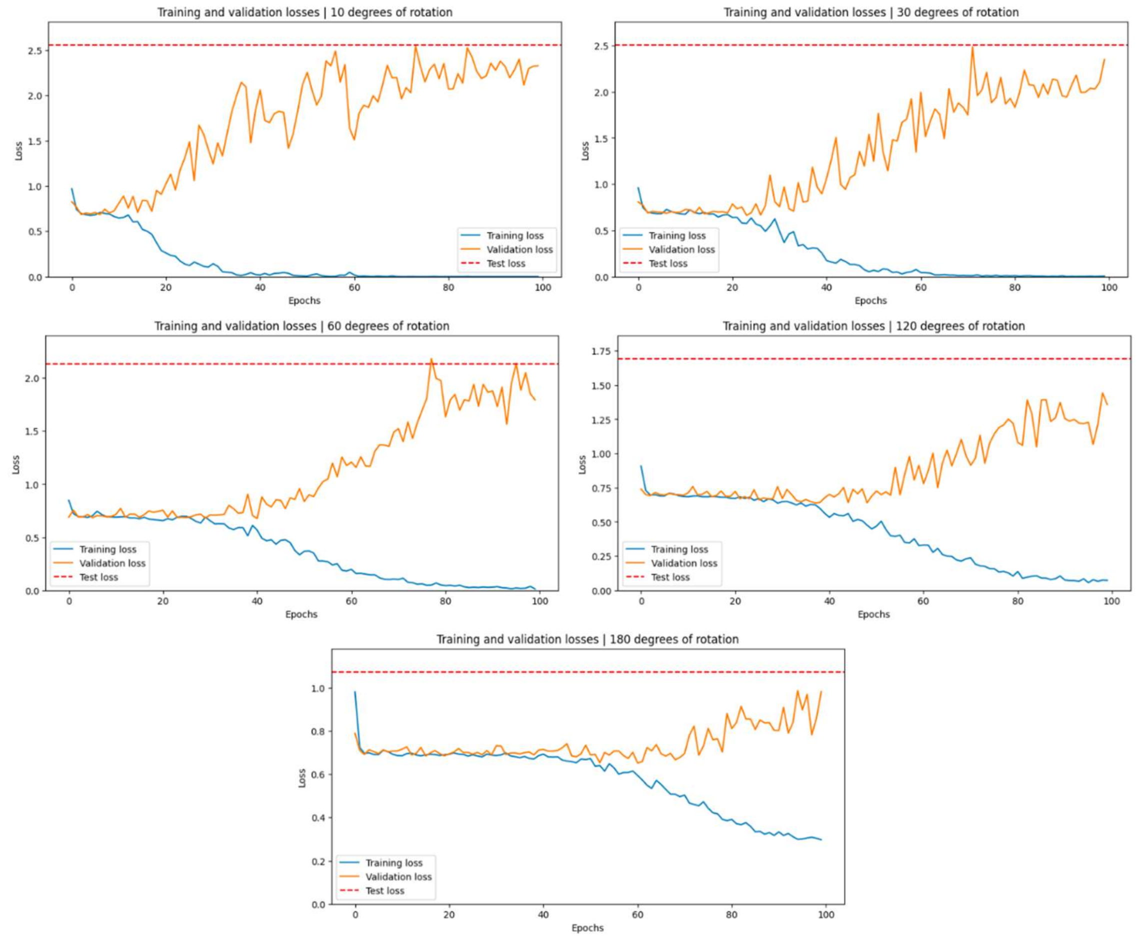

Figure S4: Training and validation loss curves for different levels of random-rotation augmentation (±10°, ±30°, ±60°, ±120°, and ±180°). Increasing rotation strength reduced the divergence between training and validation loss, consistent with decreased overfitting; the clearest effect was observed at ±180°.

In response to the reviewer's suggestion, we further strengthened regularization for both models. For ResNetBiT, we froze all backbone layers except the last two stages, added dropout (p = 0.3) and weight decay (1e-4), reduced the learning rate to 5e-5 with a 5-epoch linear warmup, and switched early stopping to ACC- and loss-based monitoring from epoch 11. For YOLOv5s-cls, we froze backbone layers 0–9, added dropout (p = 0.1), enabled on-the-fly augmentation (random flipping, rotation, and color jitter), and applied early stopping with patience of 15 after a 20-epoch warmup. These changes substantially reduced the train–validation gap in both models (see updated Figures 4 and 5). Despite these measures, validation performance remained near chance across architectures. Thus, although overfitting could be mitigated, reliable generalization was not recovered. We therefore interpret the limited generalization as more consistent with a weak or non-robust sex-discriminative signal in the data than with insufficient regularization alone.

Per comment of the reviewer, we have added Figure S4 to the Supporting Information and revised the text in the “Approaches: ResNetBit and YOLOv5” section of the Materials and methods:

“Both models were trained using the Adam optimizer, BCE loss function, and cosine annealing learning rate scheduler to ensure stable convergence. To prevent overfitting, several

regularization strategies were employed. `, we froze all backbone layers except the last two stages, added dropout (p = 0.3) and weight decay (1e-4), and used a learning rate of 5e-5 with a 5-epoch linear warmup followed by cosine annealing. Early stopping was applied from epoch 11 onward using two criteria: validation ACC (patience = 10, minimum improvement = 0.01) and validation loss (patience = 5). For YOLOv5s-cls, we froze backbone layers 0–9, added dropout (p = 0.1), and applied weight decay (5×10^{-5}) with a cosine annealing learning rate scheduler. Early stopping with a patience of 15 epochs was applied after a 20-epoch warmup. Both models used training-only data augmentation including rotations (up to ±180°), translations, scaling, random flips, and brightness/contrast jitter, as well as transfer learning from ImageNet pretrained weights. Five-fold cross-validation with egg-ID-based splits was used to prevent data leakage and ensure reproducibility."

"For both ResNetBit and YOLOv5s-cls models, several regularization strategies were applied exclusively to the training set to improve model generalization and prevent overfitting (see Supporting Information Fig S4). Training-only data augmentation included included random rotations up to 180°, translations up to 5% in any direction, image scaling between 0.85x and 1.15x, random vertical and horizontal flips with probability 0.3, and brightness/contrast/saturation jitter. No augmentations were applied to the validation set. Five-fold cross-validation with egg-ID-based splits were implemented to prevent data leakage and evaluate the model's performance.

For ResNetBiT, all backbone layers were frozen except the last two stages, dropout (p = 0.3) and weight decay (1e-4) were added, and the learning rate was set to 5e-5 with a 5-epoch linear warmup followed by cosine annealing. Early stopping was applied from epoch 11 onward using two criteria: validation ACC (patience = 10, minimum improvement = 0.01) and validation loss (patience = 5), halting training when either criterion was triggered.

For YOLOv5s-cls, backbone layers 0–9 were frozen, dropout (p = 0.1) was added to the classification head, and weight decay (5×10^{-5}) was applied. Early stopping with a patience of 15 epochs was applied after a 20-epoch warmup. This strategy ensured that training was stopped before training and validation performance significantly diverged (Fig. S4)."

<u>Comment R3-3:</u> *"3. Using YOLOv5 — an object detection architecture — for binary classification without bounding-box labels or region proposals, is a fundamental architectural mismatch that is inadequately justified and likely contributes to its below-random performance at day 4 (53%)."*

We thank the reviewer for this important observation and apologize for the confusion. We would like to clarify that our implementation used the classification variant of YOLOv5, YOLOv5s-cls, rather than its object detection head. Therefore, the concern regarding the use of a bounding-box-supervised detector without corresponding annotations does not apply in our case. We selected this architecture based on prior work [16], which reported strong performance using YOLO-based models in related imaging tasks. To ensure a fair assessment, we also evaluated several widely used convolutional neural network classifiers, including ResNetBiT, Xception41, DenseNet121, and InceptionV3. However, all architectures consistently exhibited poor validation performance on our dataset. Importantly, the uniformly low accuracy observed across both YOLOv5s-cls and conventional classification architectures suggests that the limitation is unlikely to stem from architectural mismatch alone. Rather, these findings support our central conclusion that vascular pattern imaging, by itself, does not provide a sufficiently robust signal for reliable early sex identification in chicken embryos.

Revisions: We added clarifications to the first paragraph of the section: "Approaches: ResNetBit and YOLOv5s-cls" in Materials and methods:

"To classify the sex of chicken embryos, two established backbone DNN architectures were used: ResNetBiT (51) and YOLOv5s-cls (58), both adapted for binary classification. ResNetBiT is a high-capacity convolutional neural network designed for robust feature extraction and transfer learning. Pretrained on the ImageNet-21k dataset, it is well-suited for transfer learning on small datasets. ResNetBiT was initialized with pretrained weights and fine-tuned it on our dataset. Similarly, YOLOv5s-cls was adapted using its classification variant, rather than its object detection head. This distinction is important, as the model was not used with bounding-box supervision but instead configured as a standard image-level classifier. This approach allowed to explore whether a detection-based model, originally designed for localization tasks, could better suit this problem. The YOLOv5s-cls model was trained on 640 x 640 pixels images to match the default image size for the model."

We also clarified that the YOLOv5s-cls variant of YOLOv5 was used in this study.

Reference:

16. Jia N, Li B, Zhao Y, Fan S, Zhu J, Wang H, et al. Exploratory Study of Sex Identification for Chicken Embryos Based on Blood Vessel Images and Deep Learning. Agriculture. 2023 Jul 26;13(8):1480. doi:10.3390/agriculture13081480

Comment R3-4: *"4. The initial model selection procedure used a simple train/test split rather than cross-validation, and ResNetBiT was selected despite having the lowest accuracy (51%), solely based on loss behavior, which is a subjective and unreliable selection criterion."*

While the initial model comparison was performed with a simple train/test split, we also employed cross-validation for parameter tuning to ensure robust hyperparameter selection.

Figure S6 shows the training and validation curves for ResNetBiT (fist row), Xception41 (second row), DenseNet121 (third row), and InceptionV3 (last row) over 25 epochs on the full dataset (HH19 + HH25). Although ResNetBiT initially exhibits the lowest accuracy, it showed the most balanced learning behavior: training and validation losses remain relatively close, whereas Xception41, DenseNet121, and InceptionV3 exhibited decreases in training loss but stagnant or increasing validation loss, indicating overfitting, thus minimal remaining learning capabilities. Similarly, only ResNetBiT demonstrated progressive improvements in validation accuracy and AUC, while the other architectures plateaued or declined.

Based on these observations, we concluded that ResNetBiT had the greatest potential for further improvement, and thus it was selected for extended training and fine-tuning. This selection criterion is therefore not solely subjective but grounded in empirical potential improvements.

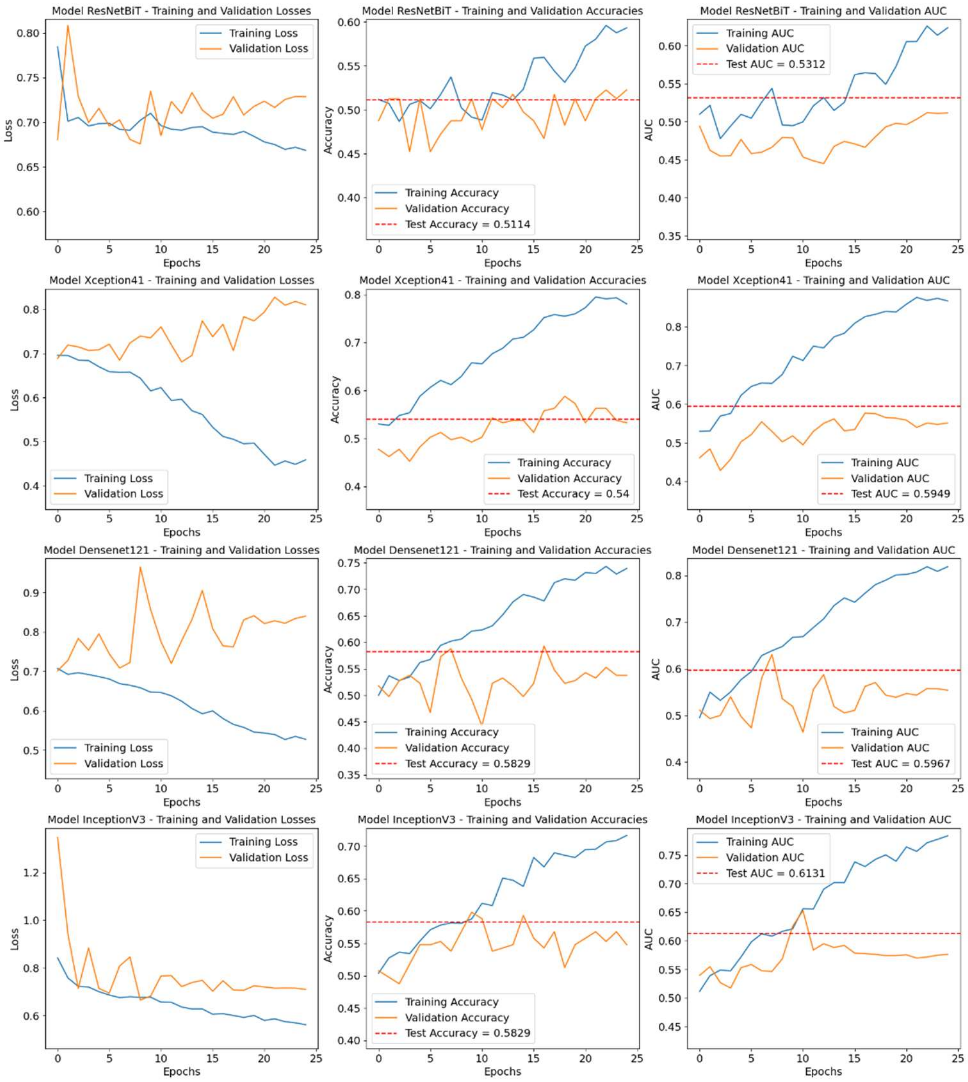

Figure S6: Training and validation curves for ResNetBiT, Xception41, DenseNet121, and InceptionV3 over 25 epochs on the full dataset (HH19 + HH25). Although ResNetBiT performs the worst in terms of accuracy, it exhibits the least overfitting, with training and validation losses relatively aligned, suggesting higher potential for learning with extended training.

<u>Revisions:</u> We have added a Support Figure S6 in the Supporting Information and revised the Choice of the Deep Neural Networks (DNNs) section to better justify the choice of ResNetBit:

"After training and validating the four DNNs for 25 epochs, the accuracy, AUC, and loss for each model were as follows: ResNetBiT (accuracy: 51%, AUC: 0.51, loss: 0.72), Xception41 (accuracy: 54%, AUC: 0.54, loss: 0.80), DenseNet121 (accuracy: 53%, AUC: 0.54, loss: 0.85), and InceptionV3 (accuracy: 55%, AUC: 0.57, loss: 0.76). Although ResNetBiT performed slightly worse in terms of accuracy and AUC, it exhibited the least overfitting with lowest loss and with training and validation curves showing similar trends (see Supporting Information). This observation suggests that ResNetBiT may have greater capacity to improve if trained for additional epochs, motivating our focus on optimizing ResNetBiT performance,"

Comment R3-5: *"5. The ResNet50 and YOLOv8 validation mentioned in Section 2.3 is described in a single paragraph with no quantitative results, no tables, and no methodology detail, rendering it scientifically uninformative and insufficient to constitute independent validation."*

We agree with the reviewer that the original description of the ResNet50 and YOLOv8 validation was insufficient to constitute an independent evaluation. We have added more details about the methodology and results:

"Additionally, the classification performance was independently validated using a modified ResNet50 model and a YOLOv8-nano classification model. The system processes egg images from both day 3 (HH19) and day 4 (HH25) developmental stages, supporting training on individual stages as well as a mixed-stage dataset. For the ResNet50 model, the architecture was adapted for single-channel grayscale input by modifying the first convolutional layer (Conv2d, 1→64 channels, 7×7 kernel, stride 2, padding 3) and adjusting the final fully connected layer 2048→2 outputs. Models were trained both with ImageNet-pretrained weights and from random initialization. Input images were resized to 224×224 pixels and subjected to data augmentation (random resized cropping and horizontal flipping), followed by normalization ($\mu = 0.5$, $\sigma = 0.5$). Optimization was performed using the Adam optimizer (learning rate of 10^{-3}) with cross-entropy loss for 25 epochs with a batch size of 32. For the YOLOv8 model, we used the nano classification variant (yolov8n-cls), rather than the object detection architecture, to perform image-level binary classification. The model was trained on 512×512 pixel images with a batch size of 16 for 50 epochs."

Experimental design:

"Both architectures were evaluated across three dataset configurations: HH19 only (day 3; n=1,287 images), HH25 only (day 4; n=1,048 images), and a mixed dataset combining both stages (n=2,335 images). For both architectures, the dataset was split into training and validation sets using an 80/20 stratified split based on sex labels, across HH19, HH25, and mixed-stage conditions. All experiments were tracked using weights and biases to ensure reproducibility and consistent performance monitoring. To account for split variability, all experiments were repeated across five independent random seeds and results are reported as mean ± standard deviation of the best validation accuracy (Supporting Table S1). Both models achieved validation performance close to chance level across configurations, consistent with the results obtained from other architectures. These findings further support the conclusion that LSCI vascular pattern alone does not provide a sufficiently robust discriminative signal for reliable early sex identification."

We added the following Supporting Table S1 summarizing the independent validation results into the Supporting Information:

"G- Results on independent validation with ResNet50 and YOLOv8n-cls DNN models

Table S1. Binary sex classification accuracy (%) for ResNet50 and YOLOv8 across developmental stages, reported as mean ± std over 5 train/validation splits.

Model	Mixed (HH19+HH25)	HH19 (day 3)	HH25 (day 4)

ResNet50 (ImageNet pretrained)	52.1 ± 1.2	51.3 ± 0.8	52.0 ± 1.6
ResNet50 (from scratch)	52.1 ± 1.3	51.4 ± 1.1	52.0 ± 1.6
YOLOv8n-cls	56.8 ± 1.4	56.5 ± 1.4	58.8 ± 4.3
Chance level	51.6	51.2	51.9

No model configuration achieved classification accuracy meaningfully above chance level (Table S1). ResNet50 models converged to near-identical performance regardless of pretraining strategy, consistent with both architectures learning a trivial majority-class prediction. YOLOv8 exhibited marginally higher accuracy (56.8–58.8%) but with considerable variance across splits (up to ±4.3%), suggesting sensitivity to train/validation partitioning rather than genuine discriminative learning. These results corroborate the primary DNN findings and confirm that standard image classification architectures are unable to reliably identify embryo sex from LSCI images at these developmental stages."

Comment R3-6: *"6. The paper cannot explain the 24–32 percentage point accuracy gap between its results and those of Ref. [13] using a comparable protocol and dataset size, and the inability to access Ref. [13]'s dataset or code means this critical discrepancy remains entirely unresolved."*

We thank the reviewer for raising this important point. We agree that, at present, there is no clear or satisfactory explanation for the discrepancy in accuracy between our results and those reported in previously submitted version Ref. [13] (Ref. [16] in newly version), despite the apparent similarity in experimental protocol and data analysis. We made efforts to better understand this discrepancy by requesting access to the dataset and implementation details from the authors of Ref. [16]. Unfortunately, we did not receive a response, which limits our ability to perform a direct comparison or identify the underlying causes of the performance difference.

In this context, we believe that the value of our study is important in providing an independent benchmark using similar experimental framework. Our results provide an additional data point and contribute to a more balanced assessment of the feasibility of early sex classification based solely on vascular patterns. As such, our study highlights the need for caution when interpreting the results of Ref. [16] and underscores the importance of reproducibility and independent validation in this area.

Comment R3-7: *"7. The heart-detection heuristic — identifying the embryonic heart as the brightest 30×30 pixel region — is not validated against ground truth anatomical positions, and misidentification would systematically misalign all downstream crops, corrupting the entire dataset.."*

We do not agree with the reviewer's concern regarding the heart-detection being heuristic and not validated against ground truth anatomical positions. As opposed to conventional egg candling (Ref. [16]), our LSCI system captures anatomical structure via relative blood flow measurements (see Ref. [21]) through speckle contrast analysis. Specifically, the blood flow index (BFI) is extracted from the speckle contrast as:

$$BFI = \frac{1}{K^2}$$

The BFI is a quantitative proxy for relative blood flow measurements: higher BFI values indicate faster-moving blood and greater perfusion, while lower values correspond to slower flow. In developing embryos, the heart is the region with the highest vascularization and blood circulation, and therefore consistently exhibits the highest BFI values. As a result, the brightest 30×30 pixel region in LSCI images corresponds, for healthy embryos, to the embryonic heart. To further support this assumption, we have included 16 representative LSCI images (see Figures R1 and R2 below), where the detected brightest region is marked with a red box. As shown, this region spatially overlaps with the expected anatomical location of the heart.

Importantly, this approach was not introduced without prior validation. In our previous work, Ref. [21], we directly validated the correspondence between LSCI signal intensity and anatomical structures by comparing LSCI images with microscopy images of the same embryos after top-eggshell removal (see Fig. 7 of Ref. [21]). We refer the reviewer to Visualization 1 of the Supplementary Material in Ref. [21], which shows that the brightest region in the LSCI image aligns with the embryonic heart.

Finally, additional support is provided by the heatmaps provided in newly added Supporting Figure S4 in response to Comment R3-11, where we showed that our ResnetBit model attention is focused on embryo-associated vascular regions rather than arbitrary locations. Taken together, these validations demonstrate that the brightest-region reliably identifies the embryonic heart, and therefore does not introduce systematic misalignment or corruption in downstream cropping.

Revision: Following the reviewer's comment, we have revised the Data Preparation and Preprocessing sections to more clearly state that the 30 × 30 pixel region with the highest BFI corresponds to the embryonic heart: "To enhance vascular structure, a sliding window approach was used to identify the 30 × 30 pixels region with the highest blood flow index (BFI), derived from speckle contrast. Given that the embryonic heart exhibits the highest perfusion in LSCI images (16,17), this region was used as a proxy for heart localization. Based on this localization, a 2,300 × 2,300 pixels square region was cropped and centered on the detected location, providing a standardized and biologically relevant region of interest across all samples."

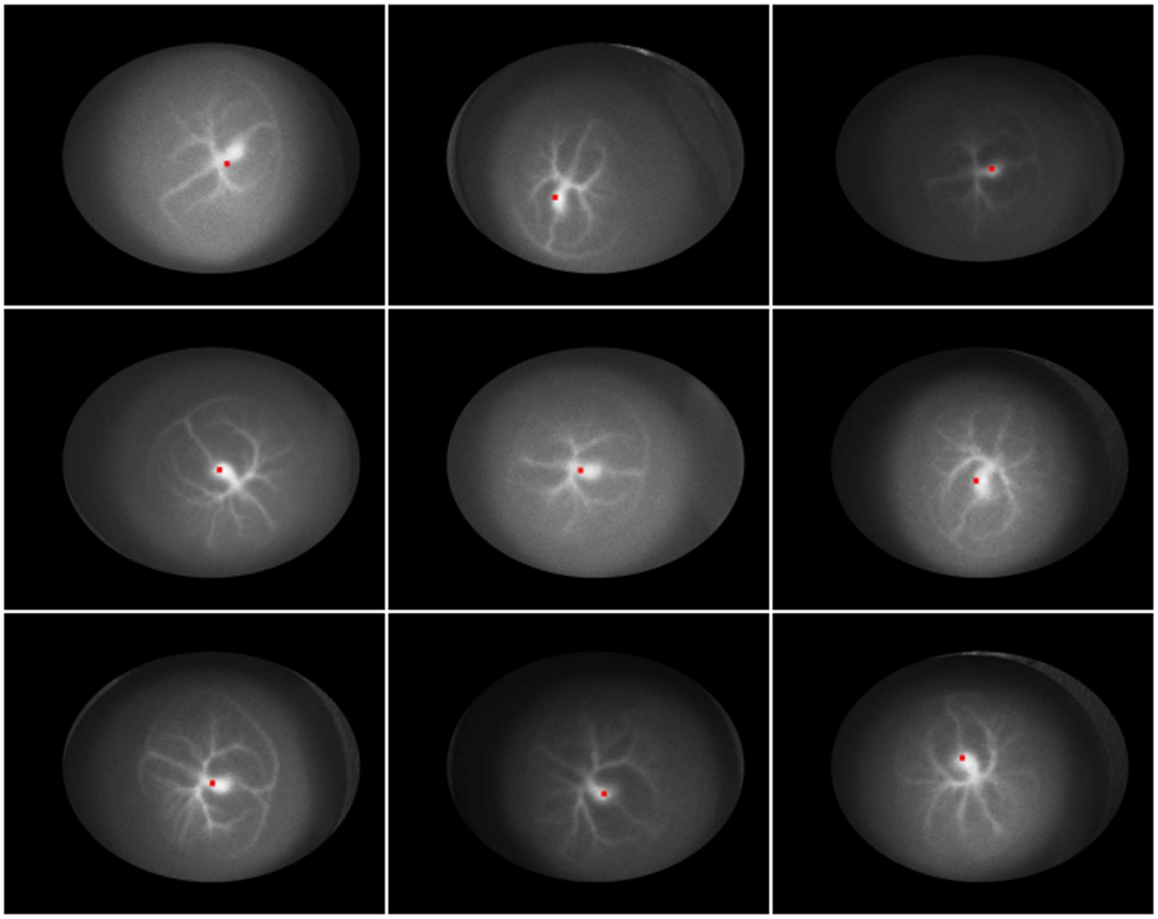

Figure R1: Nine randomly selected HH19 images with the brightest 30 × 30 pixel region highlighted by a red square.

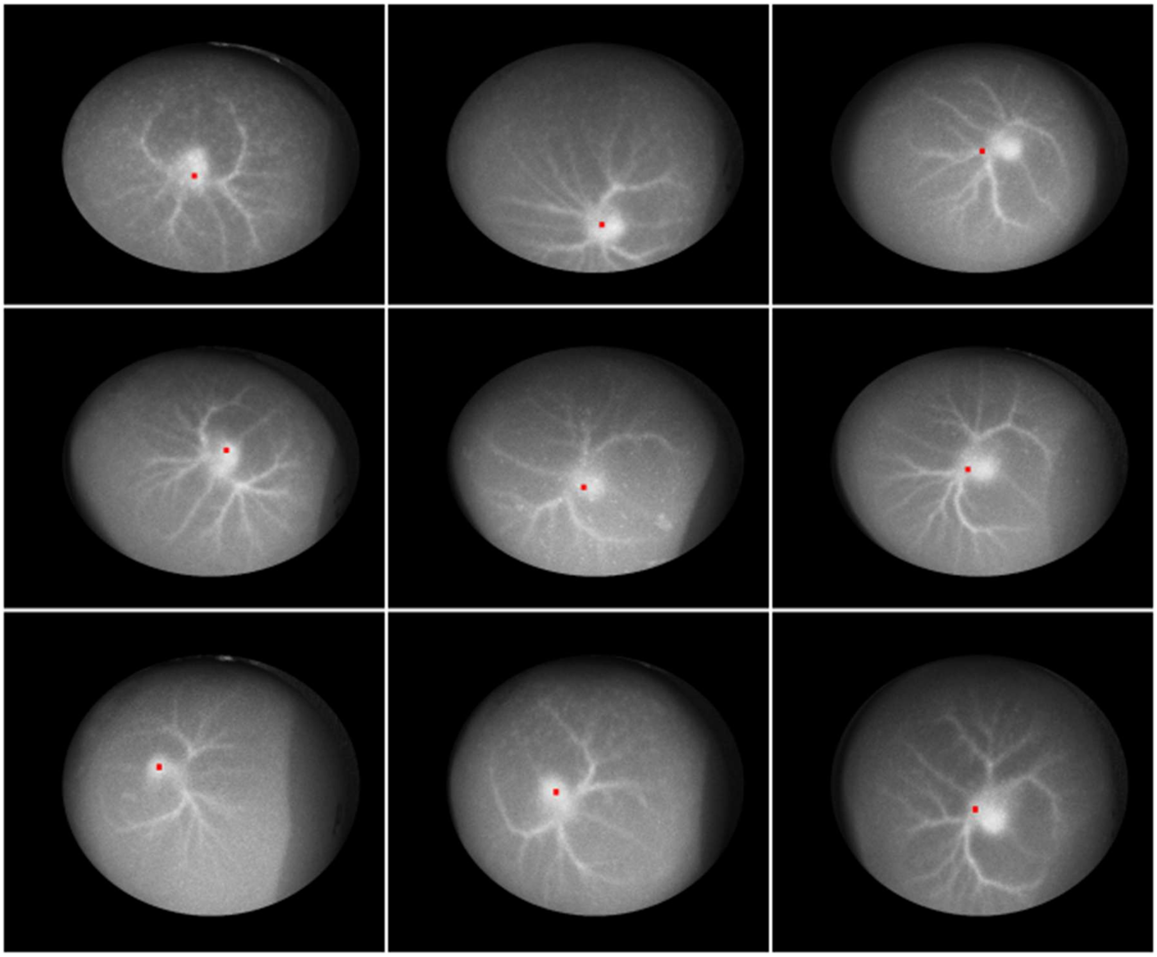

Figure R2: Nine randomly selected HH25 images with the brightest 30 × 30 pixel region highlighted by a red square.

References:

16. Jia N, Li B, Zhao Y, Fan S, Zhu J, Wang H, et al. Exploratory Study of Sex Identification for Chicken Embryos Based on Blood Vessel Images and Deep Learning. Agriculture. 2023 Jul 26;13(8):1480. doi:10.3390/agriculture13081480

21. Dong Z, Mahler S, Readhead C, Chen X, Dickson M, Bronner M, et al. Non-invasive laser speckle contrast imaging (LSCI) of extra-embryonic blood vessels in intact avian eggs at early developmental stages. Biomed Opt Express. 2024 Aug 1;15(8):4605. doi:10.1364/BOE.530366

22. Readhead C, Mahler S, Dong Z, Sato Y, Yang C, Bronner ME. Automated non-invasive laser speckle imaging of the chick heart rate and extraembryonic blood vessels and their response to Nifedipine and Amlodipine drugs. Dev Biol. 2025 Mar;519:46–54. doi:10.1016/j.ydbio.2024.12.005

Comment R3-8: *"8. The number of images per egg is non-uniform and largely undisclosed, and the paper's claim that this has "little impact on model performance" due to transfer learning is asserted without any supporting evidence or sensitivity analysis.."*

We thank the reviewer for raising this and agree that, ideally, each egg should be represented by a single image per classification stage to avoid potential sampling bias. In preliminary experiments, we evaluated this approach; however, using only one image per egg resulted in similarly low classification accuracy and did not improve model performance (accuracy

being consistently less than 60%). We adopted multiple images per egg to remain consistent with the protocol used in Ref. [16] (which also includes multiple images per egg, see their Tables 1 and 2), enabling a fairer comparison in terms of dataset size.

Revision: We acknowledge that the statement "*has little impact on model performance*" is not adequate and misleading. We have removed this statement from the manuscript to avoid overinterpretation. We thank the reviewer for highlighting this issue and have revised the text accordingly to more accurately reflect the limitations of our analysis: "The number of images collected per egg was not uniform due to the limited time windows available for imaging, as the eggs can only be imaged during specific time windows. In addition, some images had to be rejected due to poor imaging conditions, such as bad embryo positioning, embryonic body movements, or artifact noise. Ideally, each egg would be represented by a single image per classification stage to avoid potential sampling bias; however, in preliminary experiments, using only one image per egg yielded low classification accuracy (consistently below 60%). To enable a fair comparison (comparable dataset size) with the prior work in Ref. [16] (which also used multiple images per egg) multiple images per egg was used."

Comment R3-9: *"9. The data availability statement contradicts PLOS ONE policy by restricting access to data "upon reasonable requests from the corresponding author" rather than making it publicly available at the time of publication."*

Our response: We thank the reviewer for highlighting this issue. In accordance with the comments from the editorial team and reviewers 3 & 4, we have now made the dataset and code publicly available through a repository hosted on Zenodo, one of the repositories recommended by PLOS ONE. The repository can be accessed at:

Dataset URL:

https://zenodo.org/records/19004322?token=eyJhbGciOiJIUzUxMiJ9.eyJpZCI6IjYwZTdiMGVjLTMyOWEtNDY5MS05YTVjLWQ0OTU1ZDQzNmY0OCIsImRhdGEiOnt9LCJyYW5kb20iOiIzZjQwNzlhZDY5NTJjNTdiNzY4NTljNGQ5Y2M1ZTBkZCJ9.tIFalcb3V9aCwCLno5uCitBhZFBinbXC6pnOZzHFka6EPekYvE56muUQfPyiaNRORSbfIW3Q3CG23RxTZkc0nQ

Code URL: https://github.com/anika24/lsci-egg-sex-classification

In the initial submission, the dataset and code were not publicly deposited due to their large size (approximately 30 GB), as many common repositories impose storage limits (typically around 5 GB). We appreciate the reviewers' guidance in identifying suitable repositories recommended by PLOS ONE, which enabled us to select the only platform that supports > 25 GB datasets.

We also note that, following the posting of this work on bioRxiv, we received four data requests (three from affiliated researchers and one from an independent researcher), all of which were answered within one week and data provided.

Revision: The Data Availability Statement has been updated accordingly to reflect public access to the dataset and code.

Comment R3-10: *"10. The conclusion proposes CAM-based imaging from day 6 onward as a future direction yet acknowledges the current LSCI system is limited to day 5.5, meaning the most scientifically promising hypothesis cannot be tested without a hardware redesign that is not described or planned."*

Our response: We thank the reviewer for this insightful comment. As identified in our previous study (Ref. [21]), the primary limitation of LSCI imaging beyond day 5 arises from increased embryonic body motion, which degrades the accuracy of speckle contrast calculations or blood flow imaging. In the current system, images are acquired using a Thorlabs CS126MU Kiralux camera at 20 frames per second (FPS), corresponding to a 50 ms interval between frames (note that we used an exposure time of 10 ms per frame). Because LSCI requires approximately 100 frames per measurement, the total acquisition time is about 5 seconds. During this period, the embryo must remain relatively motionless to ensure reliable measurements. This condition is generally satisfied before day 5, when the embryo is still relatively small. However, beyond day 5, increased embryonic activity introduces motion artifacts that affect image quality.

To address this limitation, we are planning to upgrade the imaging system with a higher-speed camera (Lucid ATX124S-MC, Sony IMX535 sensor) capable of operating at 90 FPS, which is more than four times faster than the current system. This improvement reduces the inter-frame interval to approximately 11 ms and shortens the total acquisition time for 100 frames to about 1.1 seconds. As a result, the likelihood of motion-induced artifacts is substantially reduced. We therefore expect that this hardware improvement will enable reliable LSCI imaging at day 6 and beyond, allowing us to investigate the CAM-based imaging approach proposed in the conclusion. We have clarified this point in the revised manuscript.

Revisions: "While we hypothesize that sex differentiation may be detectable from day 6 onward, our current LSCI system is limited to imaging up to day 5.5 due to increased embryonic motion, which degrades speckle contrast measurements during multi-frame acquisition (total acquisition time of 5 s for 100 frames at 20 FPS, 10 ms exposure). To address this limitation, we plan to upgrade to a higher-speed camera (90 FPS), reducing the inter-frame interval to 11 ms and the total acquisition time to approximately 1.1 s, minimizing motion-induced artifacts. This improvement is expected to enable reliable LSCI imaging at day 6 and beyond."

Reference:

21. Dong Z, Mahler S, Readhead C, Chen X, Dickson M, Bronner M, et al. Non-invasive laser speckle contrast imaging (LSCI) of extra-embryonic blood vessels in intact avian eggs at early developmental stages. Biomed Opt Express. 2024 Aug 1;15(8):4605. doi:10.1364/BOE.530366

Comment R3-11: *"11. No feature visualization, saliency mapping, or interpretability analysis is provided, leaving it entirely unclear whether the models are responding to biologically meaningful vascular features or to imaging artefacts such as eggshell staining, embryo positioning, or brightness variation."*

We thank the reviewer for this important point. To address model interpretability, we included gradient-weighted class activation mappings (Grad-CAM) to provide visual heatmaps in Figure S5, highlighting the specific regions of the blood vessels image that influence our ResNetBiT model. Grad-CAM images were computed from the final conv3 layer in the last ResNet stage using the standard gradient-weighted class activation mapping procedure.

As shown in Figure S5, the resulting heatmaps show that model attention is generally concentrated within the egg and often around the embryo/vascular region, rather than exclusively on the external background. Specifically, high-activation regions did not consistently coincide with eggshell boundaries or background brightness gradients, suggesting that the model is not simply responding to shell staining or illumination artefacts. Furthermore, our extensive rotation augmentation (±180°) during training mitigates the risk that predictions are driven by embryo orientation.

The activation patterns are spatially diffuse and not consistent across samples, and they do not reveal a stable sex-specific vascular or anatomical signature. Therefore, while the visualization

reduces the concern that predictions are driven purely by imaging artefacts, it further supports our conclusion that the LSCI vascular images at HH19–HH25 do not contain sufficient sex-discriminative signal for reliable classification.

Per comment of the reviewer, we have added the following figure:

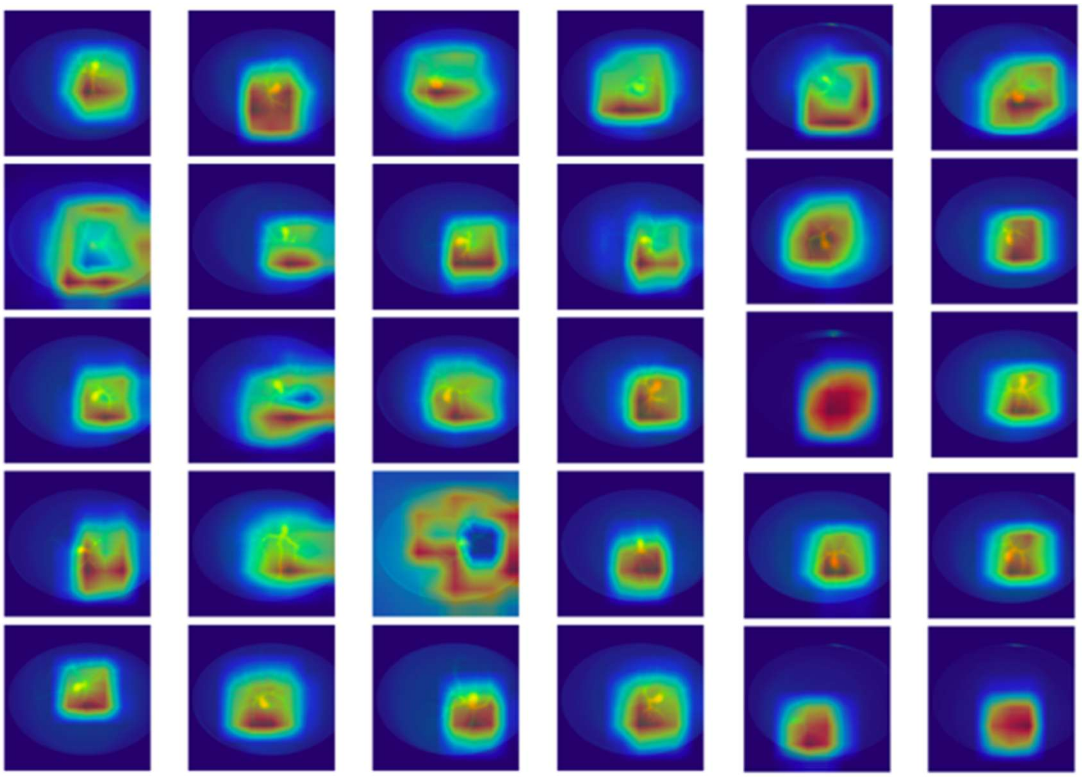

Figure S5: Gradient-weighted class activation mappings (Grad-CAM) heatmap visualizations of the early-stopped ResNetBiT model trained on LSCI egg images, highlighting image regions contributing to model predictions.

We also have added the following paragraph in the end of the ResnetBit section:

"To assess model interpretability, we applied gradient-weighted class activation mappings (Grad-CAM) to generate heatmaps highlighting regions influencing the ResNetBiT predictions, see Supporting Information Fig. S5. The model's attention was primarily localized within the egg, often near the embryo vascular region, rather than the external background, suggesting that the model was driven by the blood vessels structure rather than the eggshell artefacts or illumination bias. Despite focusing on biologically relevant regions, the model did not capture meaningful sex-discriminative features, supporting our conclusion that our LSCI images at HH19–HH25 were not suitable for DNN classification."

Comment R3-12: *"12. The paper frames itself as an exploratory study yet draws definitive conclusions ("sex identification from LSCI images at this early incubation stage is likely not feasible") from null results obtained with potentially suboptimal models on a modality that has not been fully optimized, overstating the negative conclusion beyond what the evidence supports."*

We thank the reviewer for assuring consistency of our manuscript. We agree that the original wording of our conclusion may have been too definitive given the exploratory nature of

this study. Our intent was to report that, under the current experimental conditions and modeling approaches, we did not observe sufficiently strong predictive performance to support reliable sex classification at this early incubation stage. In the revised manuscript, we have moderated our language to better reflect this scope and now emphasize that our findings indicate a lack of feasibility under the present imaging and modeling framework. These revisions have been incorporated into the Abstract and Conclusion sections.

Revisions:

Abstract: "~~Our findings suggest that using vascular pattern imaging alone is inconclusive for reliable early sex identification in chicken embryos.~~ Our findings suggest that under the current experimental conditions and modeling approaches, per-egg evaluation did not produce sufficiently accurate or statistically robust results for early sex classification."

Conclusion: "A per-egg evaluation approach did not achieve high accuracy or statistically robust results, indicating that, under the current experimental conditions and modeling approaches, we did not observe sufficiently strong predictive performance to support reliable sex classification at early incubation stages. We emphasize that this is not a definitive conclusion. Further refinements and optimizations of the models or alternative imaging approaches could potentially improve performance."

Reviewer 4:

Comment R4-1: *"1. The central assumption that vascular patterns encode sex information at day 3-4 is is biologically unlikely. Authors should explicitly discuss developmental biology constraints earlier in the introduction."*

We thank the reviewer for this important point which improves our introduction section. We agree that the biological plausibility of sex-specific vascular patterns at early stages is low, especially after knowing that sex related gene expression appears around day 4.5. The hypothesis of this study was: *"sex-specific vascular patterns appear before the sex gene expression"*.

As we said in the conclusion, the survey in Ref. [23] on gene expression during embryonic development indicate that key sex-determining genes begin to show differential expression around day 4.5 of incubation in male embryos and around day 6.5 in female embryos. Morphological differentiation of the gonads becomes observable only after approximately day 6.5, marking the onset of visible sex-specific development. Given that LSCI primarily captures vascular dynamics, these developmental timelines suggest that sex-related vascular differences are unlikely to be pronounced at days 3–4 (HH19–HH25). Instead, such differences may only emerge at later stages, potentially from day 6 onward, as genetic and morphological sex differentiation progresses. We have incorporated this discussion in the Introduction to clarify the biological limitations underlying our study:

"Recently, deep neural network (DNN) based studies have reported positive sex identification results using egg candling images of chick embryos [8,14,16–20]. Egg candling is a well-established technique in which an egg is held up to a strong light source to reveal its blood vessel network due to the high optical absorption of hemoglobin (in contrast to laser speckle contrast imaging (LSCI) [21,22] that provides blood flow). A recent study [16] suggest that DNN models can pick-up the subtle sex-based differences in the blood vessel network at early stages (as early as day 3 of incubation). However, these results are not supported by biological plausibility. Studies on embryonic gene expression indicate that key sex-determining genes begin to show differential expression around day 4.5 of incubation in male embryos and around day 6.5 in female

embryos [23], with morphological differentiation in the gonads becoming apparent only after day 6.5. These developmental constraints suggest that pronounced sex-specific features are unlikely to be present at earlier stages. Nevertheless, for this study, we hypothesized the following: subtle sex-related differences in vascular patterns may emerge prior to genetic expression, potentially enabling sex classification through blood vessels image analysis.

We recently developed an improved egg imaging system using laser speckle contrast imaging (LSCI) [21,22]. Compared to traditional egg candling, this approach offers superior detection of active blood flow in blood vessels and is robust against eggshell coloration variations and stains [21]. Blood vessel images acquired with our LSCI system would serve as better input for DNN-based image analysis. This study aims to verify the following hypothesis: LSCI-derived vascular patterns contain sufficient discriminative information for reliable sex determination at early developmental stages."

References:

16. Jia N, Li B, Zhao Y, Fan S, Zhu J, Wang H, et al. Exploratory Study of Sex Identification for Chicken Embryos Based on Blood Vessel Images and Deep Learning. Agriculture. 2023;13: 1480. doi:10.3390/agriculture13081480

17. Zhihui Z, Zifan Y, Yuting H, Kai Y, Qiaohua W, Meihu M. Gender identification of early chicken embryo based on RF-DS information fusion of spectroscopy and machine vision. Transactions of the Chinese Society of Agricultural Engineering (Transactions of the CSAE). 2022;38: 308–315. doi:10.11975/j.issn.1002-6819.2022.18.034

18. Zhihui Z, Yong T, Qi H, Piao H, Qiaohua W, Meihu M. Female and male identification of early chicken embryo based on blood line features of hatching egg image and deep belief networks. Transactions of the Chinese Society of Agricultural Engineering (Transactions of the CSAE). 2018;34: 197–203. doi:10.11975/j.issn.1002-6819.2018.06.025

19. Cheng B, Shing Ching CT, Van Hieu N, Tang PC, Huy HT, Nhan NC, et al. Application of Machine Learning to Impedance Data for In-Ovo Chicken Egg Sexing. IEEJ Transactions Elec Engng. 2025;20: 1927–1932. doi:10.1002/tee.70125

20. England AD, Kheravii SK, Musigwa S, Kumar A, Daneshmand A, Sharma NK, et al. Sexing chickens (Gallus gallus domesticus) with high-resolution melting analysis using feather crude DNA. Poultry Science. 2021;100: 100924. doi:10.1016/j.psj.2020.12.022

21. Dong Z, Mahler S, Readhead C, Chen X, Dickson M, Bronner M, et al. Non-invasive laser speckle contrast imaging (LSCI) of extra-embryonic blood vessels in intact avian eggs at early developmental stages. Biomed Opt Express. 2024;15: 4605. doi:10.1364/BOE.530366

22. Readhead C, Mahler S, Dong Z, Sato Y, Yang C, Bronner ME. Automated non-invasive laser speckle imaging of the chick heart rate and extraembryonic blood vessels and their response to Nifedipine and Amlodipine drugs. Developmental Biology. 2025;519: 46–54. doi:10.1016/j.ydbio.2024.12.005

23. Luo X, Guo J, Zhang J, Ma Z, Li H. Overview of chicken embryo genes related to sex differentiation. PeerJ. 2024;12: e17072. doi:10.7717/peerj.17072

Comment R4-2: *"2. Comparison with prior study is incomplete [e.g.: strongly contrasts its results with Ref. [13] (~85% accuracy) but does not investigate why."*

We agree that, at present, there is no clear or satisfactory explanation for the discrepancy in accuracy between our results and those reported in Ref. [13] in the previously submitted manuscript (now Ref. [16] in revised manuscript), despite the apparent similarity in experimental protocol and data analysis. We made efforts to better understand this discrepancy by requesting access to the dataset and implementation details from the authors of Ref. [16]. Unfortunately, we

did not receive a response, which limits our ability to perform a direct comparison or identify the underlying causes of the performance difference.

In this context, we believe that the value of our study is important in providing an independent benchmark using similar experimental framework. Our results contribute to a more balanced assessment of the feasibility of early sex classification based solely on vascular patterns. The relatively modest performance of our best-performing model (ResNetBit, 61% accuracy) supports the main conclusion of our study that sex classification of chicken embryos from LSCI-derived vascular images using current deep neural network (DNN) approaches remains inconclusive at this stage. As such, our study highlights the need for caution when interpreting the results of Ref. [16] and underscores the importance of reproducibility and independent validation in this area.

We added a Discussion section to interpret the limitations of the deep neural network (DNN) performance, the implications of the modest classification accuracy, and a comparison with the previously reported study. We also discuss potential reasons for the observed discrepancies, as well as directions for future research

"Discussions

In this study, we evaluated the feasibility of using deep neural networks (DNNs) to classify the sex of chicken embryos from laser speckle contrast imaging (LSCI)-derived vascular images. Despite employing multiple established DNN architectures with transfer learning and fine-tuning, the overall classification performance remained modest, with the best-performing model achieving an accuracy of 61%. These results indicate that, under the current experimental and analytical conditions, sex classification at early-stages from vascular patterns alone is marginally above chance and not reliable. Importantly, these findings are consistent with the central conclusion of this work: sex-specific differences in vascular structure at the investigated developmental stages are either too subtle to be captured by LSCI or not present at all.

A key motivation for this paper was to contrast our findings with prior work (Ref. (13)), which reported classification accuracies exceeding 85% using similar vascular images (egg candling). Despite using a comparable methodological framework, we were unable to reproduce such high performance. The reasons for this discrepancy remain unclear. Notably, the dataset used in that study is not publicly available, and attempts to obtain additional details were unsuccessful, preventing direct comparison and reproducibility assessment."

"Future studies could explore alternative approaches, such as multiple-instance learning to assess spatial and temporal variations in blood vessel patterns, feature extraction from LSCI images, blood flow dynamics and heart rate analysis, which would require precise temperature control during image acquisition for accurate measurements. These refinements may offer new insights into the potential for early-stage sex differentiation in chick embryos using LSCI imaging."

Comment R4-3: *"3. Statistical analysis needs improvement (e.g.: The use of binomial p-values for model accuracy)."*

We clarified that the reported p-values were calculated across cross-validation folds and modified the wording "fold-wise p-value" to "across cross-validation folds" to improve clarity.

We have also expanded the p-value explanation in the ResNeBit section to explicitly describe how these values were computed: "To assess whether the classification performance exceeded chance level, p-values were calculated across cross-validation folds (Table 2). For each

fold, the p-value was computed using a binomial test under the null hypothesis of random guessing, implemented via a binomial calculator [65]. Specifically, the observed classification accuracy of the deep neural network (DNN) for each fold was compared against the expected accuracy from random classification of male versus female egg embryo LSCI images (i.e., a chance level of 50%). The significance levels α are established as follows: $p > 0.05$ (ns: not significant), $p < 0.05$ (*), $p < 0.01$ (**), $p < 0.001$ (***), and $p < 0.0001$ (****)."

The following text was updated in the Abstract: "Specifically, ResNetBiT had a five-fold cross-validated average accuracy of 56% ± 4% (p-value $p < 0.28$ across cross-validation folds) at day 3 and 57% ± 3% (p-value $p < 0.07$ across cross-validation folds) at day 4. YOLOv5s-cls had a five-fold cross-validated average accuracy of 55%±2% ($p < 0.13$ across folds) at day 3 and 57% ± 3% ($p < 0.10$ across folds) at day 4."

The following text was updated in the Introduction: "ResNetBiT achieved an average accuracy of 56% at day 3 (p-value $p < 0.28$ across cross-validation folds) and 57% at day 4 ($p < 0.07$ across folds). YOLOv5s-cls achieved an average accuracy of 55% at day 3 ($p < 0.13$ across folds) and 57% at day 4 ($p < 0.10$ across folds)."

Comment R4-4: *"4. PLOS ONE data policy requires open data. Deposit dataset in a public repository (Zenodo / Dryad / Figshare)."*

We have created a public repository containing the dataset and code used to generate the findings reported in this manuscript. The repository has been deposited in Zenodo, which is one of the repositories recommended by the reviewer and PLOS ONE for data sharing. The repository can be accessed at the following link:

https://zenodo.org/records/19004322?token=eyJhbGciOiJIUzUxMiJ9.eyJpZCI6IjYwZTdiMGVjLTMyOWEtNDY5MS05YTVjLWQ0OTU1ZDQzNmY0OCIsImRhdGEiOnt9LCJyYW5kb20iOiIzZjQwNzlhZDY5NTJjNTdiNzY4NTljNGQ5Y2M1ZTBkZCJ9.tIFalcb3V9aCwCLno5uCitBhZFBinbXC6pnOZzHFka6EPekYvE56muUQfPyiaNRORSbfIW3Q3CG23RxTZkc0nQ

This dataset represents the minimal anonymized data required to replicate the results presented in the study.

Comment R4-5: *"5. Limited exploration of alternative signals: The paper examines only static vascular morphology. LSCI measures blood flow dynamics, so authors should evaluate temporal features, not only spatial patterns."*

We agree that the advantage of LSCI lies in capturing hemodynamic and perfusion-related information rather than static structural features. Accordingly, we did explore this direction by analyzing sex-related differences in embryonic heart rate and temporal blood flow graphs at days 3 and 4 of incubation. However, we did not observe any statistically significant differences between male and female embryos at these early stages. One major challenge was the high inter-egg variability in heart rate, which is particularly pronounced at those early stages of development. Indeed, at days 3 and 4 of incubation, the embryonic heart is still immature and has not yet fully developed into a well-structured four-chamber organ with a stable and regular beating pattern. As a result, variations due to differences in developmental stage appear to be larger than any potential differences associated with sex.

Another important limitation is the strong dependence of heart rate on temperature. We observed that even small temperature variations (as little as ± 1 °C) can lead to non-negligible changes in heart rate and blood flow dynamics. During imaging, eggs have to be removed from the

incubator, requiring data acquisition to be completed within approximately 3 minutes to minimize temperature drop effects. To address this limitation, we are working on integrating an incubator directly into the LSCI system (Ref. [22]).

For completeness, we extended this analysis to a later stage (> day 6), where the heart is more developed, and recorded data from 39 eggs. We maintained the temperature of the eggs to be always above 37 °C for this experiment. Ground-truth sexing was done similarly than in the manuscript (PCR test + gonads visualization).

The results (Table R1 and Figure R2) still did not reveal clear or reliable sex-specific differences in heart rate. These findings suggest that, while hemodynamic features are a promising direction in principle, heart rate alone may not provide a sufficiently robust signal for early sex determination, particularly at very early developmental stages, which is why we focused on the blood vessels images.

Table R1: List of day-6 incubation chicken eggs for which blood flow was measured and heart rate was extracted for sex determination.

Egg ID	T egg (°C)	Heart rate (bpm)	Visual+PCR Sex
1410	> 37	190	F
1419	> 37	228	F
1421	> 37	232	F
1425	> 37	228	F
1428	> 37	228	F
1451	> 37	215	F
1462	37.6	215	F
1465	37.9	235	F
1466	37.8	235	F
1471	37.6	230	F
1472	38.0	205	F
1474	37.6	190	F
1476	37.5	215	F
1477	37.8	205	F
1478	37.0	215	F
1484	37.0	215	F
1485	37.3	225	F
1486	37.3	215	F
1488	37.6	220	F
1489	37.6	220	F
1411	> 37	215	M
1418	> 37	215	M

1420	> 37	241	M
1424	> 37	119	M
1450	> 37	241	M
1455	> 37	228	M
1463	38.2	225	M
1464	38.0	230	M
1468	37.2	235	M
1469	37.4	245	M
1470	36.9	225	M
1475	37.2	190	M
1476	38.0	177	M
1479	37.2	150	M
1480	37.5	220	M
1481	37.5	225	M
1482	37.1	155	M
1483	37.4	230	M
1490	37.6	185	M

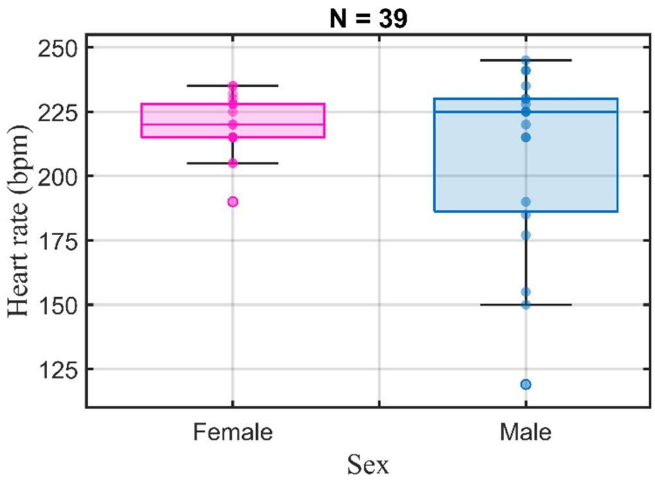

bpm	Female	Male
Average	218	208
Variance	13	35
Quartile Q1	215	188
Q2 (median)	218	225
Q3	228	230
Q4	235	245

Figure S7 (left) Statistical distribution of heart rate for male and female chicken embryos at day 6 of incubation. Each dot in the graph represents a chicken egg. (Right) Summary table showing mean, median, and quartiles of heart rate for each sex. As shown, there is no clear statistical difference between female and male in heart rate, even though the male distribution appears more dispersed.

We have added a paragraph in the Discussion section addressing the feasibility of sexing chicken eggs based on blood flow dynamics and heart rate at days 3–4 of incubation: "LSCI is particularly well suited for capturing hemodynamic and perfusion-related information (blood flow time trace). Exploration of temporal features, including embryonic heart rate (HR) and blood flow dynamics, did not reveal reliable sex-specific differences at early (days 3–4) developmental stages

(see Supporting Information). At these stages, the embryonic heart remains immature and has not yet developed into a fully structured four-chamber organ with a stable and regular beating pattern. Consequently, heart rate measurements exhibited inter-embryo variability, driven by differences in developmental stage rather than sex. In addition, heart rate measurements are sensitive to temperature fluctuations, where variations as small as ±1 °C were found to affect the measurements.

Finally, analysis at a later stage (day 6), where cardiac development is more advanced was conducted on 39 eggs (20 female and 19 male). The temperature of the eggs was always maintained above 37 °C. The results, shown in Supporting Figure S7, did not reveal clear sex-specific differences in heart rate between female and male, however, a larger dataset would be required to draw more definitive conclusions."

Supporting Figure S7 was also added to the Supporting Information.

Comment R4-6: *"6. The discussion should emphasize - biological limitations likely explain failure rather than model limitations."*

Per the reviewer's comment, we have revised the conclusion to emphasize more the biological limitation likely explained failure of our DNN analysis:

"Conclusion

Using laser speckle contrast imaging (LSCI), images of chick embryo blood vessels at days 3 and 4 of incubation were recorded to explore the feasibility of sex differentiation using deep neural network (DNN) models. Our final dataset consisted of images from 1,251 chick embryos, providing a substantial sample size for a pilot DNN testing. Two DNN models were trained: ResNetBiT and YOLOv5, both achieved not satisfactory accuracy and relatively high BCE loss, with YOLOv5s-cls performing worse than ResNetBiT. A per-egg evaluation approach did not provide high accuracy or compelling statistical significance, leading us to conclude that sex identification from LSCI images at this early incubation stage is likely not feasible for practical applications.

Importantly, these negative results are unlikely to stem primarily from model limitations. Instead, they are more plausibly explained by fundamental biological constraints at this early developmental stage. Previous literature on genetic expression related to sex differentiation in embryos indicates that most genes influencing sex determination begin to express around day 4.5 of incubation in male embryos and day 6.5 in female embryos [23]. From day 6.5 onward, gradual morphological changes in gonadal tissue become observable, marking the onset of sex organ development. At days 3–4, when our imaging was performed, sex-specific physiological or vascular signatures are therefore expected to be minimal or absent, limiting the availability of discriminative features in blood vessel images.

Additionally, the chorioallantoic membrane (CAM)—a highly vascularized extra-embryonic membrane—plays a crucial role in embryonic development, supporting vital functions such as gas exchange, calcium absorption, and waste removal. Since the CAM begins to form around day 5 of incubation (stage HH25) and is closely linked to the embryo's genetic identity and overall development, we hypothesize that it may also be involved in sex differentiation. Given that the CAM is rich in active blood vessels and continues

developing until days 12–14, this could explain why sex identification through blood vessel imaging may become more viable at later incubation stages.

While we hypothesize that sex differentiation may be detectable from day 6 onward, our current LSCI system is limited to imaging up to day 5.5 due to increased embryonic motion, which degrades speckle contrast measurements during multi-frame acquisition (total acquisition time of 5 s for 100 frames at 20 FPS, 10 ms exposure). To address this limitation, we plan to upgrade to a higher-speed camera (90 FPS), reducing the inter-frame interval to 11 ms and the total acquisition time to approximately 1.1 s, minimizing motion-induced artifacts. This improvement is expected to enable reliable LSCI imaging at day 6 and beyond."

Comment R4-7: *"7. Overfitting concerns: Training accuracy approached 75–100% while validation accuracy hovered near random chance (50–61%).*

We thank the reviewer for this concern. Our original experiments already included several strategies to mitigate overfitting: systematic data augmentation ablations (rotation up to ±180°, translation, scaling, and random flips) that progressively reduced the train–validation gap (Figure S4), cosine annealing learning rate scheduling, transfer learning from pretrained weights, and early stopping. In response to this feedback, we further strengthened regularization for both models. For ResNetBiT, we froze all backbone layers except the last two stages, added dropout (p = 0.3) and weight decay (1e-4), and switched to ACC- and loss-based early stopping from epoch 11. For YOLOv5s-cls, we froze backbone layers 0–9, added dropout (p = 0.1), and enabled on-the-fly augmentation with early stopping (patience = 15, with a 20 epoch warm up). These changes substantially reduced the train–validation gap (updated Figures 4 and 5). However, the model fits the training eggs (training loss decreases), but fails to generalize to unseen eggs (validation diverges). This supports the conclusion that the existing signal is not robust enough to generalize for early sex identification in chicken embryos.

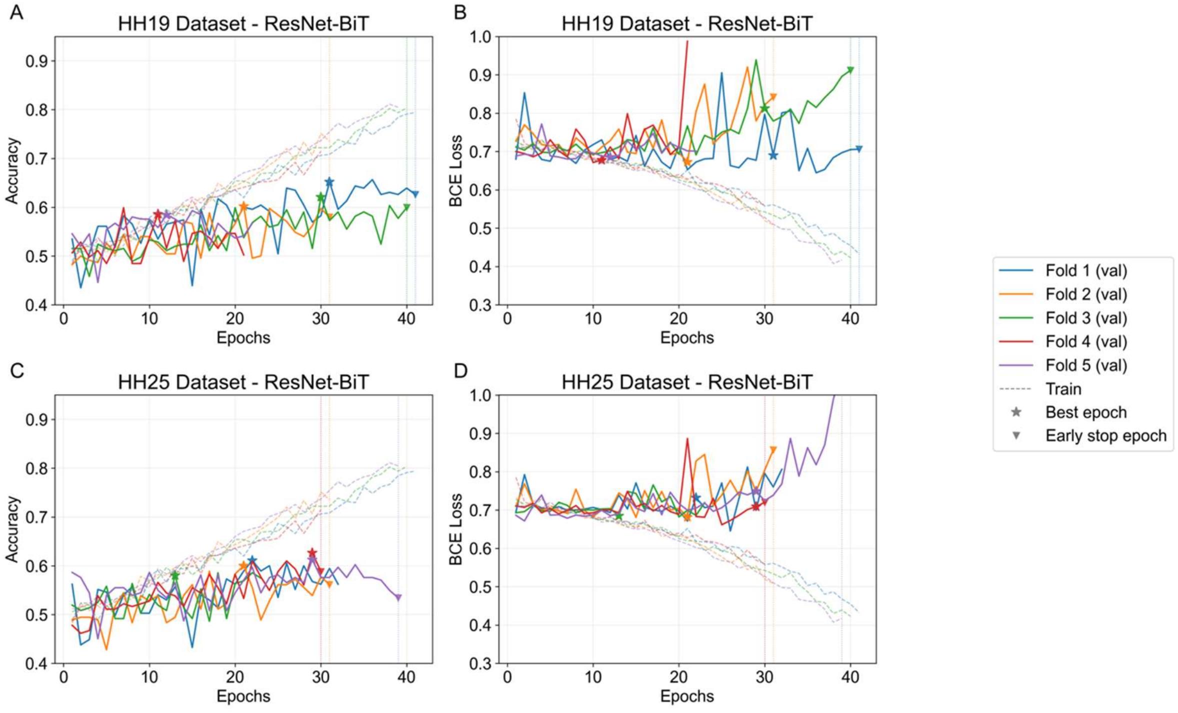

Fig 4 Sex identification results on LSCI images using the ResNetBiT model. (A) Accuracy and (B) BCE loss as function of the number of epochs for each fold of the training (dotted line) and validation (plain line) datasets on day 3 of incubation. Each fold is represented by a different line color. (C) Accuracy and (D) BCE loss on day 4.

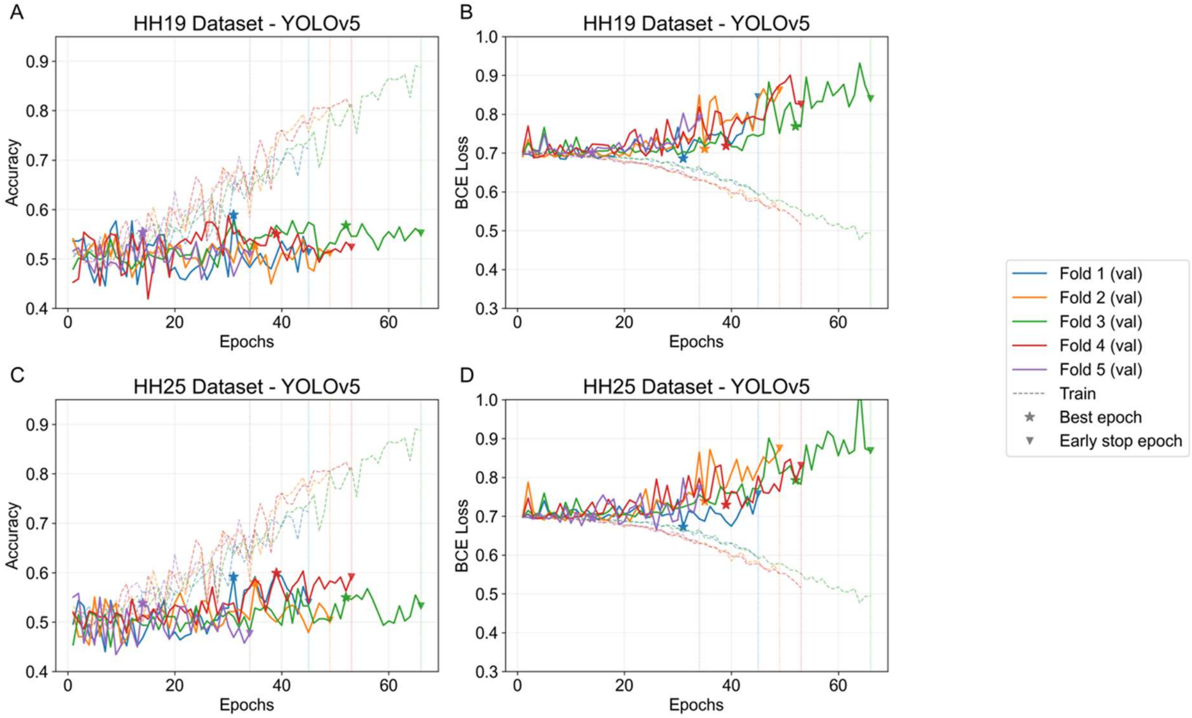

Fig 5 Sex identification results on LSCI images using the YOLOv5s-cls model. (A) Accuracy and (B) BCE loss as function of the number of epochs for each fold of the training (dotted line) and validation (plain line) datasets on day 3 of incubation. Each fold is represented by a different line color. (C) Accuracy and (D) BCE loss on day 4

Regarding overfitting with the ResNetBit model, please refer to Comment R3-2 where we added a Fig. S4 and more details on how overfitting was mitigated.

Revisions:

We have significantly revised the writing to detail more about the adopted strategies to mitigate overfitting and have updated the results of the re-ran YOLOv5s-cls experiment where overfitting is now less severe. Specifically:

We have revised the “Approaches: ResNetBit and YOLOv5” section of the Materials and methods to better clarify overfitting concerns:

“For both ResNetBit and YOLOv5s-cls models, several regularization strategies were applied exclusively to the training set to improve model generalization and prevent overfitting (see Supporting Information Fig S4). Training-only data augmentation included included random rotations up to 180°, translations up to 5% in any direction, image scaling between 0.85x and 1.15x, random vertical and horizontal flips with probability 0.3, and brightness/contrast/saturation jitter. No augmentations were applied to the validation set. Five-fold cross-validation with egg-ID-based splits were implemented to prevent data leakage and evaluate the model's performance.

For ResNetBiT, all backbone layers were frozen except the last two stages, dropout (p = 0.3) and weight decay (1e-4) were added, and the learning rate was set to 5e-5 with a 5-epoch linear warmup followed by cosine annealing. Early stopping was applied from epoch 11 onward using

two criteria: validation ACC (patience = 10, minimum improvement = 0.01) and validation loss (patience = 5), halting training when either criterion was triggered.

For YOLOv5s-cls, backbone layers 0–9 were frozen, dropout (p = 0.1) was added to the classification head, and weight decay (5×10^{-5}) was applied. Early stopping with a patience of 15 epochs was applied after a 20-epoch warmup. This strategy ensured that training was stopped before training and validation performance significantly diverged (Fig. S4)."

In the section Approaches: ResNetBiT and YOLOv5s-cls:

"Both models were trained using the Adam optimizer, BCE loss function, and cosine annealing learning rate scheduler to ensure stable convergence. To prevent overfitting, several regularization strategies were employed. For ResNetBiT, we froze all backbone layers except the last two stages, added dropout (p = 0.3) and weight decay (1e-4), and used a learning rate of 5e-5 with a 5-epoch linear warmup followed by cosine annealing. Early stopping was applied from epoch 11 onward using two criteria: validation ACC (patience = 10, minimum improvement = 0.01) and validation loss (patience = 5). For YOLOv5s-cls, we froze backbone layers 0–9, added dropout (p = 0.1), and applied weight decay (5×10^{-5}) with a cosine annealing learning rate scheduler. Early stopping with a patience of 15 epochs was applied after a 20-epoch warmup. Both models used training-only data augmentation including rotations (up to ±180°), translations, scaling, random flips, and brightness/contrast jitter, as well as transfer learning from ImageNet pretrained weights. Five-fold cross-validation with egg-ID-based splits was used to prevent data leakage and ensure reproducibility."

In the first paragraph of the ResNetBiT section:

"The five-fold cross-validation results for the ResNetBiT model are presented in Fig 4 and Table 2. Figures 4A and 4C depict the training and validation accuracy curves over the course of epoch for day 3 and day 4. Early stopping was applied from epoch 11 onward using two criteria: validation ACC (patience = 10, minimum improvement = 0.01) and validation loss (patience = 5)."

In the first paragraph of the YOLOv5s-cls section:

"The five-fold cross-validated results for the YOLOv5s-cls model are presented in Fig 5 and Table 3. Figures 5A and 5C depict the training and validation accuracy curves over the course of epochs for day 3 and day 4. Figures 5B and 5D show the BCE loss. To mitigate overfitting, the following strategies were adopted and described in Data Preparation and Preprocessing section of the Materials and methods to better clarify overfitting concerns."

We added a short conclusion at the end of the YOLOv5s-cls Results section:

"Despite early-stopping and other strategies preventing overfitting such as backbone freezing, dropout, weight decay, cosine annealing, and extensive data augmentation (see Materials and methods), as shown in Fig. 5, the validation accuracy did not improve meaningfully across training epochs, remaining close to chance level. While the validation performance remained near chance, the training accuracy increased, indicating that while overfitting was somehow controlled, reliable generalization was not achieved. This suggests that LSCI blood vessels images lack sufficiently robust sex-discriminative features for models such as YOLOv5 to learn from, and therefore the training process did not yield a valid model for hypothesis testing purposes."

We also have updated Figs. 4 and 5, Tables 2 and 3. We also added a Figure S4 to the Supporting Information.

Comment R4-8: *"Minor Comments:*
1. Several grammartical issues and typographical errors.

The manuscript has been carefully proofread, and grammatical and typographical errors have been corrected throughout.

"2. Some figure references are inconsistent."

We have reviewed the manuscript and corrected figure references (including those in the Supporting Information), to ensure consistency throughout the manuscript. Specifically, incorrect references in line 349 and line 355 have been corrected to Fig 5A and Fig 5B, respectively. In accordance with PLOS ONE formatting guidelines, all figures are now labeled as "Fig XX" in the text (with "Figure XX" used only at the beginning of a sentence).

"3. The preprocessing figure could be clearer."

In accordance with the PLOS ONE, we have created a Supporting Information and moved all Appendix material to the Supporting Information. The preprocessing figures have been moved to the Supporting Information (Figures S2 and S3) and are provided as a separate PDF. This change improves their resolution and overall clarity. We also have ensured higher resolution of resubmitted manuscript.

4. Some citations are outdated regarding chick sexing technologies.

We have reviewed the recent literature on chick sexing technologies and updated the manuscript accordingly. Specifically, we added recent publications related to the use of optical methods and detection algorithms in the introduction. We note that the original submission was in April 2025; therefore, most recent publications were not included at that time. We have now incorporated relevant recent studies to ensure the manuscript reflects the current state of the field.

"5. Table numbering could be improved."

In the revised manuscript, tables are numbered sequentially in the order of their first citation in the text. In accordance with the PLOS ONE format guidelines, each table now includes a title above it and is explicitly referenced in the manuscript.

Pre-production comment:

"6. PLOS authors have the option to publish the peer review history of their article (what does this mean?). If published, this will include your full peer review and any attached files."

We would like to publish the peer review history of this article please. Thank you for this transparency opportunity.

In view of the above, we hope that you will favorably consider this manuscript and look forward to hearing from you very soon.

Sincerely yours,
Dr. Simon Mahler,
on behalf of all the authors.
