# Supplementary material for: Detection of non-invasive sexing of early chick embryos in intact eggs using laser speckle contrast imaging and deep neural networks
Source: PLoS One. 2026 Jun 26;21(6):e0323847. doi: 10.1371/journal.pone.0323847 (PMC13308874; doi:10.1371/journal.pone.0323847)
Supplement: Attachment — Submitted filename: Response_to_Reviewers_auresp_2.pdf [file pone.0323847.s010.pdf]

Manuscript ID: PONE-D-25-20406R1

Title: Detection of non-invasive sexing of early chick embryos in intact eggs using laser speckle contrast imaging and deep neural networks

Authors: S. Mahler et al; California Institute of Technology, Stevens Institute of Technology.

Subject: Revised manuscript + response letter to reviewer's comments

Dear Khan Bahadar Khan,

We have received your decision on May-14, 2026 to give us an opportunity to revise our manuscript. Below, we address the reviewers' comments, editorial comments and instructions, together with our changes made to the manuscript. We appreciate your consideration and look forward to receiving your final decision soon.

We also attach to this resubmission a marked-up copy of the revised manuscript (labeled "Revised Manuscript with Track Changes") and an unmarked version without tracked changes (labeled "Manuscript").

We do not wish to make any change to the financial disclosure.

Editorial Comments:

"If applicable, we recommend that you deposit your laboratory protocols in protocols.io to enhance the reproducibility of your results. Protocols.io assigns your protocol its own identifier (DOI) so that it can be cited independently in the future. For instructions see: https://journals.plos.org/plosone/s/submission-guidelines#loc-laboratory-protocols. Additionally, PLOS ONE offers an option for publishing peer-reviewed Lab Protocol articles, which describe protocols hosted on protocols.io. Read more information on sharing protocols at

https://plos.org/protocols?utm_medium=editorialemail&utm_source=authorletters&utm_campaign=protocols."

Thank you for the recommendation. All laboratory protocols and methodological details are already fully described within the manuscript and supplementary materials to support reproducibility. Therefore, we do not wish to submit the protocols separately to protocols.io or as a Lab Protocol article at this time.

"As the corresponding author, your ORCID iD is verified in the submission system and will appear in the published article.

PLOS supports the use of ORCID, and we encourage all coauthors to register for an ORCID iD and use it as well. Please

encourage your coauthors to verify their ORCID iD within the submission system before final acceptance, as unverified

ORCID iDs will not appear in the published article. Only the individual author can complete the verification step; PLOS staff

cannot verify ORCID iDs on behalf of authors."

I have notified co-authors about linking their ORCID iD within the submission system.

"If the reviewer comments include a recommendation to cite specific previously published works, please review and evaluate these publications to determine whether they are relevant and should be cited. There is no requirement to cite these works unless the editor has indicated otherwise.

Please review your reference list to ensure that it is complete and correct. If you have cited papers that have been retracted, please include the rationale for doing so in the manuscript text, or remove these references and replace them with relevant current references. Any changes to the reference list should be mentioned in the rebuttal letter that accompanies your revised manuscript. If you need to cite a retracted article, indicate the article's retracted status in the References list and also include a citation and full reference for the retraction notice."

Thank you for this information and take note of it. I reviewed the reference list and did not see any obvious or widely known retracted articles.

All Reviewers:

General Evaluation: *"Comments to the Author*

1. If the authors have adequately addressed your comments raised in a previous round of review and you feel that this manuscript is now acceptable for publication, you may indicate that here to bypass the "Comments to the Author" section, enter your conflict of interest statement in the "Confidential to Editor" section, and submit your "Accept" recommendation.
Reviewer #1: All comments have been addressed
Reviewer #2: All comments have been addressed
Reviewer #3: All comments have been addressed
Reviewer #4: All comments have been addressed

2. Is the manuscript technically sound, and do the data support the conclusions? The manuscript must describe a technically sound piece of scientific research with data that supports the conclusions. Experiments must have been conducted rigorously, with appropriate controls, replication, and sample sizes. The conclusions must be drawn appropriately based on the data presented.
Reviewer #1: Yes
Reviewer #2: Yes
Reviewer #3: Yes
Reviewer #4: Yes

3. Has the statistical analysis been performed appropriately and rigorously?
Reviewer #1: Yes
Reviewer #2: Yes
Reviewer #3: Yes
Reviewer #4: Yes

4. Have the authors made all data underlying the findings in their manuscript fully available? The PLOS Data policy requires authors to make all data underlying the findings described in their manuscript fully available without restriction, with rare exception (please refer to the Data Availability Statement in the manuscript PDF file). The data should be provided as part of the manuscript or its supporting information, or deposited to a public repository. For example, in addition to summary statistics, the data points behind means, medians and variance measures

should be available. If there are restrictions on publicly sharing data—e.g. participant privacy or use of data from a third party—those must be specified.
Reviewer #1: Yes
Reviewer #2: Yes
Reviewer #3: Yes
Reviewer #4: Yes

5. Is the manuscript presented in an intelligible fashion and written in standard English?
PLOS ONE does not copyedit accepted manuscripts, so the language in submitted articles must be clear, correct, and unambiguous. Any typographical or grammatical errors should be corrected at revision, so please note any specific errors here.
Reviewer #1: Yes
Reviewer #2: Yes
Reviewer #3: Yes
Reviewer #4: Yes
"

We are pleased that all reviewers have found that the comments have been fully addressed, and that the manuscript is now acceptable for publication.

Reviewer 1:

"Reviewer #1: This article has relatively high novelty regarding sex detection in eggs, which is a topic that many researchers actively investigate. However, the presentation and several related details require greater attention. I believe this article has undergone revision for a very long time, and I am not sure what caused the delay."

Comment R1-1: *"Despite the many improvements, several aspects still require clarification. The following are my minor revisions:*
1. Please provide the dataset link through a cloud-based repository that supports DOI assignment or use a national scientific data repository facility from an institutional source, such as a national data repository. Please consider this carefully."

We resubmitted the dataset to a new DOI-assigned public repository. The dataset was deposited in Zenodo, a recognized open-access repository that provides persistent identifiers and supports long-term archival and accessibility. Zenodo is listed among the recommended repositories by PLOS ONE and satisfy the reviewer's requirement for a dedicated DOI-assigned archival platform. This update ensures compliance with long-term data-sharing principles and transparency. The dataset is publicly available at this link with the corresponding DOI: 10.5281/zenodo.20316359.

Revisions: In the *Data availability* section:

"The data that support the findings of this study are publicly available from the following Zenodo repository dataset using this link with the corresponding DOI: 10.5281/zenodo.20316359.

~~Dataset URL:~~

~~https://zenodo.org/records/19004322?token=eyJhbGciOiJIUzUxMiJ9.eyJpZCI6IjYwZTdiMGVjLTMyOWEtNDY5MS05YTVjLWQ0OTU1ZDQzNmY0OCIsImRhdGEiOnt9LCJyYW5kb20iOiIzZjQwNzlhZDY5NTJjNTdiNzY4NTljNGQ5Y2M1ZTBkZCJ9.tIFalcb3V9aCwCLno5uCitBhZFBinbXC6pnOZzHFka6EPekYvE56muUQfPyiaNRORSbfIW3Q3CG23RxTZkc0nQ~~"

Comment R1-2: *"2. The URL for the GitHub repository can be included; however, it may be better to keep this available only upon request from the corresponding author. Other than that, I have no further comments."*

Per suggestion of the reviewer, we removed the GitHub repository link from the manuscript. The algorithms scripts are now stated as available from the corresponding author upon reasonable request.

Revisions in the *Data availability* section:

~~"Code URL: https://github.com/anika24/lsci-egg-sex-classification~~ The scripts and codes used for the deep neural network analyses in this study are available from the corresponding author upon reasonable request."

Reviewer 2:

"Reviewer #2: The authors have addressed all the comments from the reviewers. This manuscript is now scientifically sound, meticulously documented, and ethically transparent. It fully meets the publication criteria for PLOS ONE. I recommend accepting the manuscript in its current form without the need for further revisions."

Reviewer 3:

Reviewer #3: (No Response)

Reviewer 4:

"Reviewer #4: Substantial revisions have been made to the manuscript including to comply with the formatting and style requirements and to report statistical significance."

In view of the above, we hope that you will favorably consider this manuscript and look forward to hearing from you very soon.

Sincerely yours,
Dr. Simon Mahler,
on behalf of all the authors.
