# Supplementary material for: Detection of non-invasive sexing of early chick embryos in intact eggs using laser speckle contrast imaging and deep neural networks
Source: PLoS One. 2026 Jun 26;21(6):e0323847. doi: 10.1371/journal.pone.0323847 (PMC13308874; doi:10.1371/journal.pone.0323847)
Supplement: Peer review history

# Peer review: Decision Letter 0

**DOI:** 10.1371/journal.pone.0323847.r001

**Authors:** Khan Bahadar Khan

11 Mar 2026

PONE-D-25-20406

Exploring non-invasive sexing of early chick embryos in intact eggs using Laser Speckle Contrast Imaging (LSCI) and Deep Neural Network (DNN)

PLOS One

Dear Dr. Mahler,

Thank you for submitting your manuscript to PLOS ONE. After careful consideration, we feel that it has merit but does not fully meet PLOS ONE’s publication criteria as it currently stands. Therefore, we invite you to submit a revised version of the manuscript that addresses the points raised during the review process.

Please submit your revised manuscript by Apr 25 2026 11:59PM. If you will need more time than this to complete your revisions, please reply to this message or contact the journal office at plosone@plos.org. When you're ready to submit your revision, log on to https://www.editorialmanager.com/pone/ and select the 'Submissions Needing Revision' folder to locate your manuscript file.

Please include the following items when submitting your revised manuscript:

- A letter that responds to each point raised by the academic editor and reviewer(s). You should upload this letter as a separate file labeled 'Response to Reviewers'.
- A marked-up copy of your manuscript that highlights changes made to the original version. You should upload this as a separate file labeled 'Revised Manuscript with Track Changes'.
- An unmarked version of your revised paper without tracked changes. You should upload this as a separate file labeled 'Manuscript'.

If you would like to make changes to your financial disclosure, please include your updated statement in your cover letter. Guidelines for resubmitting your figure files are available below the reviewer comments at the end of this letter.

If applicable, we recommend that you deposit your laboratory protocols in protocols.io to enhance the reproducibility of your results. Protocols.io assigns your protocol its own identifier (DOI) so that it can be cited independently in the future. For instructions see: https://journals.plos.org/plosone/s/submission-guidelines#loc-laboratory-protocols. Additionally, PLOS ONE offers an option for publishing peer-reviewed Lab Protocol articles, which describe protocols hosted on protocols.io. Read more information on sharing protocols at https://plos.org/protocols?utm_medium=editorial-email&utm_source=authorletters&utm_campaign=protocols.

We look forward to receiving your revised manuscript.

Kind regards,

Khan Bahadar Khan, Ph.D

Academic Editor

PLOS One

Journal Requirements:

When submitting your revision, we need you to address these additional requirements.

1. Please ensure that your manuscript meets PLOS ONE's style requirements, including those for file naming. The PLOS ONE style templates can be found at

https://journals.plos.org/plosone/s/file?id=wjVg/PLOSOne_formatting_sample_main_body.pdf and

https://journals.plos.org/plosone/s/file?id=ba62/PLOSOne_formatting_sample_title_authors_affiliations.pdf

2. Please note that PLOS ONE has specific guidelines on code sharing for submissions in which author-generated code underpins the findings in the manuscript. In these cases, we expect all author-generated code to be made available without restrictions upon publication of the work.

Please review our guidelines at https://journals.plos.org/plosone/s/materials-and-software-sharing#loc-sharing-code and ensure that your code is shared in a way that follows best practice and facilitates reproducibility and reuse.

3. We note that the grant information you provided in the ‘Funding Information’ and ‘Financial Disclosure’ sections do not match.

When you resubmit, please ensure that you provide the correct grant numbers for the awards you received for your study in the ‘Funding Information’ section.

4. Thank you for stating the following financial disclosure:

“IST Carver Mead Endowment (25550038)”

Please state what role the funders took in the study.  If the funders had no role, please state: "The funders had no role in study design, data collection and analysis, decision to publish, or preparation of the manuscript." If this statement is not correct you must amend it as needed.

Please include this amended Role of Funder statement in your cover letter; we will change the online submission form on your behalf.

5. Please note that funding information should not appear in the Acknowledgments section or other areas of your manuscript. We will only publish funding information present in the Funding Statement section of the online submission form. Please remove any funding-related text from the manuscript.

6. We note that you have indicated that there are restrictions to data sharing for this study. PLOS only allows data to be available upon request if there are legal or ethical restrictions on sharing data publicly. For more information on unacceptable data access restrictions, please see http://journals.plos.org/plosone/s/data-availability#loc-unacceptable-data-access-restrictions.

Before we proceed with your manuscript, please address the following prompts:

a) If there are ethical or legal restrictions on sharing a de-identified data set, please explain them in detail (e.g., data contain potentially identifying or sensitive patient information, data are owned by a third-party organization, etc.) and who has imposed them (e.g., a Research Ethics Committee or Institutional Review Board, etc.). Please also provide contact information for a data access committee, ethics committee, or other institutional body to which data requests may be sent.

b) If there are no restrictions, please upload the minimal anonymized data set necessary to replicate your study findings to a stable, public repository and provide us with the relevant URLs, DOIs, or accession numbers. For a list of recommended repositories, please see

https://journals.plos.org/plosone/s/recommended-repositories. You also have the option of uploading the data as Supporting Information files, but we would recommend depositing data directly to a data repository if possible.

We will update your Data Availability statement on your behalf to reflect the information you provide.

7. In this instance it seems there may be acceptable restrictions in place that prevent the public sharing of your minimal data. However, in line with our goal of ensuring long-term data availability to all interested researchers, PLOS’ Data Policy states that authors cannot be the sole named individuals responsible for ensuring data access (http://journals.plos.org/plosone/s/data-availability#loc-acceptable-data-sharing-methods).

Data requests to a non-author institutional point of contact, such as a data access or ethics committee, helps guarantee long term stability and availability of data. Providing interested researchers with a durable point of contact ensures data will be accessible even if an author changes email addresses, institutions, or becomes unavailable to answer requests.

Before we proceed with your manuscript, please also provide non-author contact information (phone/email/hyperlink) for a data access committee, ethics committee, or other institutional body to which data requests may be sent. If no institutional body is available to respond to requests for your minimal data, please consider if there any institutional representatives who did not collaborate in the study, and are not listed as authors on the manuscript, who would be able to hold the data and respond to external requests for data access? If so, please provide their contact information (i.e., email address). Please also provide details on how you will ensure persistent or long-term data storage and availability.

8. We note that Figures 1 and 2 in your submission contain copyrighted images. All PLOS content is published under the Creative Commons Attribution License (CC BY 4.0), which means that the manuscript, images, and Supporting Information files will be freely available online, and any third party is permitted to access, download, copy, distribute, and use these materials in any way, even commercially, with proper attribution. For more information, see our copyright guidelines: http://journals.plos.org/plosone/s/licenses-and-copyright.

We require you to either (1) present written permission from the copyright holder to publish these figures specifically under the CC BY 4.0 license, or (2) remove the figures from your submission:

1) You may seek permission from the original copyright holder of Figures 1 and 2 to publish the content specifically under the CC BY 4.0 license.

We recommend that you contact the original copyright holder with the Content Permission Form (http://journals.plos.org/plosone/s/file?id=7c09/content-permission-form.pdf) and the following text:

“I request permission for the open-access journal PLOS ONE to publish XXX under the Creative Commons Attribution License (CCAL) CC BY 4.0 (http://creativecommons.org/licenses/by/4.0/). Please be aware that this license allows unrestricted use and distribution, even commercially, by third parties. Please reply and provide explicit written permission to publish XXX under a CC BY license and complete the attached form.”

Please upload the completed Content Permission Form or other proof of granted permissions as an ""Other"" file with your submission.

In the figure caption of the copyrighted figure, please include the following text: “Reprinted from [ref] under a CC BY license, with permission from [name of publisher], original copyright [original copyright year].”

2) If you are unable to obtain permission from the original copyright holder to publish these figures under the CC BY 4.0 license or if the copyright holder’s requirements are incompatible with the CC BY 4.0 license, please either i) remove the figure or ii) supply a replacement figure that complies with the CC BY 4.0 license. Please check copyright information on all replacement figures and update the figure caption with source information.

If applicable, please specify in the figure caption text when a figure is similar but not identical to the original image and is therefore for illustrative purposes only.

9. If the reviewer comments include a recommendation to cite specific previously published works, please review and evaluate these publications to determine whether they are relevant and should be cited. There is no requirement to cite these works unless the editor has indicated otherwise.

[Note: HTML markup is below. Please do not edit.]

Reviewers' comments:

Reviewer's Responses to Questions

**Comments to the Author**

1. Is the manuscript technically sound, and do the data support the conclusions?

The manuscript must describe a technically sound piece of scientific research with data that supports the conclusions. Experiments must have been conducted rigorously, with appropriate controls, replication, and sample sizes. The conclusions must be drawn appropriately based on the data presented.

Reviewer #1: Yes

Reviewer #2: Yes

Reviewer #3: Partly

Reviewer #4: Partly

**********

2. Has the statistical analysis been performed appropriately and rigorously?

Reviewer #1: Yes

Reviewer #2: Yes

Reviewer #3: Yes

Reviewer #4: No

**********

3. Have the authors made all data underlying the findings in their manuscript fully available?

The PLOS Data policy requires authors to make all data underlying the findings described in their manuscript fully available without restriction, with rare exception (please refer to the Data Availability Statement in the manuscript PDF file). The data should be provided as part of the manuscript or its supporting information, or deposited to a public repository. For example, in addition to summary statistics, the data points behind means, medians and variance measures should be available. If there are restrictions on publicly sharing data—e.g. participant privacy or use of data from a third party—those must be specified.

Reviewer #1: Yes

Reviewer #2: Yes

Reviewer #3: Yes

Reviewer #4: No

**********

4. Is the manuscript presented in an intelligible fashion and written in standard English?

PLOS ONE does not copyedit accepted manuscripts, so the language in submitted articles must be clear, correct, and unambiguous. Any typographical or grammatical errors should be corrected at revision, so please note any specific errors here.

Reviewer #1: No

Reviewer #2: Yes

Reviewer #3: Yes

Reviewer #4: Yes

**********

5. Review Comments to the Author

Please use the space provided to explain your answers to the questions above. You may also include additional comments for the author, including concerns about dual publication, research ethics, or publication ethics. (Please upload your review as an attachment if it exceeds 20,000 characters)

Reviewer #1: 1. The use of the term “Exploring” in the title appears less appropriate. A more precise and assertive word, such as “Detection”, may better reflect the nature of the study.

2. First-person pronouns such as “we” should be avoided, in accordance with the conventions of scientific writing.

3. The phrase “fold-wise p-value, p < 0.3” is unclear. Please clarify the intended meaning and context of this expression.

4. Statistical significance should be reported using only the less than symbol (“<”), without the equal sign.

5. It was initially assumed that the Deep Neural Network (DNN) used in the study was developed independently. However, the model appears to rely on existing algorithms. Please clearly state which parts of the architecture or training process were modified or uniquely implemented in the Methods section.

6. The manuscript lacks a distinct discussion section. Including a well-structured discussion would greatly improve the clarity and scientific rigor of the paper.

The novelty of the research is not in question. However, the manuscript suffers from insufficient detail and overly simplistic explanations. These weaknesses reduce the overall impact of the study. Please revise the content thoroughly and align it with the standards of the journal.

Reviewer #2: The research methods and steps in this manuscript are generally accurate, and well-documented, aligning with standards for reproducibility and ethical conduct in developmental biology and imaging studies. Although the conclusions are negative, I believe this study is helpful for research and development in related fields. I have the following comments:

(1) After obtaining the laser speckle contrast image and BFI image, the authors only utilized the vascular structure information. Traditional methods, such as egg candling, provide more detailed vascular structure information, which may explain why the results of the tested method are inferior to those of the egg candling method. Please comment on this.

(2) When constructing the training dataset for deep learning, the authors downsampled all images from 2300×2300 to 256×256, which may result in the loss of vascular structure details. Please evaluate the impact of downsampling on vascular structure details.

(3) The advantage of laser speckle imaging lies not in structural imaging but in hemodynamic and perfusion imaging, such as capturing the periodic changes in blood flow caused by heartbeats. Please comment on whether hemodynamics can assist in non-invasive sexing of early chick embryos in intact eggs.

Reviewer #3: This paper “Exploring non-invasive sexing of early chick embryos in intact eggs using Laser Speckle Contrast Imaging (LSCI) and Deep Neural Network (DNN)” uses a custom laser speckle contrast imaging (LSCI) system to non-invasively capture extraembryonic blood vessel images from 1,251 chick embryos at days 3 and 4 of incubation, then applies ResNetBiT and YOLOv5 deep neural networks to determine whether sex can be classified from vascular patterns alone, concluding via five-fold cross-validation that neither model achieves practically useful accuracy (59–61% and 53–55% respectively), rendering the approach insufficient for reliable early sex identification.

The topic is justified. The paper could be further improved if the following remarks are taken into consideration:

1. The best-performing model (ResNetBiT at day 4) achieves only 61% accuracy, which is barely above chance — and only one-fold across both models and both days even marginally passes the significance threshold (p ≤ 0.04), making the entire DNN component statistically inconclusive and of no practical value.

2. Both models exhibit severe overfitting, with training accuracy reaching 75–100% while validation accuracy stagnates near chance level, yet no effective regularization strategies beyond early stopping are explored or discussed.

3. Using YOLOv5 — an object detection architecture — for binary classification without bounding-box labels or region proposals, is a fundamental architectural mismatch that is inadequately justified and likely contributes to its below-random performance at day 4 (53%).

4. The initial model selection procedure used a simple train/test split rather than cross-validation, and ResNetBiT was selected despite having the lowest accuracy (51%), solely based on loss behavior, which is a subjective and unreliable selection criterion.

5. The ResNet50 and YOLOv8 validation mentioned in Section 2.3 is described in a single paragraph with no quantitative results, no tables, and no methodology detail, rendering it scientifically uninformative and insufficient to constitute independent validation.

6. The paper cannot explain the 24–32 percentage point accuracy gap between its results and those of Ref. [13] using a comparable protocol and dataset size, and the inability to access Ref. [13]'s dataset or code means this critical discrepancy remains entirely unresolved.

7. The heart-detection heuristic — identifying the embryonic heart as the brightest 30×30 pixel region — is not validated against ground truth anatomical positions, and misidentification would systematically misalign all downstream crops, corrupting the entire dataset.

8. The number of images per egg is non-uniform and largely undisclosed, and the paper's claim that this has "little impact on model performance" due to transfer learning is asserted without any supporting evidence or sensitivity analysis.

9. The data availability statement contradicts PLOS ONE policy by restricting access to data "upon reasonable requests from the corresponding author" rather than making it publicly available at the time of publication.

10. The conclusion proposes CAM-based imaging from day 6 onward as a future direction yet acknowledges the current LSCI system is limited to day 5.5, meaning the most scientifically promising hypothesis cannot be tested without a hardware redesign that is not described or planned.

11. No feature visualization, saliency mapping, or interpretability analysis is provided, leaving it entirely unclear whether the models are responding to biologically meaningful vascular features or to imaging artefacts such as eggshell staining, embryo positioning, or brightness variation.

12. The paper frames itself as an exploratory study yet draws definitive conclusions ("sex identification from LSCI images at this early incubation stage is likely not feasible") from null results obtained with potentially suboptimal models on a modality that has not been fully optimized, overstating the negative conclusion beyond what the evidence supports.

Reviewer #4: Major Comments:

1. The central assumption that vascular patterns encode sex information at day 3-4 is is biologically unlikely. Authors should explicitly discuss developmental biology constraints earlier in the introduction.

2. Comparison with prior study is incomplete [e.g.: strongly contrasts its results with Ref. [13] (~85% accuracy) but does not investigate why.]

3. Statistical analysis needs improvement (e.g.: The use of binomial p-values for model accuracy)

4. PLOS ONE data policy requires open data. Deposit dataset in a public repository (Zenodo / Dryad / Figshare).

5. Limited exploration of alternative signals: The paper examines only static vascular morphology. LSCI measures blood flow dynamics, so authors should evaluate temporal features, not only spatial patterns.

6. The discussion should emphasize - biological limitations likely explain failure rather than model limitations.

7. Overfitting concerns: Training accuracy approached 75–100% while validation accuracy hovered near random chance (50–61%).

Minor Comments:

1. Several grammartical issues and typographical errors.

2. Some figure references are inconsistent.

3. The preprocessing figure could be clearer.

4. Some citations are outdated regarding chick sexing technologies.

5. Table numbering could be improved.

**********

6. PLOS authors have the option to publish the peer review history of their article (what does this mean?). If published, this will include your full peer review and any attached files.

If you choose “no”, your identity will remain anonymous but your review may still be made public.

**Do you want your identity to be public for this peer review?**   For information about this choice, including consent withdrawal, please see our Privacy Policy.

Reviewer #1: No

Reviewer #2: **Yes:**   Peng Miao

Reviewer #3: **Yes:**   Ghulam Gilanie

Reviewer #4: **Yes:**   Dr. Biraja Ghoshal

**********

[NOTE: If reviewer comments were submitted as an attachment file, they will be attached to this email and accessible via the submission site. Please log into your account, locate the manuscript record, and check for the action link "View Attachments". If this link does not appear, there are no attachment files.]

To ensure your figures meet our technical requirements, please review our figure guidelines: https://journals.plos.org/plosone/s/figures

You may also use PLOS’s free figure tool, NAAS, to help you prepare publication quality figures: https://journals.plos.org/plosone/s/figures#loc-tools-for-figure-preparation.

NAAS will assess whether your figures meet our technical requirements by comparing each figure against our figure specifications.

# Author comment: Author response to Decision Letter 1

**DOI:** 10.1371/journal.pone.0323847.r002

25 Apr 2026

See attached Response to Reviewers or Cover Letter document.

Attachment Submitted filename: Response to Reviewers.pdf

# Peer review: Decision Letter 1

**DOI:** 10.1371/journal.pone.0323847.r003

**Authors:** Khan Bahadar Khan

14 May 2026

PONE-D-25-20406R1

Detection of non-invasive sexing of early chick embryos in intact eggs using laser speckle contrast imaging and deep neural networks

PLOS One

Dear Dr. Mahler,

Thank you for submitting your manuscript to PLOS ONE. After careful consideration, we feel that it has merit but does not fully meet PLOS ONE’s publication criteria as it currently stands. Therefore, we invite you to submit a revised version of the manuscript that addresses the points raised during the review process.

Please submit your revised manuscript by Jun 28 2026 11:59PM. If you will need more time than this to complete your revisions, please reply to this message or contact the journal office at plosone@plos.org. When you're ready to submit your revision, log on to https://www.editorialmanager.com/pone/ and select the 'Submissions Needing Revision' folder to locate your manuscript file.

Please include the following items when submitting your revised manuscript:

- A letter that responds to each point raised by the academic editor and reviewer(s). You should upload this letter as a separate file labeled 'Response to Reviewers'.
- A marked-up copy of your manuscript that highlights changes made to the original version. You should upload this as a separate file labeled 'Revised Manuscript with Track Changes'.
- An unmarked version of your revised paper without tracked changes. You should upload this as a separate file labeled 'Manuscript'.

If you would like to make changes to your financial disclosure, please include your updated statement in your cover letter. Guidelines for resubmitting your figure files are available below the reviewer comments at the end of this letter.

If applicable, we recommend that you deposit your laboratory protocols in protocols.io to enhance the reproducibility of your results. Protocols.io assigns your protocol its own identifier (DOI) so that it can be cited independently in the future. For instructions see: https://journals.plos.org/plosone/s/submission-guidelines#loc-laboratory-protocols. Additionally, PLOS ONE offers an option for publishing peer-reviewed Lab Protocol articles, which describe protocols hosted on protocols.io. Read more information on sharing protocols at https://plos.org/protocols?utm_medium=editorial-email&utm_source=authorletters&utm_campaign=protocols.

As the corresponding author, your ORCID iD is verified in the submission system and will appear in the published article. PLOS supports the use of ORCID, and we encourage all coauthors to register for an ORCID iD and use it as well. Please encourage your coauthors to verify their ORCID iD within the submission system before final acceptance, as unverified ORCID iDs will not appear in the published article. *Only*   the individual author can complete the verification step; PLOS staff *cannot*   verify ORCID iDs on behalf of authors.

We look forward to receiving your revised manuscript.

Kind regards,

Khan Bahadar Khan, Ph.D

Academic Editor

PLOS One

Journal Requirements:

If the reviewer comments include a recommendation to cite specific previously published works, please review and evaluate these publications to determine whether they are relevant and should be cited. There is no requirement to cite these works unless the editor has indicated otherwise.

Please review your reference list to ensure that it is complete and correct. If you have cited papers that have been retracted, please include the rationale for doing so in the manuscript text, or remove these references and replace them with relevant current references. Any changes to the reference list should be mentioned in the rebuttal letter that accompanies your revised manuscript. If you need to cite a retracted article, indicate the article’s retracted status in the References list and also include a citation and full reference for the retraction notice.

[Note: HTML markup is below. Please do not edit.]

Reviewers' comments:

Reviewer's Responses to Questions

**Comments to the Author**

1. If the authors have adequately addressed your comments raised in a previous round of review and you feel that this manuscript is now acceptable for publication, you may indicate that here to bypass the “Comments to the Author” section, enter your conflict of interest statement in the “Confidential to Editor” section, and submit your "Accept" recommendation.

Reviewer #1: All comments have been addressed

Reviewer #2: All comments have been addressed

Reviewer #3: All comments have been addressed

Reviewer #4: All comments have been addressed

**********

2. Is the manuscript technically sound, and do the data support the conclusions?

The manuscript must describe a technically sound piece of scientific research with data that supports the conclusions. Experiments must have been conducted rigorously, with appropriate controls, replication, and sample sizes. The conclusions must be drawn appropriately based on the data presented.

Reviewer #1: Yes

Reviewer #2: Yes

Reviewer #3: Yes

Reviewer #4: Yes

**********

3. Has the statistical analysis been performed appropriately and rigorously?

Reviewer #1: Yes

Reviewer #2: Yes

Reviewer #3: Yes

Reviewer #4: Yes

**********

4. Have the authors made all data underlying the findings in their manuscript fully available?

The PLOS Data policy requires authors to make all data underlying the findings described in their manuscript fully available without restriction, with rare exception (please refer to the Data Availability Statement in the manuscript PDF file). The data should be provided as part of the manuscript or its supporting information, or deposited to a public repository. For example, in addition to summary statistics, the data points behind means, medians and variance measures should be available. If there are restrictions on publicly sharing data—e.g. participant privacy or use of data from a third party—those must be specified.

Reviewer #1: Yes

Reviewer #2: Yes

Reviewer #3: Yes

Reviewer #4: Yes

**********

5. Is the manuscript presented in an intelligible fashion and written in standard English?

PLOS ONE does not copyedit accepted manuscripts, so the language in submitted articles must be clear, correct, and unambiguous. Any typographical or grammatical errors should be corrected at revision, so please note any specific errors here.

Reviewer #1: Yes

Reviewer #2: Yes

Reviewer #3: Yes

Reviewer #4: Yes

**********

6. Review Comments to the Author

Please use the space provided to explain your answers to the questions above. You may also include additional comments for the author, including concerns about dual publication, research ethics, or publication ethics. (Please upload your review as an attachment if it exceeds 20,000 characters)

Reviewer #1: This article has relatively high novelty regarding sex detection in eggs, which is a topic that many researchers actively investigate. However, the presentation and several related details require greater attention.

I believe this article has undergone revision for a very long time, and I am not sure what caused the delay. Despite the many improvements, several aspects still require clarification.

The following are my minor revisions:

1. Please provide the dataset link through a cloud-based repository that supports DOI assignment or use a national scientific data repository facility from an institutional source, such as a national data repository. Please consider this carefully.

2. The URL for the GitHub repository can be included; however, it may be better to keep this available only upon request from the corresponding author.

Other than that, I have no further comments.

Reviewer #2: The authors have addressed all the comments from the reviewers. This manuscript is now scientifically sound, meticulously documented, and ethically transparent. It fully meets the publication criteria for PLOS ONE. I recommend accepting the manuscript in its current form without the need for further revisions.

Reviewer #3: (No Response)

Reviewer #4: Substantial revisions have been made to the manuscript including to comply with the formatting and style requirements and to report statistical significance.

**********

7. PLOS authors have the option to publish the peer review history of their article (what does this mean?). If published, this will include your full peer review and any attached files.

If you choose “no”, your identity will remain anonymous but your review may still be made public.

**Do you want your identity to be public for this peer review?**   For information about this choice, including consent withdrawal, please see our Privacy Policy.

Reviewer #1: **Yes:**   Mohammad Miftakhus Sholikin

Reviewer #2: **Yes:**   Peng MIAO

Reviewer #3: **Yes:**   Ghulam Gilanie

Reviewer #4: **Yes:**   Dr. Biraja Ghoshal

**********

[NOTE: If reviewer comments were submitted as an attachment file, they will be attached to this email and accessible via the submission site. Please log into your account, locate the manuscript record, and check for the action link "View Attachments". If this link does not appear, there are no attachment files.]

To ensure your figures meet our technical requirements, please review our figure guidelines: https://journals.plos.org/plosone/s/figures

You may also use PLOS’s free figure tool, NAAS, to help you prepare publication quality figures: https://journals.plos.org/plosone/s/figures#loc-tools-for-figure-preparation.

NAAS will assess whether your figures meet our technical requirements by comparing each figure against our figure specifications.

# Author comment: Author response to Decision Letter 2

**DOI:** 10.1371/journal.pone.0323847.r004

22 May 2026

See attached PDF file: "Response to Reviewers"

Attachment Submitted filename: Response_to_Reviewers_auresp_2.pdf

# Peer review: Decision Letter 2

**DOI:** 10.1371/journal.pone.0323847.r005

**Authors:** Khan Bahadar Khan

10 Jun 2026

Detection of non-invasive sexing of early chick embryos in intact eggs using laser speckle contrast imaging and deep neural networks

PONE-D-25-20406R2

Dear Dr. Mahler,

We’re pleased to inform you that your manuscript has been judged scientifically suitable for publication and will be formally accepted for publication once it meets all outstanding technical requirements.

Within one week, you’ll receive an e-mail detailing the required amendments. When these have been addressed, you’ll receive a formal acceptance letter and your manuscript will be scheduled for publication.

An invoice will be generated when your article is formally accepted. Please note, if your institution has a publishing partnership with PLOS and your article meets the relevant criteria, all or part of your publication costs will be covered. Please make sure your user information is up-to-date by logging into Editorial Manager at Editorial Manager® and clicking the ‘Update My Information' link at the top of the page. For questions related to billing, please contact billing support.

If your institution or institutions have a press office, please notify them about your upcoming paper to help maximize its impact. If they’ll be preparing press materials, please inform our press team as soon as possible -- no later than 48 hours after receiving the formal acceptance. Your manuscript will remain under strict press embargo until 2 pm Eastern Time on the date of publication. For more information, please contact onepress@plos.org.

Kind regards,

Khan Bahadar Khan, Ph.D

Academic Editor

PLOS One

Additional Editor Comments (optional):

Reviewers' comments:

Reviewer's Responses to Questions

**Comments to the Author**

1. If the authors have adequately addressed your comments raised in a previous round of review and you feel that this manuscript is now acceptable for publication, you may indicate that here to bypass the “Comments to the Author” section, enter your conflict of interest statement in the “Confidential to Editor” section, and submit your "Accept" recommendation.

Reviewer #1: All comments have been addressed

Reviewer #2: All comments have been addressed

Reviewer #3: All comments have been addressed

**********

2. Is the manuscript technically sound, and do the data support the conclusions?

The manuscript must describe a technically sound piece of scientific research with data that supports the conclusions. Experiments must have been conducted rigorously, with appropriate controls, replication, and sample sizes. The conclusions must be drawn appropriately based on the data presented.

Reviewer #1: Yes

Reviewer #2: Yes

Reviewer #3: Yes

**********

3. Has the statistical analysis been performed appropriately and rigorously?

Reviewer #1: Yes

Reviewer #2: Yes

Reviewer #3: Yes

**********

4. Have the authors made all data underlying the findings in their manuscript fully available?

The PLOS Data policy requires authors to make all data underlying the findings described in their manuscript fully available without restriction, with rare exception (please refer to the Data Availability Statement in the manuscript PDF file). The data should be provided as part of the manuscript or its supporting information, or deposited to a public repository. For example, in addition to summary statistics, the data points behind means, medians and variance measures should be available. If there are restrictions on publicly sharing data—e.g. participant privacy or use of data from a third party—those must be specified.

Reviewer #1: Yes

Reviewer #2: Yes

Reviewer #3: Yes

**********

5. Is the manuscript presented in an intelligible fashion and written in standard English?

PLOS ONE does not copyedit accepted manuscripts, so the language in submitted articles must be clear, correct, and unambiguous. Any typographical or grammatical errors should be corrected at revision, so please note any specific errors here.

Reviewer #1: Yes

Reviewer #2: Yes

Reviewer #3: Yes

**********

6. Review Comments to the Author

Please use the space provided to explain your answers to the questions above. You may also include additional comments for the author, including concerns about dual publication, research ethics, or publication ethics. (Please upload your review as an attachment if it exceeds 20,000 characters)

Reviewer #1: Congratulations on your work and the effort invested in completing this study. The manuscript is well prepared, and the results are presented clearly and logically. I have no further comments or suggestions for improvement at this time. I believe the manuscript is suitable in its current form and wish the authors success with publication.

Reviewer #2: All comments are addressed. I recommend accepting the manuscript in its current form without the need for further revisions.

Reviewer #3: My all comments have been addressed successfully by the authors, therefore, I would suggest acceptance of the draft.

**********

7. PLOS authors have the option to publish the peer review history of their article (what does this mean?). If published, this will include your full peer review and any attached files.

If you choose “no”, your identity will remain anonymous but your review may still be made public.

**Do you want your identity to be public for this peer review?**   For information about this choice, including consent withdrawal, please see our Privacy Policy.

Reviewer #1: **Yes:**   Mohammad Miftakhus Sholikin

Reviewer #2: No

Reviewer #3: **Yes:**   Ghulam Gilanie

**********

# Editor report: Acceptance letter

**DOI:** 10.1371/journal.pone.0323847.r006

**Authors:** Khan Bahadar Khan

PONE-D-25-20406R2

PLOS One

Dear Dr. Mahler,

I'm pleased to inform you that your manuscript has been deemed suitable for publication in PLOS One. Congratulations! Your manuscript is now being handed over to our production team.

At this stage, our production department will prepare your paper for publication. This includes ensuring the following:

* All references, tables, and figures are properly cited

* All relevant supporting information is included in the manuscript submission,

* There are no issues that prevent the paper from being properly typeset

You will receive further instructions from the production team, including instructions on how to review your proof when it is ready. Please keep in mind that we are working through a large volume of accepted articles, so please give us a few days to review your paper and let you know the next and final steps.

Lastly, if your institution or institutions have a press office, please let them know about your upcoming paper now to help maximize its impact. If they'll be preparing press materials, please inform our press team within the next 48 hours. Your manuscript will remain under strict press embargo until 2 pm Eastern Time on the date of publication. For more information, please contact onepress@plos.org.

You will receive an invoice from PLOS for your publication fee after your manuscript has reached the completed accept phase. If you receive an email requesting payment before acceptance or for any other service, this may be a phishing scheme. Learn how to identify phishing emails and protect your accounts at https://explore.plos.org/phishing.

If we can help with anything else, please email us at customercare@plos.org.

Thank you for submitting your work to PLOS ONE and supporting open access.

Kind regards,

PLOS ONE Editorial Office Staff

on behalf of

Dr. Khan Bahadar Khan

Academic Editor

PLOS One
